# Multitargeted 6-Substituted Thieno[2,3-*d*]pyrimidines as Folate Receptor-Selective Anticancer Agents that Inhibit Cytosolic and Mitochondrial One-Carbon Metabolism

**DOI:** 10.1021/acsptsci.3c00020

**Published:** 2023-04-26

**Authors:** Nian Tong, Jennifer Wong-Roushar, Adrianne Wallace-Povirk, Yesha Shah, Morgan C. Nyman, Jade M. Katinas, Mathew Schneider, Carrie O’Connor, Xun Bao, Seongho Kim, Jing Li, Zhanjun Hou, Larry H. Matherly, Charles E. Dann, Aleem Gangjee

**Affiliations:** † Division of Medicinal Chemistry, Graduate School of Pharmaceutical Sciences, Duquesne University, Pittsburgh, Pennsylvania 15282, United States; ‡ Department of Chemistry, Indiana University, Bloomington, Indiana 47405, United States; § Department of Oncology, Wayne State University School of Medicine, Detroit, Michigan 48201, United States; ∥ Department of Pharmacology, Wayne State University School of Medicine, Detroit, Michigan 48201, United States; ⊥ Barbara Ann Karmanos Cancer Institute, Detroit, Michigan 48201, United States

**Keywords:** antifolate, cancer, multitargeted, one-carbon metabolism, purine biosynthesis, serine hydroxymethyl transferase 2

## Abstract

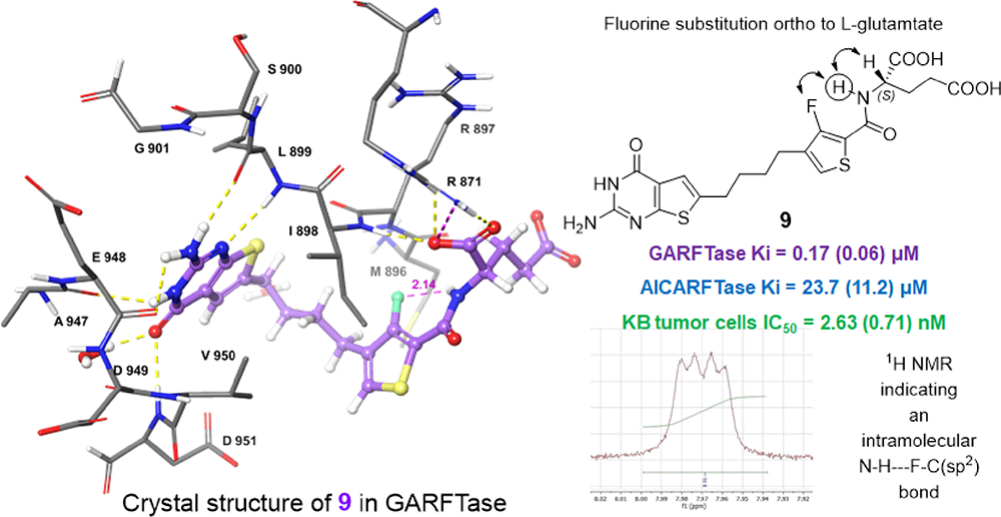

Multitargeted agents with tumor selectivity result in reduced drug resistance and dose-limiting toxicities. We report 6-substituted thieno[2,3-*d*]pyrimidine compounds (**3**–**9**) with pyridine (**3**, **4**), fluorine-substituted pyridine (**5**), phenyl (**6**, **7**), and thiophene side chains (**8**, **9**), for comparison with unsubstituted phenyl (**1**, **2**) and thiophene side chain (**10**, **11**) containing thieno[2,3-*d*]pyrimidine compounds. Compounds **3**–**9** inhibited proliferation of Chinese hamster ovary cells (CHO) expressing folate receptors (FRs) α or β but not the reduced folate carrier (RFC); modest inhibition of CHO cells expressing the proton-coupled folate transporter (PCFT) by **4**, **5**, **6**, and **9** was observed. Replacement of the side-chain 1′,4′-phenyl ring with 2′,5′-pyridyl, or 2′,5′-pyridyl with a fluorine insertion ortho to l-glutamate resulted in increased potency toward FR-expressing CHO cells. Toward KB tumor cells, **4**–**9** were highly active (IC_50_’s from 2.11 to 7.19 nM). By metabolite rescue in KB cells and *in vitro* enzyme assays, *de novo* purine biosynthesis was identified as a targeted pathway (at 5-aminoimidazole-4-carboxamide ribonucleotide formyltransferase (AICARFTase) and glycinamide ribonucleotide formyltransferase (GARFTase)). Compound **9** was 17- to 882-fold more potent than previously reported compounds **2**, **10**, and **11** against GARFTase. By targeted metabolomics and metabolite rescue, **1**, **2**, and **6** also inhibited mitochondrial serine hydroxymethyl transferase 2 (SHMT2); enzyme assays confirmed inhibition of SHMT2. X-ray crystallographic structures were obtained for **4**, **5**, **9**, and **10** with human GARFTase. This series affords an exciting new structural platform for potent multitargeted antitumor agents with FR transport selectivity.

Folate-dependent one-carbon (C1) metabolism involves interconnected folate-dependent metabolic pathways localized in the cytoplasm, mitochondria, and nucleus.^1−3^ These pathways are essential for *de novo* biosynthesis of purine nucleotides and thymidylate, interconversion of serine and glycine, and remethylation of homocysteine to methionine.^2^ Mitochondrial C1 metabolism is an essential source of glycine, glutathione, and C1 units (as formate) for nucleotide biosynthesis in the cytosol.^1,2^ As C1 metabolism is commonly dysregulated in cancer, key enzymes involved in these pathways represent promising therapeutic targets.^1−4^

*De novo* synthesis of nucleotides in proliferating cells and tissues requires C1 metabolism in which a single carbon unit is transferred from reduced folate cofactors. Inhibitors targeting C1 metabolism have been used clinically for cancer for decades and include classical antifolates such as methotrexate (MTX), pemetrexed (PMX), pralatrexate (PDX), and raltitrexed (RTX)^4,5^ (Figure 1). MTX is a dihydrofolate reductase (DHFR) inhibitor used to treat acute lymphoblastic leukemia and osteosarcoma.^4,5^ PDX is another DHFR inhibitor that is FDA approved for treating peripheral T-cell lymphoma, whereas the thymidylate synthase (TS) inhibitors RTX and PMX are used for the treatment of colorectal cancer and for treating patients diagnosed with non-small-cell lung cancer and malignant pleural mesothelioma, respectively.^4,5^ Interestingly, PMX is a multitargeted antifolate that also inhibits the folate-dependent reactions catalyzed by glycinamide ribonucleotide (GAR) formyltransferase (GARFTase) (in the trifunctional GARFTase/glycinamide ribonucleotide synthase (GARS)/aminoimidazole ribonucleotide synthetase (AIRS) enzyme) and 5-aminoimidazole-4-carboxamide ribonucleotide formyltransferase (AICARFTase) (in the bifunctional enzyme AICARFTase/inosine monophosphate cyclohydrolase (ATIC)) in *de novo* purine biosynthesis.^6−8^ Although all these antifolates are used clinically, their use is often associated with dose-limiting toxicities arising from the lack of tumor selectivity, as well as emergence of drug resistance.^4,5,9^

**Figure 1:**
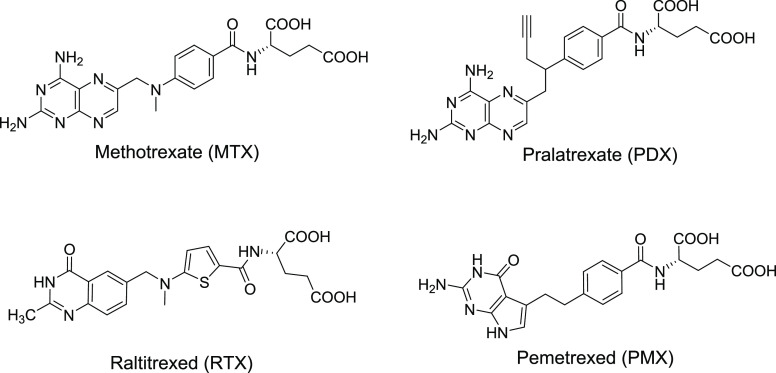
Structures of select clinically used antifolate agents.

Reflecting their ionic character, classic antifolates like the natural folates cannot diffuse across cell membranes and must be actively transported. Major transport systems for folates and related molecules include the reduced folate carrier (RFC) (SLC19A1), folate receptors (FRs) α and β, and the proton-coupled folate transporter (PCFT) (SLC46A1).^9−11^ RFC is ubiquitously expressed in both normal and malignant cells and is the primary transport mechanism for folates in cells and tissues.^10^ RFC functions optimally at neutral pH (∼7.2–7.4).^10^ As RFC substrates, the clinically used antifolates (Figure 1) show limited selectivity for uptake by tumors over proliferative tissues (*e.g.*, bone marrow), thus contributing to dose-limiting toxicities.

FRs represent a family of membrane-anchored receptors that participate in the uptake of folate into cells by endocytosis.^12^ FRs have been increasingly recognized for their potential in cancer therapy with targeted antifolates, cytotoxic folate conjugates, and antibody–drug conjugates.^12−15^ Transport via FRα is an attractive anticancer drug delivery route owing to its overexpression in a range of tumors, including breast, cervical, colorectal, renal, nasopharyngeal, ovarian, and endometrial cancers.^12,13,15^ Although FRα is also expressed in normal epithelial tissues (i.e., mammary ducts, lungs, kidneys, and the choroid plexus), in contrast to tumors, FRα in normal tissues is localized to apical membranes and is not accessible to the systemic circulation.^12^ FRβ is primarily expressed in hematopoietic tissues, activated myeloid cells, and tumor-associated macrophages (TAMs).^14−16^ FRβ expression in TAMs has prompted substantial interest in targeting this important immune population with FR-targeted agents as a cancer therapy.^14−16^ PCFT shows a limited tissue distribution but is widely expressed in human tumors and exhibits optimal transport activity at acidic pHs typical of the tumor microenvironment.^11,17^ Interestingly, PCFT is also expressed with FRβ in M2-like macrophages.^18^

Resistance to cancer therapy remains a formidable challenge, and the potential of multitargeted therapy for cancer is well established.^19,20^ Indeed, growing evidence suggests that drugs capable of inhibiting multiple targets simultaneously could afford the benefits of drug combinations in single agents.^21−24^

We previously discovered a series of novel cytotoxic agents **1** and **2** with a 6-substituted thieno[2,3-*d*]pyrimidine scaffold (Figure 2). These agents selectively target cells (including tumor cells) expressing FRα or FRβ while not affecting cells that express RFC or PCFT.^25^ These analogs are structurally and functionally distinct from PMX, a 5-substituted pyrrolo[2,3-*d*]pyrimidine antifolate,^6^ as well as previously reported 6-substituted pyrrolo[2,3-*d*]pyrimidine antifolates.^26−30^ Isosteric replacement of the fused pyrrole ring with a thiophene ring provides an increase in the ring size that more closely resembles the 6–6 fused pteridine ring system of naturally occurring folate cofactors. In addition, replacing the pyrrole NH by the S in the thiophene tests the significance of a hydrogen bond donor (NH) versus a hydrogen bond acceptor (S) in target engagement.^31^ More recently, we discovered thieno[2,3-*d*]pyrimidine compounds **10** and **11** with a 2′,4′-thiophene replacement of the side-chain 1′,4′-phenyl ring in **1** and **2** (Figure 2).^32^

**Figure 2:**
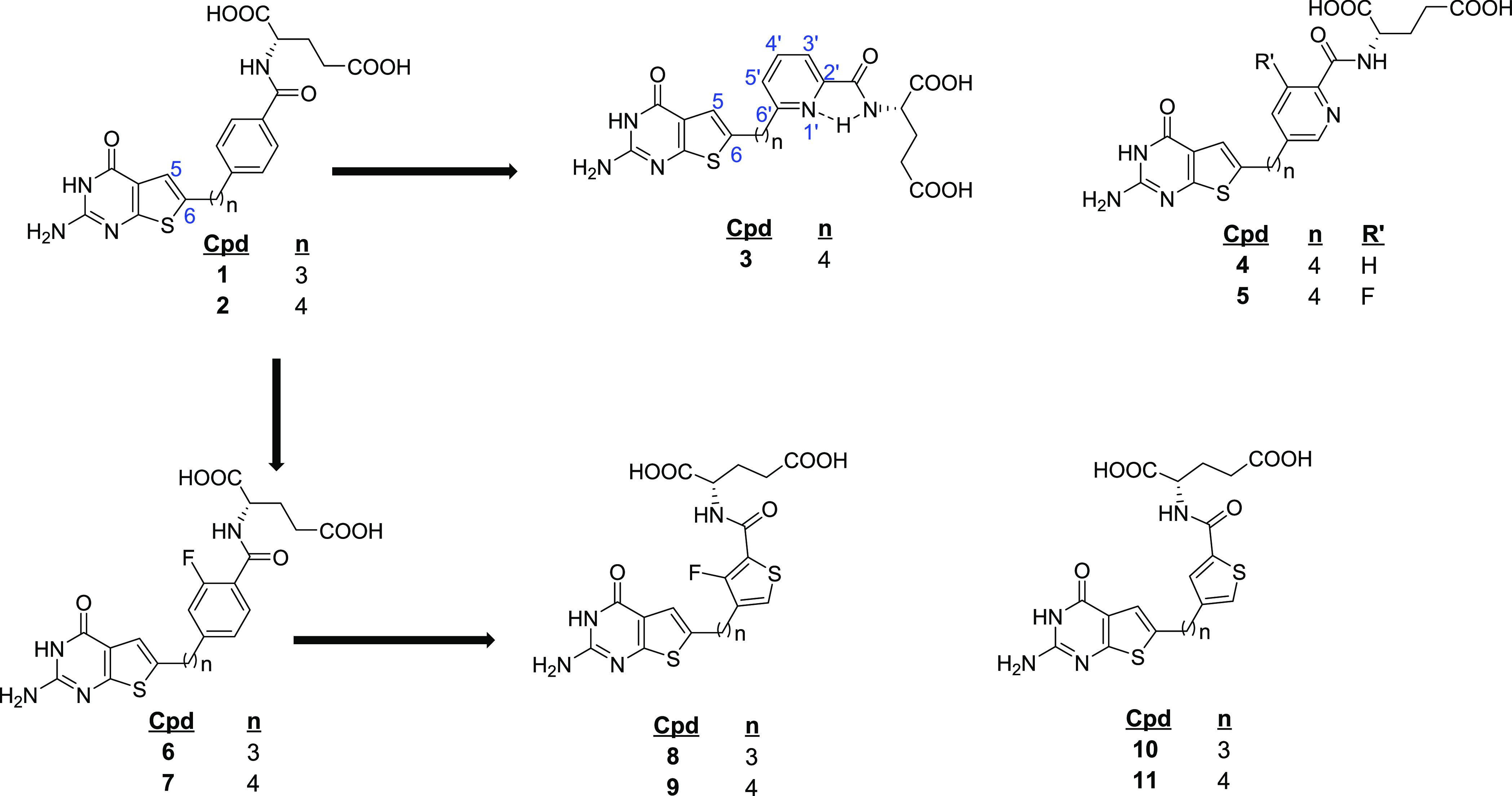
Structures of analogs with 6-substituted thieno[2,3-*d*]pyrimidine.

The mechanism of tumor cell inhibition by these novel 6-substituted thieno[2,3-*d*]pyrimidine compounds is also distinct from that by the 6-substituted pyrrolo[2,3-*d*]pyrimidine antifolates, which are exclusively inhibitors of GARFTase.^26−30^ Indeed, we established that both GARFTase and AICARFTase in *de novo* purine nucleotide biosynthesis are important intracellular targets for thieno[2,3-*d*]pyrimidine compounds **1**, **2**, **10**, and **11**.^25,32^ The near-absolute selectivity of this series for internalization by FRs over RFC and their dual inhibition of GARFTase and AICARFTase make these promising leads for the design and optimization of tumor-selective multitargeted anticancer agents.

In this report, we further optimize the selectivity of the thieno[2,3-*d*]pyrimidine series of compounds for transport (e.g., FRα and FRβ versus RFC) while preserving their multitargeted inhibition and antitumor activity by conformational restriction and/or by structurally modifying ligand–enzyme interactions. Thus, bioisosteric replacement of the side-chain 1′,4′-phenyl ring by pyridine regioisomers resulted in target compounds **3** and **4** (Figure 2). Introduction of nitrogen in the phenyl ring as a 2′,6′-pyridine (**3**) afforded a potential intramolecular H-bond between the pyridine nitrogen and the hydrogen on the adjacent amide NH, leading to conformational restriction of the side chain (Figure 2).^33,34^ Further, regiosubstitutions on the pyridyl ring system involving the 4-carbon bridge (**4** and **5**) were synthesized and evaluated to determine their inhibitions of multiple targets in C1 metabolism and to increase inhibitory potency and preserve transport selectivity over RFC. The pyridine ring can also form noncovalent interactions such as H-bonds, π–π interactions, and OH/NH interactions with amino acid residues in the binding pockets of putative cellular targets, which could further enhance potencies over the phenyl ring analogs **1** and **2**.^33,34^

An additional important goal of our study was to determine the impact of fluorine substitutions in the side-chain aromatic moiety. Fluorine substitutions in drug design and development have important applications, reflecting fluorine’s unique properties.^35^ Thus, introducing fluorine into a molecule alters the steric effects, lipophilicity, and electronics of the system and productively impacts parameters such as p*K*_a_, membrane permeability, selectivity, potency, and ADMET.^35,36^ Introducing fluorine adjacent to the side-chain amide of l-glutamate improved the potencies of classical antifolates related to the pyrrolo[2,3-*d*]pyrimidine series.^28^ We posited that this could be a result of a possible hydrogen bond of fluorine with the amide NH on the l-glutamate moiety, providing conformational restriction.^28^ Thus, in addition to replacing the 1′,4′-phenyl ring by a 2′,5′-pyridyl ring in compound **4**, we introduced an ortho fluorine in the 2′,5′-pyridyl ring to afford compound **5**. Finally, to further explore the influence of strategic fluorine substitutions on transport selectivity, enzyme inhibition, and antitumor activity, we designed fluorinated, 6-subsituted thieno[2,3-*d*]pyrimidine compounds with both 5- and 6-member rings in the side-chain (**6**–**9**) as potentially selective multitargeted antitumor agents (Figure 2).

Collectively, these novel series of analogs permit systematic comparison of the influence of these structural modifications on biological activities including the impact on selective membrane transport, target inhibition, and antitumor activity. Reflecting the favorable interactions imparted by the pyridyl group and the fluorine substitutions, we hypothesize that a 6-substituted thieno[2,3-*d*]pyrimidine linked to a pyridyl l-glutamate side-chain (exemplified by **3** and **4**) and a fluorine atom ortho to the l*-*glutamate moiety (exemplified in **5**–**9**) will increase inhibition of FR-expressing tumors while preserving transport selectivity toward FRs over RFC.

## Rationale for Proposed Compounds: Molecular Modeling

**Figure 3:**
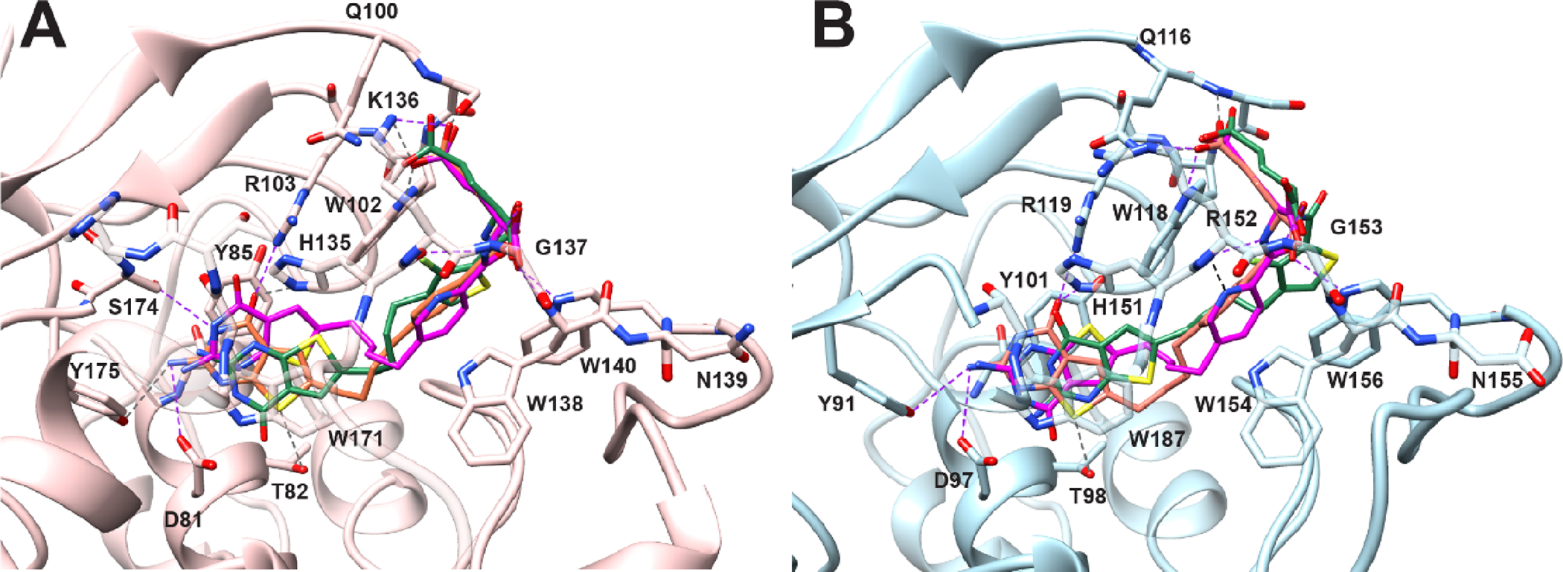
Molecular modeling studies with human FRα (PDB: 5IZQ) and FRβ (4KN2). The docked poses of the lead compound **2** (orange, docking score: −14.62 kcal/mol for FRα and −15.14 kcal/mol for FRβ), pyridine analog **4** (magenta, **–**13.68 kcal/mol for FRα and −12.77 kcal/mol for FRβ), and fluorinated analog **9** (green, −14.14 kcal/mol for FRα and −12.70 kcal/mol for FRβ) with the crystal structures for FRα (PDB 5IZQ)^27^ and FRβ (PDB 4KN2)^37^ are shown in panels A and B, respectively. The docking studies indicate that all three compounds were able to fit a similar space as the crystallized ligand in 5IZQ^27^ for FRα and in 4KN2^37^ for FRβ. Docked poses of the 2′,5′-substituted pyridine analog **4**, the ortho fluorinated analog **9**, and its parent des-fluoro phenyl bridge compound **2** were superimposed in the crystal structure of FRα and FRβ. Conserved polar interactions with the crystal structure are shown as purple dashed lines, whereas unique interactions are shown as gray dashed lines. The docking studies were carried out using Maestro 12.3 (Schrödinger LLC), and the results were visualized in Chimera visualization software.

**Figure 4:**
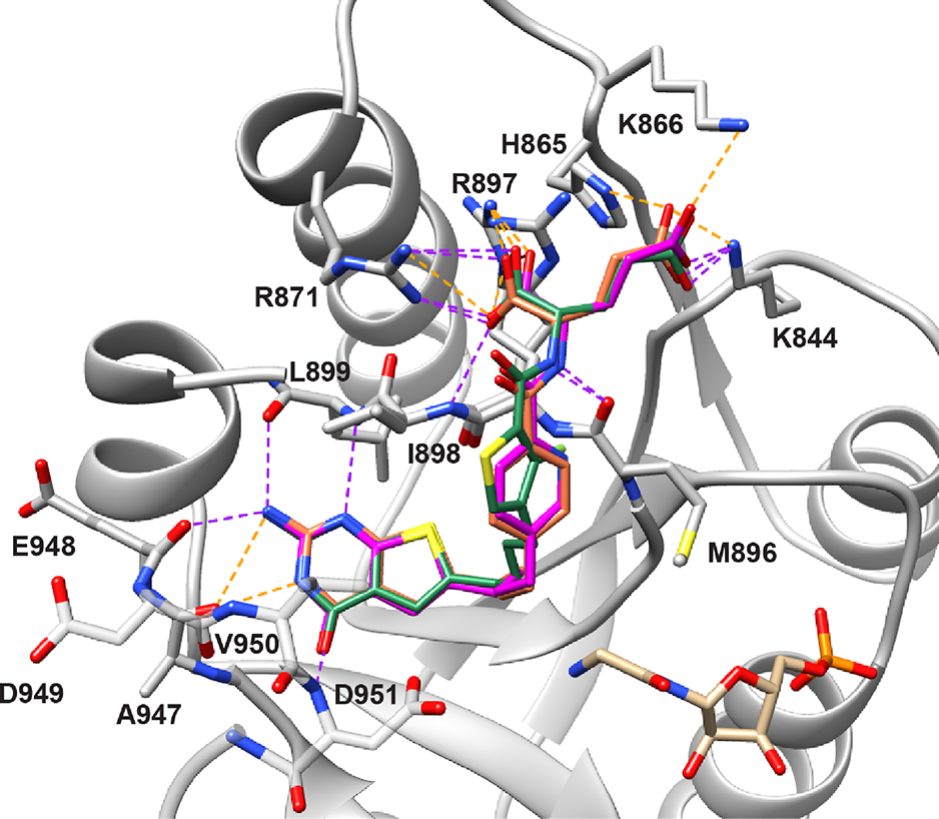
Molecular modeling studies with human GARFTase (PDB: 7JG0).^32^ The docked poses of the lead compound **2** (orange, docking score: −15.60 kcal/mol), pyridine analog **4** (magenta, −15.40 kcal/mol), and fluorinated thiophene analog **9** (green, −16.05 kcal/mol) in the folate binding pocket of GARFTase^32^ are shown. Polar interactions consistent with the crystal structure are shown as purple dashed lines, whereas interactions unique to the computational modeling are shown as orange dashed lines. The docking studies were carried out using Maestro 12.3 (Schrödinger LLC), and the results were visualized in the Chimera visualization software.

**Figure 5:**
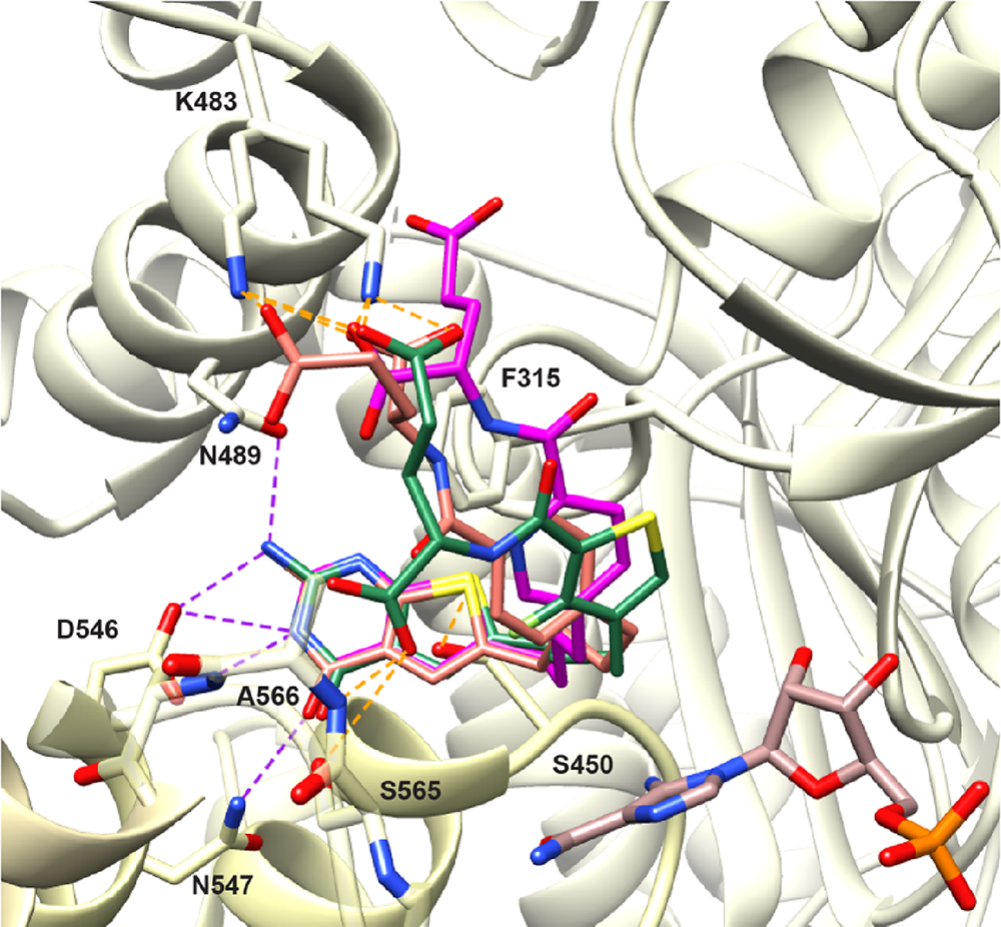
Molecular modeling studies with the AICARFTase domain of human ATIC (PDB: 1P4R).^38^ The docked poses of the lead compound **2** (orange, docking score: −12.49 kcal/mol), the pyridine analog **4** (magenta, −12.71 kcal/mol), and the fluorinated thiophene analog **9** (green, −13.51 kcal/mol) in the folate binding pocket of AICARFTase (PDB: 1P4R)^38^ are shown. Conserved polar interactions with the crystal structure are shown as purple dashed lines, whereas unique interactions of the computational docked structures are shown as orange dashed lines. The docking studies were carried out using Maestro 12.3 (Schrödinger LLC), and the results were visualized in the Chimera visualization software.

To rationalize the design and synthesis of the proposed side-chain pyridyl and thiophene analogs (**3**–**9**) versus phenyl (**1** and **2**) and thiophene (**10** and **11**) side-chain compounds,^25,32^ we performed molecular modeling studies with human FRα, FRβ, GARFTase, and AICARFTase. In this study, an induced-fit docking protocol (Schrödinger LLC) was utilized to explore binding interactions and validate our proposed drug targets using X-ray crystal structures of human FRα (5IZQ),^27^ FRβ (4KN2),^37^ GARFTase (7JG0),^32^ and ATIC (1P4R).^38^ Molecular modeling was performed in Maestro for the thieno[2,3-*d*]pyrimidine analogs (**3**–**9**) to provide a molecular rationale for the pyridine and fluorinated compounds. Results are shown for compounds **4** and **9** compared to **2** (Figures 3–5).

In the molecular docking studies (FRα), the π–π stacking interactions between the thieno[2,3–*d*]pyrimidine scaffolds of compounds **2**, **4**, and **9** and the side chains of Tyr85 and Trp171 were shown to be similar to the bicyclic scaffold of the bound pyrrolo[2,3-*d*]pyrimidine ligand in our previous report^27^ (Figure 3A; ligand not shown for clarity). For compound **4**, similar to the lead compound **2**, the 2-amino (2-NH_2_), N3, and the 4-oxo group exhibited hydrogen bonding interactions with Asp81, Ser174, and the side chains of Arg103, respectively. The designed analog **4** also participated in an additional hydrogen bond with the side chain of His135 via its 4-oxo group. The bicyclic scaffold for **9** showed slightly different hydrogen bonding with the residues. The 2-NH_2_ and N3 of the pyrimidine of compound **9** formed a hydrogen bond with Asp81 and Ser174, respectively, whereas the 4-oxo group was not able to form any hydrogen bonding interactions with the residues. The l-glutamates of the three compounds were positioned in a similar orientation with the α-carboxylates of the glutamates involved in hydrogen bonding with the backbone amides of Trp138 and Gly137 and the indole side chain of Trp140. The γ-carboxylate formed hydrogen bonds with the indole nitrogen of the side chain of Trp102 and an ionic interaction with the side chain of Lys136. The docked scores for **2**, **4**, and **9** with FRα were −14.62, −13.68, and −14.14 kcal/mol, respectively. Compounds **3** and **5**–**8** had docked scores that ranged from −13.41 to −16.26 kcal/mol (Table S1, Supporting Information).

Figure 3B displays the docked poses of compounds **2, 4**, and **9** in FRβ. The docking studies of compound **2** showed hydrogen bonding interactions of the 2-NH_2_ and 4-oxo group of the ligand with the carbonyl side chain of Asp97 and the side chains of Arg119 and His151, respectively. Further, the thieno[2,3-*d*]pyrimidine scaffold of **2** formed π–π stacking with the side chains of Tyr101 and Trp187. In contrast to the lead **2**, compound **4** only formed a hydrogen bond with the carbonyl side chain of Asp97, and compound **9** formed two hydrogen bonds with the side chains of Arg119 and His151 while losing the hydrogen bonding interaction with Asp97. The binding spaces for the l-glutamate moieties of **2**, **4**, and **9** were similar, specifically, with the α-carboxylates forming hydrogen bonds with a conserved water molecule and the backbone NH of Gly153; the γ-carboxylates engaged in salt bridges with the side chain of Arg152 as well as hydrogen bonding with the backbone NH of Ser117 and Gln116. In addition, l-glutamates of **4** and **9** were oriented with the NH of the glutamate facing the pyridine nitrogen of **4**. The ortho fluorine of **9** facilitated a pseudo 6-membered ring via an intramolecular fluorine–hydrogen bond similar to that seen in the FRα docking poses. The docked scores for **2**, **4**, and **9** with FRβ were −15.14, −12.77, and −12.70 kcal/mol, respectively. Compounds **3** and **5**–**8** had docked scores that ranged from −12.90 to −15.23 kcal/mol (Table S1, Supporting Information). Collectively, these results suggest that the proposed compounds should bind to and be transported by FRα and FRβ.

For target validation, the thieno[2,3-*d*]pyrimidine scaffold of all the three compounds (**2**, **4**, and **9**) shared a binding pocket with the bicyclic scaffold of the native ligand in the GARFTase crystal structure (Figure 4) (PDB: 7JG0,^32^ native ligand not shown for clarity). Several polar interactions stabilized the bicyclic scaffolds in the binding site, including hydrogen bonds between the 2-NH_2_ of the ligands and the backbone of Leu899 and Glu948, between the NH of the ligand and the backbone carbonyl of Ala947, and between the 4-oxo group of the ligands and the backbones of Asp951, Val946, and Gly953. The l-glutamate moieties of all ligands showed similar binding, with the α-carboxylates creating ionic interactions with Arg897 and Arg871 side chains, in addition to hydrogen bonding with the backbone amide of Ile898. The γ-carboxylates of the ligands also formed salt bridges and hydrogen bonds with the side chains of Lys866 and Lys844. The docked scores (Figure 4) of **2**, **4**, and **9** are very similar (−15.60, −15.40, and −16.05 kcal/mol, respectively) and indicate the potential for good inhibition of GARFTase. Likewise, compounds **3** and **5**–**8** had docked scores that ranged from −15.68 to −16.09 kcal/mol (Table S1, Supporting Information).

The thieno[2,3-*d*]pyrimidine compounds **2**, **4**, and **9** were also docked in the crystal structure of human ATIC (PDB:1P4R). Docked poses for compound **2**, the pyridine analog **4**, and the fluorinated analog **9** are shown in Figure 5. Analogous to the lead compound **2**, compounds **4** and **9** exhibited hydrogen bonding interactions between the 2-NH_2_, N3, and 4-oxo of the bicyclic scaffold and Asp546 and Asn489, Asp546, and Asn547, respectively. The thieno[2,3-*d*]pyrimidine bicyclic scaffolds also exhibited a π–π stacking interaction with Phe315. Interestingly, the l-glutamate moieties of the three compounds bound differently in the same binding pocket. The α-carboxylate of **2** formed a salt bridge with Lys483, contacts not seen with either **4** or **9**. The γ-carboxylates of **2**, **4**, and **9** bound in distinct conformations, but all conformations essentially maintained a salt bridge with the side chain of Lys483. Docked scores for **2**, **4**, and **9** were −12.49, −12.71, and –13.51 kcal/mol, respectively. The docked scores of **3** and **5**–**8** in ATIC ranged from −12.23 to −13.67 kcal/mol (Table S1, Supporting Information).

In summary, the docked scores of the pyridine-substituted and fluorinated analogs were either maintained or slightly improved compared to the des-fluoro lead compounds, indicating inhibition similar to **2**.^25^ The bicyclic scaffolds were stabilized in the binding pocket by multiple polar interactions as well as π–π stacking interactions, and the l-glutamate side chains were favorably oriented for improved binding with FRα and FRβ and with GARFTase and AICARFTase. All the docked poses (Figures 3A,B, 4, and 5) displayed a syn conformation between the fluorine atom and the amide NH of the l-glutamate via favorable intramolecular hydrogen bonding, although this bonding was not specifically seen in the docked structure, as NH is normally involved in hydrogen bonding with the backbone carbonyl of target proteins (Figure 3A,B in FRs). We believe that the side-chain NH orientation dictated by potential intramolecular hydrogen bonding could bias the conformational ensemble of the compound in solution toward this interaction such that the entropic penalty upon binding to target proteins is minimized.

## Results and Discussion

### Synthesis

**Scheme 1:**
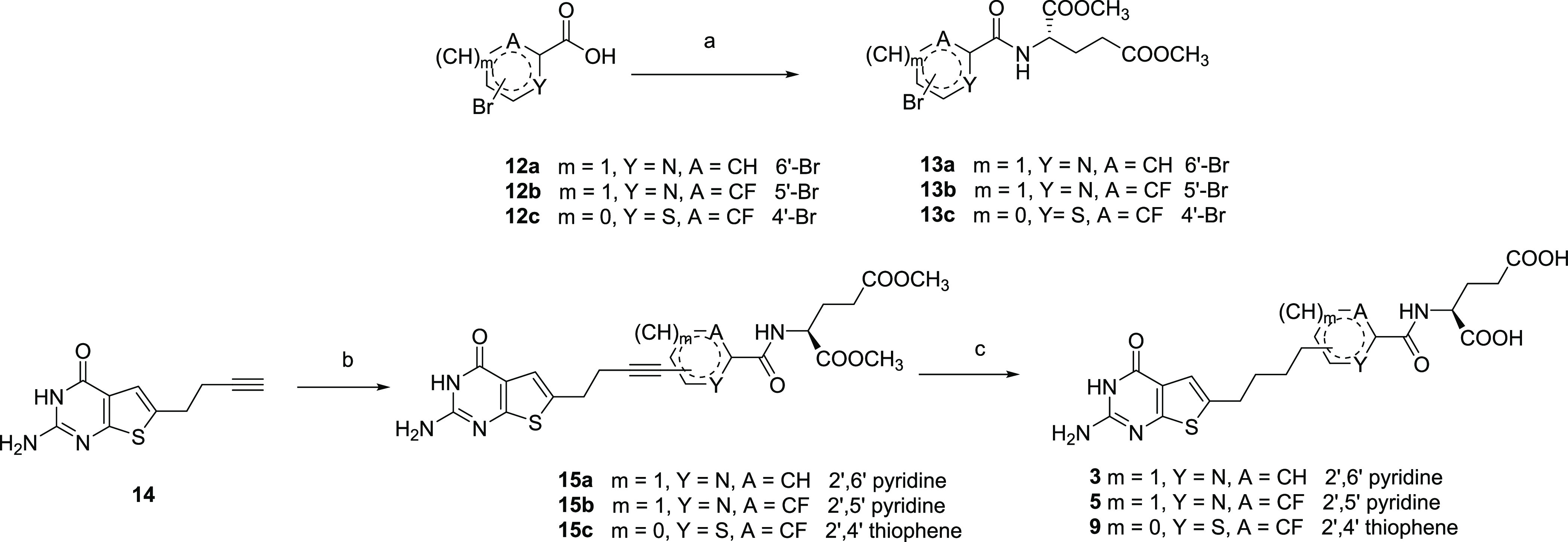
Synthesis of Target Compounds **3**, **5**, and **9** Reagents and conditions: (a) NMM, CDMT, dimethyl-l*-*glutamate hydrochloride, DMF, rt, 12 h, 45–69%; (b) Cul, tetrakis(triphenylphosphine)palladium(0), TEA, **13a-c**, DMF, 70 °C, μW, 12 h, 44–46%; and (c) (i) 10% Pd/C, H_2_, 12 h; (ii) 1 N NaOH, rt, 1 h, 46–54%.

The syntheses of target compounds **3**, **5**, and **9** are shown in Scheme 1. Using *N*-methyl morpholine (NMM) as the base and 2,4-dimethoxy-6-chlorotriazine (CDMT) as the coupling reagent, the glutamylated (hetero)aromatic bromides **13a**–**c** were obtained in yields ranging from 45−69% by an amide coupling reaction using commercially available carboxylic acids **12a**–**c** and dimethyl-l-glutamate hydrochloride, respectively. To afford the 2-amino-4-oxo-6-substituted thieno[2,3-*d*]pyrimidine alkynes **15a**–**c**, the l-glutamate bromides **13a**–**c** were subjected to Sonogashira coupling with the alkyne **14** under microwave heating; the resulting compounds were formed in 44–46% yields. Palladium-catalyzed hydrogenation of the alkynes **15a**–**c** followed by saponification of the resulting l-glutamate esters provided target compounds **3**, **5**, and **9**, respectively, in 46–54% yield over two steps.

**Scheme 2:**
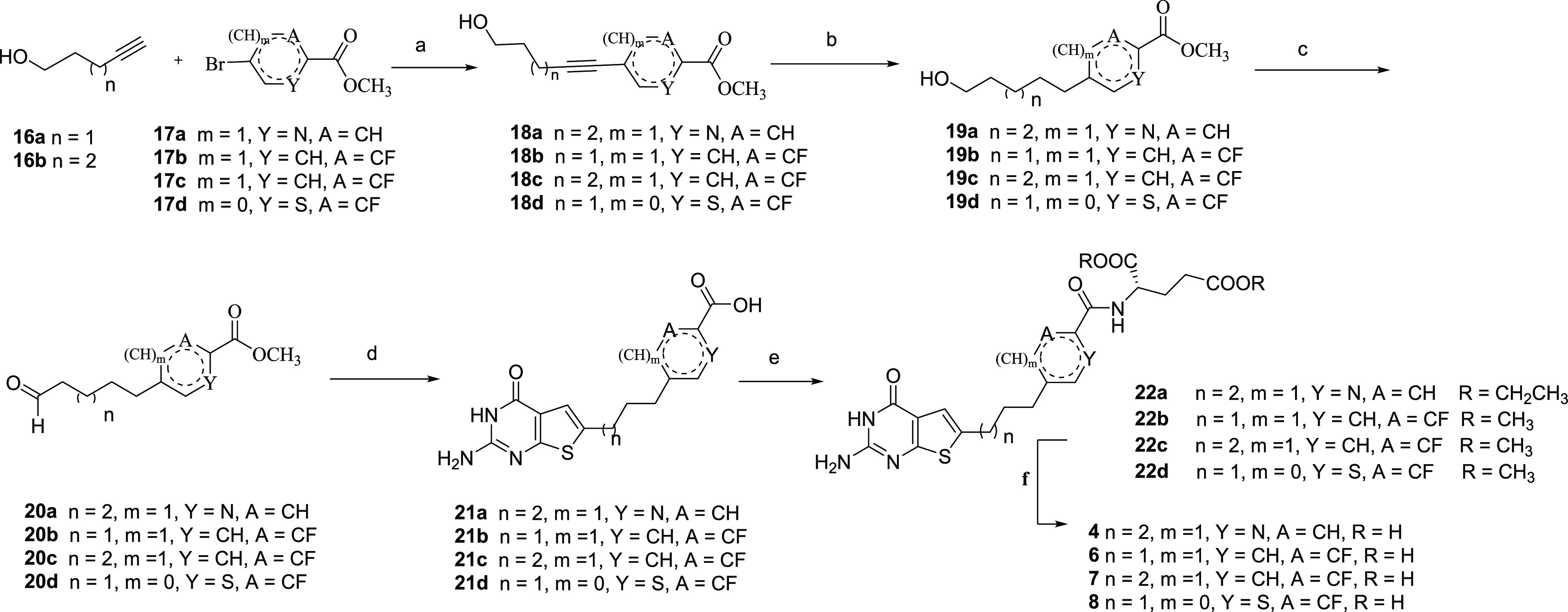
Synthesis of Target Compounds **4**, **6**, **7**, and **8** Reagents and conditions: (a) PdCl_2_, Cul, acetonitrile, PPh_3_, TEA, 4-pentyn-1-ol or 5-hexyn-1-ol, 100 °C, μW, 1 h, 66–84%; (b) H_2_, Pd/C, 55 psi, 59–90%; (c) DMP, DCM, 0 °C to rt, 74–87%; (d) (i) ethanol, TEA, ethyl cyanoacetate, sulfur powder, 100 °C, μW; (ii) DMSO_2_, chloromoformamidine hydrochloride, 120 °C, 4 h; (iii) 1 N NaOH, rt, 20–45%; (e) NMM, CDMT, diethyl-l-glutamate hydrochloride or dimethyl-l-glutamate hydrochloride, DMF, rt, 2–4 h, 44–59%; and (f) 1 N NaOH, rt, 4 h, 82–95%.

Target compounds **4**, **6**, **7**, and **8** were synthesized as shown in Scheme 2. The alkyne-coupled (hetero)aromatic ester alcohols **18a**–**d** were obtained following Sonogashira coupling of commercially available 4-pentyn-1-ol **16a** or 5-hexyn-1-ol **16b** with the appropriate bromides **17a**–**d** in 66–84% yield. By catalytic hydrogenation of the alkyne alcohols **18a**–**d**, their respective saturated alkanes **19a**–**d** were obtained in 59–90% yield. Subsequent oxidation of the alcohols **19a**–**d** using Dess–Martin periodinane (DMP) gave the corresponding aldehydes **20a**–**d** in 74–87% yield. Gewald reaction of **20a**–**d** under microwave conditions followed by cyclization with chloroformamidine hydrochloride and subsequent saponification afforded the target thieno[2,3-*d*]pyrimidine acids **21a**–**d** in 20–45% yield over three steps. Using NMM and CDMT as the coupling reagents, the peptide coupling of **21a**–**d** with l-glutamate dimethyl or diethyl ester resulted in the formation of precursor diester semisolid intermediates **22a**–**d** in 44–59% yield. Saponification of **22a**–**d** gave the respective target compounds **4**, **6**, **7**, and **8** in 82–95% yield. ^1^H NMR spectra, HPLCs, and mass spectra for synthesized final compounds **3**–**9** are provided in Figure S1 (Supporting Information).

### NMR Support as Evidence of Possible Intramolecular N–H···F–C(sp^2^) Hydrogen Bond of the Fluorinated Thiophene Analogs in the Solution State

**Figure 6:**
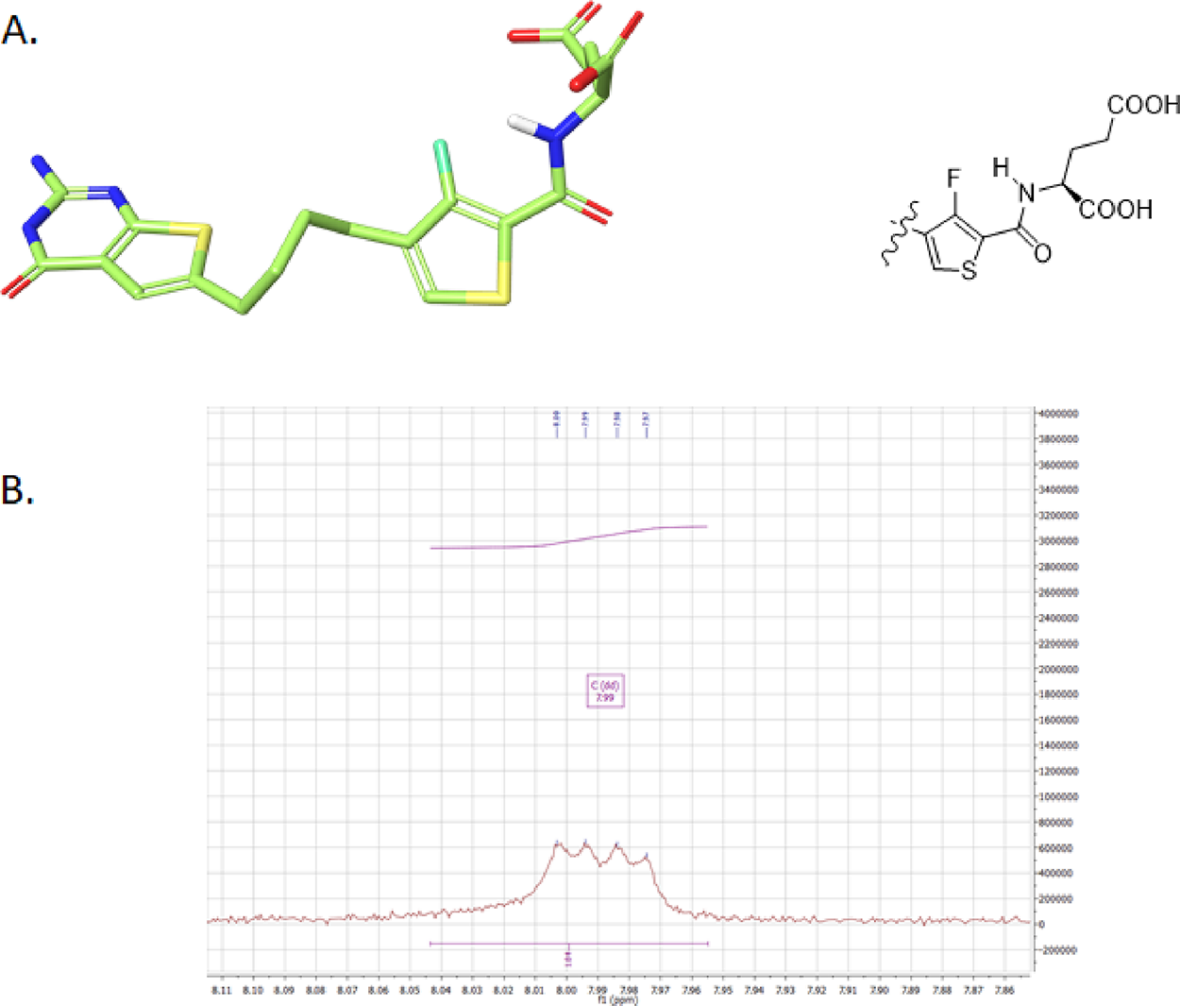
Representative example with compound **9** (green) of an energy minimization free ligand and docked pose in FRα and its NH signal by ^1^H NMR. (A) Intramolecular fluorine–hydrogen bond facilitating a syn conformation detected in the docked pose of ligand **9** in FRα using Maestro. (B) 400 MHz ^1^H NMR for the CO-NH amide hydrogen, detected as a doublet of a doublet NH peak.

All the docked poses of the fluorinated compounds (compound **9** is used as a representative example in Figure 6A) showed that the ligands were able to position the fluorine atom in a syn conformation toward the amide NH proton, which facilitated weak intramolecular hydrogen bonding with the fluorine atom. The NMR spectrum in DMSO-*d*_6_ of the fluorinated analog **9** confirmed the presence of spin–spin coupling between the fluorine atom and the NH proton of the l-glutamate amide. The ^1^H NMR of the CO-NH proton displayed a doublet of a doublet. This arises from the CO-NH proton coupling with the α-CH proton and the fluorine atom (Figure 6B) in the ^1^H NMR spectra (*J*_(H,F)_ = 3.8 Hz) that indicates nuclear spin coupling with fluorine. In comparison, for the des-fluoro compound **11**, ^1^H NMR of the CO-NH proton only displayed a doublet due to the coupling between the NH hydrogen with the adjacent α-CH. Similar coupling between an ortho fluorine atom and the amide NH proton of the side-chain l-glutamate was previously reported for the pyrrolo[2,3-*d*]pyrimidine series as an N–H···F–C(sp^2^) hydrogen bond.^28^

As previously reported,^28^ we observed that the amide NH proton of the fluorinated analogs required a significantly longer time (>1 h) for complete exchange (after D_2_O exchange for equal concentrations of the compounds dissolved in DMSO-*d_6_*) compared to the nonfluorinated analogs (<5 min). A similar observation was made for the fluorinated analog **9**, as indicated in Figure S2 (Supporting Information). This suggests that a single fluorine atom on an sp^2^ carbon can act as a hydrogen bond acceptor in a fluorine hydrogen bond. This NMR study (Figure 6 and Figure S2, Supporting Information) provides further support that the fluorine and amide NH are in syn conformation relative to each other in the solution prior to binding to target proteins.

### Biological Evaluation

#### Antiproliferative Effects of 6-Substituted Thieno[2,3-*d*]pyrimidine Antifolates in Relation to Mechanisms of Folate Transport

As an important goal of our studies was to discover novel multitargeted cytotoxic compounds with selective transport by FRs over RFC, we assessed the antiproliferative activities and transporter specificities for the new thieno[2,3-*d*]pyrimidine compounds **3**–**9** compared to previously published compounds **1**, **2**, **10**, and **11**.^25,32^ We used a unique panel of engineered Chinese hamster ovary (CHO) sublines derived from a transporter-null MTXRIIOua^R^2-4 CHO subline (R2) and designed to individually express human FRα (RT16), FRβ (D4), RFC (PC43-10), or PCFT (R2/PCFT).^39−42^ As the CHO sublines are isogenic and differ only in the presence or absence of the human folate transporters, differences in the extent of inhibition of cell proliferation between the cell lines upon treatment with the various compounds provide a robust readout of transport specificities.^25,26,29,30,43^

The CHO sublines were treated for 96 h with a range of drug concentrations up to 1000 nM; cell viabilities were measured with a fluorescence assay.^26^ Additional treatments included the classical antifolate PMX, which is transported by both RFC and PCFT with modest uptake by FRs.^4^ As a control, we used transporter-null R2 cells.^42^ To extend studies to human tumor cells, we also performed experiments in RFC-, PCFT-, and FRα-expressing KB tumor cells.^44^ To confirm the contribution of FR-mediated uptake to antitumor activity, for KB cells, we added 200 nM folic acid, which inhibits FR uptake by direct competition without effects on either RFC or PCFT.^25,26,28−30,43^ The results are summarized in Table 1.

**Table 1:** IC_50_ Values for 6-Substituted Thieno[2,3-*d*]pyrimidine Analogs and PMX with RFC-, PCFT-, and FR-Expressing Cell Lines^a^

		IC_50_ (nM)						
		RFC		FRα	FRβ	PCFT	RFC/FRα/PCFT	
compound		PC43-10	R2	RT16	D4	R2/PCFT4	KB	KB (+FA)
**1**	3C/1′4′phenyl	>1000	>1000	13.0 (3.4)	112 (12)	>1000	23 (5.5)	>1000
**2**	4C/1′4′phenyl	>1000	>1000	9 (2.9)	20 (3.9)	>1000	4.9 (1.3)	>1000
**6**	3C/2′F/1′4′phenyl	>1000	>1000	1.07 (0.47)	0.48 (0.27)	591 (76)	7.19 (0.37)	>1000
**7**	4C/2′F/1′4′phenyl	>1000	>1000	2.27 (0.54)	1.14 (0.28)	>1000	5.27 (0.27)	>1000
**10**	3C/2′4′thiophene	>1000	>1000	0.44 (0.04)	0.24 (0.06)	107 (39)	1.48 (0.04)	>1000
**11**	4C/2′4′thiophene	>1000	>1000	5.62 (1.31)	5.63 (1.63)	460 (98)	6.85 (0.99)	>1000
**8**	3C/3′F/2′4′thiophene	560 (174)	>1000	1.96 (0.60)	0.26 (0.11)	>1000	6.84 (0.07)	>1000
**9**	4C/3′F/2′4′thiophene	>1000	>1000	1.29 (0.10)	0.48 (0.07)	167 (81)	2.63 (0.71)	>1000
**3**	4C/2′6′pyridine	>1000	>1000	24.68 (8.07)	41.91 (5.86)	>1000	>1000	>1000
**4**	4C/2′5′pyridine	>1000	>1000	0.94 (0.30)	60.75 (3.10)	318 (126)	2.11 (0.13)	>1000
**5**	4C/3′F/2′5′pyridine	>1000	>1000	1.23 (0.22)	0.25 (0.01)	344 (107)	5.05 (0.07)	>1000
PMX		14 (205)	258 (44)	42 (9)	60 (8)	13.2 (2.4)	68 (12)	327 (103)

a Proliferation assays used R2, PC43-10, RT16, D4 and R2/PCFT4 CHO, and KB human tumor cells.^25,26^ Results are shown as mean IC_50_ values (± standard errors), corresponding to concentrations that inhibit growth by 50% relative to vehicle-treated control cells. The data are shown as mean values from at least three experiments. Data for PMX, **1**, **2, 10**, and **11** were published.^25,26,32^

With the exception of a slight inhibition by compound **8**, there was no effect on proliferation of RFC-expressing PC43-10 cells by the thieno[2,3-*d*]pyrimidine compounds up to 1000 nM. Although modest inhibition of PCFT-expressing R2/PCFT4 cells was detected for compounds **4**, **5**, **6**, **9**, **10**, and **11** (reflected in IC_50_s between 107 and 591 nM) (Table 1), inhibition toward RT16 (FRα) and D4 (FRβ) cells prevailed, with IC_50_ values ranging from 0.4 (**10**) to 25 nM (**3**) and from 0.24 (**10**) to 112 nM (**1**), respectively. For FRα expressing RT16 cells, IC_50_ values were in order of potency **10** > **4** ∼ **6** ∼ **5** ∼ **9** > **7** ∼ **8** > **11** > **2** > **1** > **3** (Table 1). Inhibition of FRβ-expressing D4 cells, as reflected in the IC_50_ values, exceeded that for RT16 cells (FRα) for compounds **5**, **6**, **7**, **8**, **9**, and **10**; IC_50_ values for D4 cells were in the order **10** ∼ **8** ∼ **5** > **6** ∼ **9** > **7** > **11** > **2** > **3** > **4** > **1** (Table 1). None of the thieno[2,3-*d*]pyrimidine compounds inhibited R2 cells. Although increasing the bridge lengths from 3- to 4-carbons or replacing the side-chain phenyl ring in **1** and **2** with a thiophene (**8**, **9**, **10**, and **11**) or pyridine (**3**, **4**, and **5**) resulted in notable differences in growth inhibition toward FR-expressing cells, especially striking was the impact of fluorine substitutions on the side-chain ring that dramatically increased inhibition toward both RT16 (FRα) and D4 (FRβ) cells compared to their des-fluorinated counterparts (i.e., compare compounds **6** and **7** with compounds **1** and **2**, respectively, and compound **9** versus **11** for both FRα and FRβ; also compare compound **5** versus **4** for FRβ (Table 1)).

For the active compounds, inhibition of KB tumor cells was in the order **10** ∼ **4** ∼ **9** > **2** > **5** ∼ **7** > **8** ∼ **11** ∼ **6** > **1**> > > **3** and was abolished by treatment with 200 nM folic acid, establishing the transport specificity by FRα in spite of the modest transport by PCFT for a few compounds (above).^25,26^ With the exception of compound **3**, all the compounds were superior to PMX in their potencies toward KB tumor cells. For compounds **4**, **9**, and **10**, the IC_50_ values were improved from those for PMX by 32- to 46-fold (Table 1).

Collectively, these studies establish a selectivity of the 6-substituted thieno[2,3-*d*]pyrimidine antifolates for cellular uptake by FR-α and -β over the facilitative folate transporters RFC and PCFT.

#### Identification of Intracellular Targets for Thieno[2,3-*d*]pyrimidine Analogs by Metabolite Rescue

**Figure 7:**
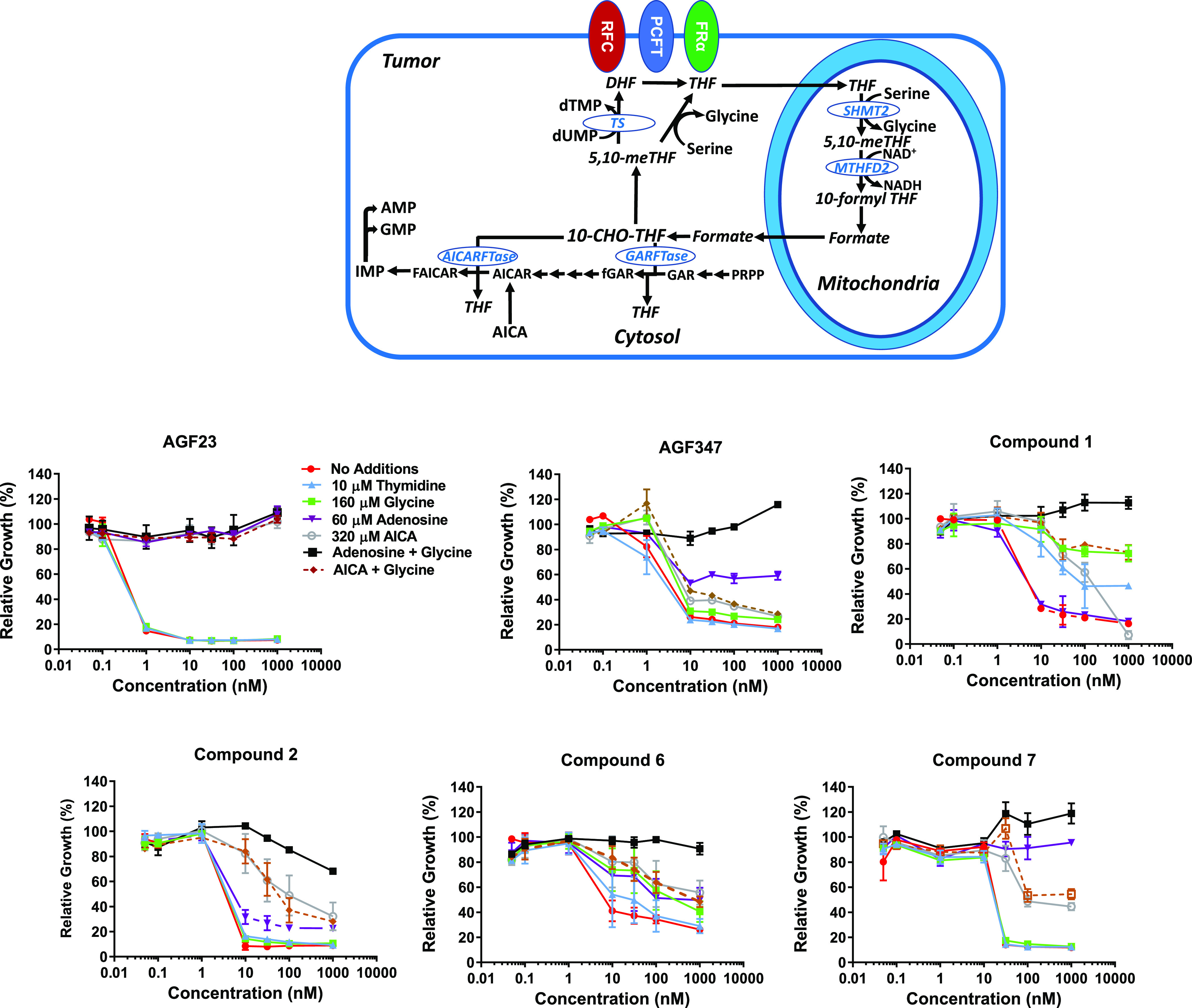
Growth inhibition of KB human tumor cells by thieno[2,3-*d*]pyrimidine analogs and the protective effects of nucleosides, glycine, and/or AICA compared to **AGF23** and **AGF347**. Upper panel: schematic of C1 metabolism. Undefined abbreviations include the following: DHF, dihydrofolate; THF, tetrahydrofolate; FAICAR, formyl 5-aminoimidazole-4-carboxamide ribonucleotide; fGAR, formyl GAR; and 5,10-meTHF, 5,10-methylene tetrahydrofolate. Lower panels: KB cells were plated (4000 cells/well) in glycine-, nucleoside-, and folate-free RPMI 1640 medium with 10% dialyzed fetal bovine serum, antibiotics, l-glutamine, and 2 nM leucovorin over a range of inhibitor concentrations in the presence of adenosine (60 μM), glycine (160 μM), thymidine (10 μM), or AICA (320 μM). As shown, combined glycine plus adenosine or AICA was also tested to identify potential mitochondrial C1 targeting.^45^ Cell proliferation was assayed with a fluorescence-based assay.^26^ Data are representative of at least triplicate experiments (presented as mean values plus/minus standard errors). Methods are described in the Experimental Section, and additional metabolite rescue data for **4**, **5**, and **8**–**11** are presented in Figure S3 (Supporting Information).

C1 metabolism involves folate-dependent pathways predominantly in the cytosol and mitochondria leading to the synthesis of purine nucleotides and thymidylate.^1,2^ Mitochondrial C1 metabolism converts serine to glycine and 10-formyl tetrahydrofolate (THF) (10-CHOTHF) (via serine hydroxymethyltransferase 2 (SHMT2) and 5,10-methylene THF dehydrogenase 2 (MTHFD2)). 10-CHOTHF is converted to formate (via 5,10-methylene THF dehydrogenase 1-like (MTHFD1L)), which in the cytosol (as 10-CHOTHF and 5,10-methylene THF) provides C1 units for cellular biosynthesis (Figure 7, upper panel).^1−3^

Metabolite “rescue” experiments have been used to identify targeted pathways for novel antifolate inhibitors including *de novo* purine nucleotide and thymidylate biosynthesis (protection by adenosine and thymidine, respectively)^25−27,29,30,32,43,45−47^ and mitochondrial C1 metabolism (protection by glycine and adenosine).^45^ To gain insights into the targeted pathways that contribute to the loss of KB tumor cell proliferation upon treatment with compounds **3**–**9**, we tested the reversal of growth inhibition by the active compounds in Table 1 (up to 1000 nM) in *glycine- and nucleoside-free* media in the presence of 60 μM adenosine, 160 μM glycine, or 10 μM thymidine alone or with combined adenosine and glycine. The use of glycine- and nucleoside-free media for these experiments expands the ability to identify causal intracellular targets (including previously unrecognized mitochondrial targets^45^) that can be confirmed by targeted metabolomics and direct *in vitro* assays (*below*). For the 6-substituted thieno[2,3-*d*]pyrimidine series of compounds, results were compared to those for **AGF23**, a 6-substituted pyrrolo[2,3-*d*]pyrimidine antifolate with a 3-carbon bridge and an established inhibitor of GARFTase,^26,32^ and for **AGF347**, a 5-substituted pyrrolo[3,2-*d*]pyrimidine inhibitor of SHMT2 in mitochondria, along with serine hydroxymethyltransferase 1 (SHMT1) and *de novo* purine biosynthesis (at GARFTase and AICARFTase) in the cytosol.^45^ We also tested compounds **1**, **2**, **10**, and **11**, established inhibitors of both GARFTase and AICARFTase from prior studies in glycine replete media.^25,32^ The results of these experiments are shown in Figure 7 (lower panel) and in Figure S3 (Supporting Information).

The effects of adenosine addition resulted in substantial differences between compounds of this series. For compounds **5**, **8**, **10**, and **11**, adenosine was completely protective, establishing *de novo* purine biosynthesis as the targeted pathway (Figure S3, Supporting Information). For compounds **4**, **7**, and **9**, adenosine by itself was substantially protective up to 1000 nM inhibitor, although rescue was slightly and reproducibly enhanced when glycine was combined with adenosine (Figure 7 and in Figure S3, Supporting information). This suggests a *modest* secondary inhibition of mitochondrial C1 metabolism.^45^

Interestingly, for compound **6**, as well as for compounds **1** and **2**, the increased protection by glycine plus adenosine over adenosine alone was substantially enhanced and was complete, results resembling those for **AGF347**(45) (Figure 7). These results strongly suggest a significant inhibition of mitochondrial C1 metabolism for compounds **1**, **2**, and **6**, along with direct or indirect effects on the *de novo* purine biosynthetic pathway.^45^

To explore the possibility that AICARFTase (as opposed to GARFTase alone) in *de novo* purine biosynthesis (Figure 7, upper) was a target for the thieno[2,3-*d*]pyrimidine compounds, we tested the protective effects of exogenous 5-aminoimidazole-4-carboxamide (AICA) (320 μM)^32^ without additions or AICA combined with glycine. AICA circumvents the first folate-dependent enzyme, GARFTase, and is directly metabolized to ZMP (AICAR), thus providing a substrate for AICARFTase^26,32,46^ (Figure 7, upper panel). This distinguishes primary inhibition at GARFTase (protection by AICA) versus AICARFTase (incomplete protection by AICA) for inhibitors of *de novo* purine biosynthesis.^25^

AICA alone completely protected KB cells from the inhibitory effects of the GARFTase inhibitor **AGF23** (Figure 7), confirming the selective inhibition of GARFTase over AICARFTase.^26,32^ However, for all of the thieno[2,3-*d*]pyrimidine compounds (**1**, **2**, **4**–**10**), AICA was only partly effective in reversing growth inhibitory effects (Figure 7 and Figure S3, Supporting Information), and this effect was completely independent of glycine addition. This suggests that at least part of the inhibitory effects of this series is due to direct targeting at AICARFTase, although additional inhibition of GARFTase is possible.

Collectively, the metabolite rescue results suggest that, in KB tumor cells, intracellular targets for the series of 6-substituted thieno[2,3-*d*]pyrimidine antifolates vary for different compounds and include AICARFTase and possibly GARFTase, combined with mitochondrial C1 metabolism for a subset of compounds (**1**, **2**, and **6**).

#### Identification of SHMT2 as a Target for 6-Substituted Thieno[2,3-*d*]pyrimidine Antifolates **1**, **2**, and **6** by Targeted Metabolomics

To confirm the inhibition of mitochondrial C1 targets for compounds **1**, **2**, and **6** suggested by the metabolite rescue experiments (Figure 7), we assayed mitochondrial C1 metabolism from serine using a [2,3,3-^2^H]serine tracer in KB tumor cells. Serine is catabolized in the mitochondria via SHMT2, which converts serine to glycine and 5,10-methylene THF, the latter of which is metabolized by MTHFD2 to 10-CHOTHF and by MTHFD1L to formate (Figure 7).

**Figure 8:**
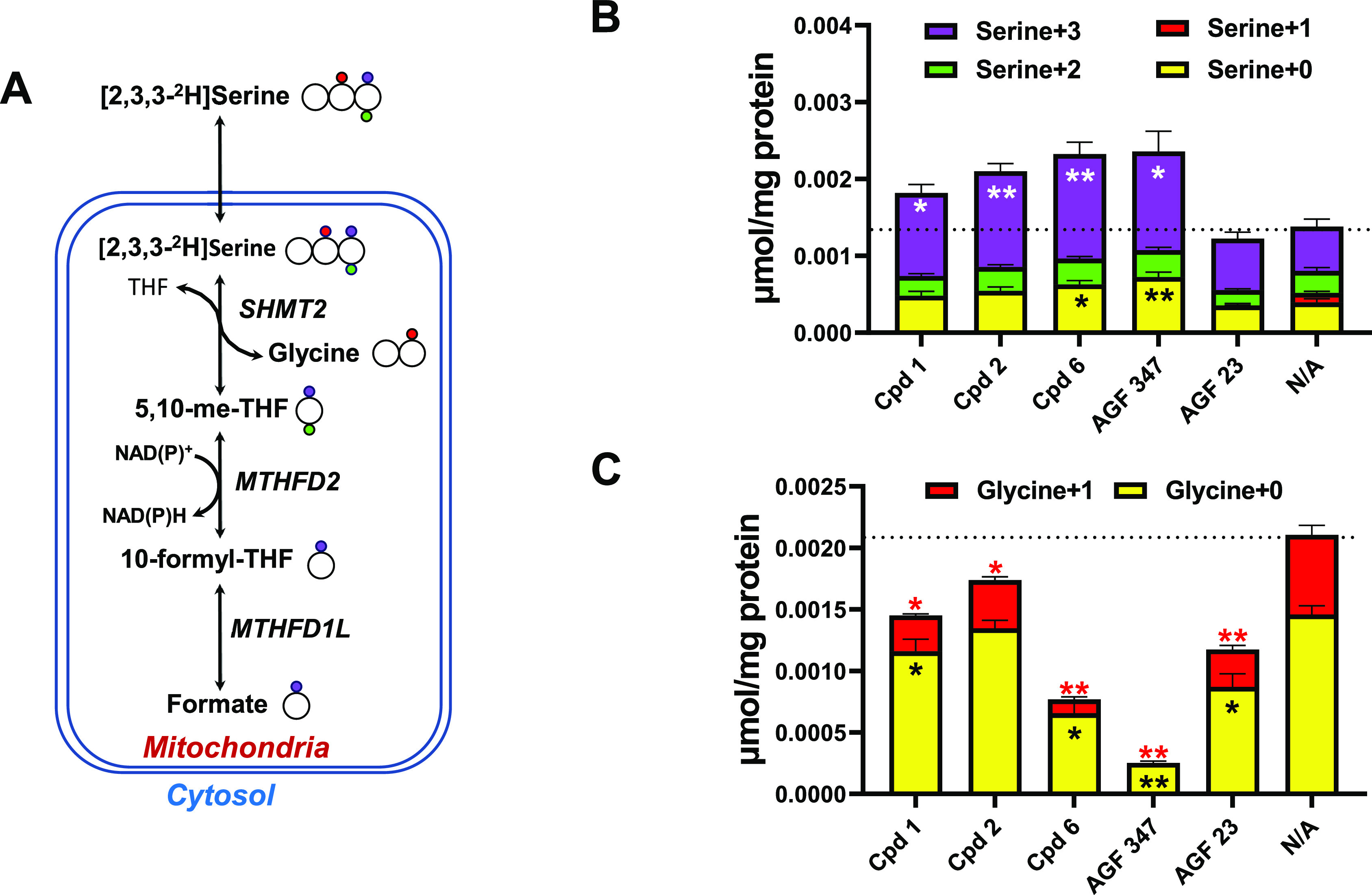
Metabolomics flux analysis with [2,3,3-^2^H]serine to assay mitochondrial C1 metabolism via SHMT2. (A) A schematic of [2,3,3-^2^H]serine metabolism is shown. Heavy (^2^H) atoms in serine (colored circles) are metabolized via SHMT2 initially followed by MTHFD2 and MTHFD1L to formate. (B, C) Total serine and glycine and the distribution of the serine (M + *n*, *n* = 0−3) and glycine (*n* = 0 or 1) isotopomers are shown. The loss of M + 3 serine corresponding to the metabolism of [2,3,3-^2^H]serine to M + 1 glycine is a direct measure of SHMT2 activity in the cells.^45^ Experimental methods are described in the Experimental Section. Results are shown as mean values plus/minus standard deviations (*n* = 3). *P < 0.05; **P < 0.01. Statistical comparisons were between vehicle-treated and inhibitor-treated cells. N/A, no additions.

KB cells were incubated (48 h) with [2,3,3-^2^H]serine (250 μM) with compounds **1**, **2**, or **6** (0.1 μM); with the GARFTase inhibitor **AGF23** (negative control); or with the SHMT2 inhibitor **AGF347** (positive control). The cells were processed for liquid chromatography–mass spectrometry (LC–MS) analysis of total serine and glycine, and deuterated serine (M + 3, M + 2, M + 1, and M + 0 serine, where M + *n* represents species with *n* deuterium atoms) and glycine (M + 1 and M + 0) isotopomers. The results are shown in Figure 8 and are compared to those for control (vehicle-treated) cells (labeled “N/A”). Compounds **1**, **2**, and **6** effected a statistically significant 2–4-fold increase in M + 3 serine accompanied by a 1.5- to 6-fold decrease in M + 1 glycine, results resembling those for **AGF347** and consistent with direct targeting and inhibition of SHMT2 in the mitochondria. As expected, changes in M + 3 serine were nominal and insignificant for **AGF23**, although M + 1 glycine was suppressed.

#### *In Vitro* Validation of Intracellular Targets with Isolated C1 Metabolic Enzymes

From the results of the metabolite rescue experiments in KB tumor cells, important cellular targets for the thieno[2,3-*d*]pyrimidine inhibitors were identified including AICARFTase and GARFTase in *de novo* purine biosynthesis (Figure 7). For compounds **1**, **2**, and **6**, direct targeting of SHMT2 was strongly suggested by glycine rescue and was further indicated by metabolomic flux analysis with [2,3,3-^2^H]serine analogous to **AGF347**(45) (Figure 8).

We directly tested inhibition potencies by *in vitro* assays using purified GARFTase and ATIC (AICARFTase) to calculate inhibition dissociation constants (*K*_i_’s) (Table 2).^45^ Results for compounds **3**–**9** were compared to those for previously published compounds **1**, **2**, **10**, and **11**.^32^ In general, the compounds inhibited one or both enzymes at nanomolar to low micromolar potencies. The *K*_i_’s for GARFTase spanned a ∼110-fold range with potencies in rank order of **9** > **2** ∼ **8** ∼ **10** ∼ PMX > **5** > **1** > **3**. No inhibition of GARFTase was detected for compounds **4**, **6**, **7**, **11**, or MTX up to 150 μM. For ATIC, the rank potencies were PMX > **6** ∼ **11** > **2** > **4** > **8** > **3** > **7** ∼ **10** > **9**. Neither compounds **5** and **1** nor MTX inhibited ATIC up to 200 μM.

**Table 2:** *K*_i_ Values for the Inhibition of Human GARFTase and ATIC by 6-Substituted Thieno[2,3-*d*]pyrimidine Compounds^a^

		GARFTase	ATIC
compound		*K*_i_ (μM)	*K*_i_ (μM)
**1**	3C/1′4′phenyl	18.5 (7.3)	>200
**2**	4C/1′4′phenyl	3.0 (0.9)	9.5 (2.8)
**6**	3C/2′F/1′4′phenyl	>150	7.3 (3.2)
**7**	4C/2′F/1′4′phenyl	>150	19.4 (5.7)
**3**	4C/2′6′pyridine	26.7 (6.8)	18.4 (0.8)
**4**	4C/2′5′pyridine	>150	12.1 (1.8)
**5**	4C/3′F/2′5′pyridine	13.7 (5.2)	>200
**10**	3C/2′4′thiophene	4.3 (1.1)	19.8 (5.3)
**11**	4C/2′4′thiophene	>150	8.0 (2.7)
**8**	3C/3′F/2′4′thiophene	3.1 (1.2)	14.5 (3.4)
**9**	4C/3'F/2′4′thiophene	0.17 (0.06)	23.7 (11.2)
MTX		>150	>200
PMX		5.2 (1.6)	0.88 (0.56)

a *K*_i_ values for the inhibition of GARFTase and ATIC by the monoglutamyl antifolates are shown. Results are shown as mean values ± standard errors from at least three replicate assays in parentheses. The results for **2**, **10**, **11**, MTX, and PMX were previously reported.^32,45,48^ Detailed methods are described in the Experimental Section.

Interestingly, the impact of the aromatic side chain and fluorine substitutions on GARFTase inhibition varied for the phenyl, pyridine, and thiophene compounds, in part depending on the length of the alkyl bridge (3- versus 4-carbons) (Table 2). Whereas compounds **6** and **7** with a 2′-F-phenyl side chain were inactive up to 150 μM, the 3′F thiophene compounds **8** and **9** were among the most potent GARFTase inhibitors of the series with the 4-carbon bridge (**9**) predominating over the 3-carbon bridge (**8**). Compound **9** showed a *K*_i_ of 0.17 μM (17-, 25-, and 882-fold more potent than **2**, **10**, and **11,** respectively). For ATIC, with the exception of compound **5**, all thieno[2,3-*d*]pyrimidine compounds showed similar inhibition potencies within a ∼3-fold range (Table 2).

On the basis of the results of our metabolomics experiments (Figure 8), we also measured the inhibition of the mitochondrial target SHMT2 by compounds **1, 2**, and **6** compared to **AGF347**.^45^ Whereas **1**, **2**, and **6** all inhibited SHMT2, this was substantially less than that for **AGF347** (Table 3).

**Table 3:** *K*_i_ Values for the Inhibition of SHMT2 by 6-Substituted Thieno[2,3-*d*]pyrimidine Compounds **1**, **2**, and **6**^a^

compound		SHMT2 *K*_i_ (μM)
**AGF347**	4C/2′F/1′4′phenyl	0.45 (0.19)
**1**	3C/1′4′phenyl	67.3 (28)
**2**	4C/1′4′phenyl	12.4 (3)
**6**	3C/2′F/1′4′phenyl	27.6 (13)

a Results are shown for *K*_i_’s for SHMT2 as mean values ± standard errors from at least three replicate assays in parentheses. Detailed methods are in the Experimental Section.

Thus, in general, the results of the *in vitro* assays with the isolated enzymes validate the cell-based inhibition data that indicate GARFTase and AICARFTase targeting by the thieno[2,3-*d*]pyrimidine antifolates. Our findings with compounds **1**, **2**, and **6** represent the first reported discovery of thieno[2,3-*d*]pyrimidine scaffolds showing mitochondrial SHMT2 inhibitory activity.

#### X-ray Crystal Structures of 6-Substituted Thieno[2,3-*d*]pyrimidine Antifolates with Human GARFTase

**Figure 9:**
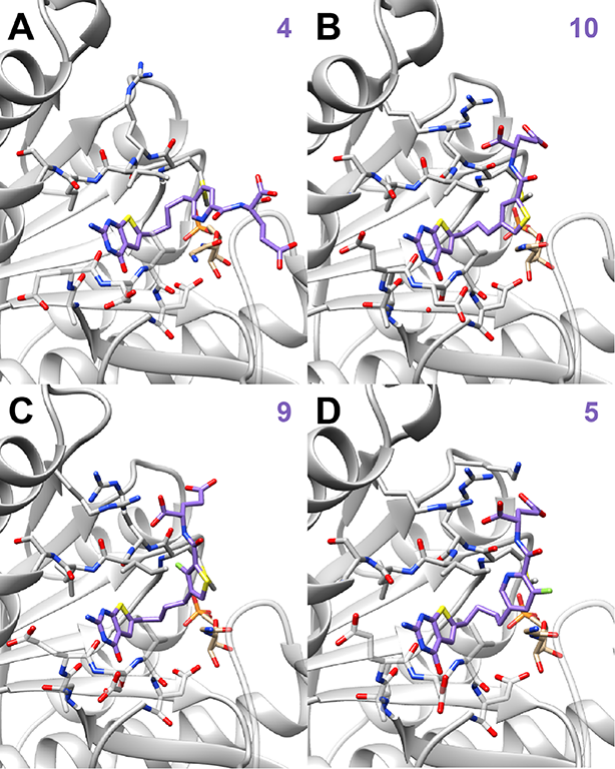
Structural analyses of 6-substituted thieno[2,3-*d*]pyrimidine analogs in GARFTase-β-GAR-antifolate ternary complexes. (A) Crystal structure of compound **4** (purple) bound in the 10-CHOTHF binding pocket of GARFTase with the natural substrate β-GAR (tan) and interacting residues shown as sticks. Structures of **10** (B), **9** (C), and **5** (D) are shown in a similar fashion as the structure for **4** (A). Data processing and refinement statistics (Table S2), as well as detailed interactions and maps for the complexes (Figures S4–S7), are included in the Supporting Information.

Our *in vitro* results demonstrate that compound **9** is a uniquely potent inhibitor of human GARFTase (Table 2). To better understand the molecular effects of modifications to 6-substituted thieno[2,3-*d*]pyrimidine analogs on GARFTase inhibition, we determined the structures of select analogs (**4**, **5**, **9**, and **10**) with a range of inhibitor potencies (Table 2) in complex with GARFTase and β-GAR substrate. The thieno[2,3-*d*]pyrimidine scaffold of all analogs makes polar contacts with the backbone atoms of Leu899, Glu948, and Asp951 in GARFTase (Figures S4–S7, Supporting Information). The most potent analog, **9** (0.17 μM), is a fluorinated thieno compound with the fluorine positioned to make an intramolecular contact with the amide hydrogen of the l-glutamate moiety (as predicted from NMR and molecular docking), thus restricting the side chain. The α-carboxylate of the l-glutamate of **9** is stabilized in the GARFTase active site by contacts with the side chain of Arg871, as well as the backbone amide of Ile898 (Figure S6, Supporting Information). Compound **9** is 882-fold more inhibitory toward GARFTase than the corresponding des-fluoro **11**, indicating the importance of the fluorine for GARFTase inhibition in this structural motif. Compound **10**, a nonfluorinated 3-C bridged thieno compound, is ∼25-fold less active than **9** in GARFTase inhibition and exhibits the same contacts between the l-glutamate and GARFTase as those seen for **9**. This implies that fluorination in **9** relative to the closely related nonfluorinated compounds **10** and **11** may bias the conformation prior to binding GARFTase, thereby reducing its entropic penalty upon binding (i.e., leading to increased potency). Compound **4**, an analog that does not inhibit GARFTase, makes no contacts with these residues and instead makes a contact between the amide NH of the l-glutamate and the hydroxyl of Ser925, orienting the linker pyridine and glutamyl tail away from Arg971 and Arg897 and toward residues 916–927 (Figure S4, Supporting Information). In compound **4**, the pyridine nitrogen is oriented syn to the amide carbonyl group and away from the amide NH, thus precluding any conformational restriction and perhaps contributing to its lack of GARFTase activity. Compound **5**, a fluorinated analog of the noninhibitor **4**, shows modest inhibition with a *K*_i_ = 13.7 μM, implying that intramolecular conformational stabilization occurs with the pyridine N···H–N that positions **5** to bind to GARFTase. Compound **5** harbors the same polar contacts between GARFTase and its l-glutamate as **9** and **10**, and the pyridine N of **5** now forms the intramolecular contacts with the amide NH, as indicated in the crystal structure (Figure 9).

Taken together, our comparative analysis of the crystal structures of the fluorinated compounds **9** and **5** versus related des-fluorinated compounds **4** and **10** provides molecular details in the solid state, which account for the dramatic increase in potency in one case where an intramolecular fluoro-hydrogen contact may be important and another in which an intramolecular pyridine N-hydrogen contact with the amide NH is necessary. Both intramolecular bonds in **9** and **5** restrict the conformation and are clearly important for GARFTase inhibition.

## Conclusions

**Figure 10:**
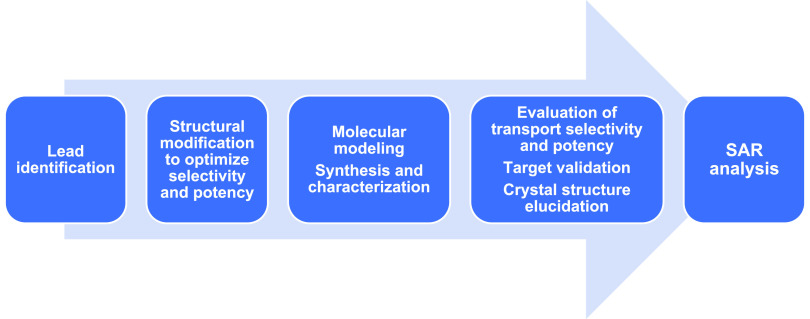
Workflow of the experiments.

The workflow for our studies is depicted in Figure 10. The goal was to further develop the structure–activity profiles for 6-substituted thieno[2,3-*d*]pyrimidine antifolates by selectively targeting tumors via FRs and inhibition of C1 metabolism. We previously discovered that in contrast to 5- and 6-substituted pyrrolo[2,3-*d*]pyrimidine antifolates,^26−30,43,46,47^ 6-substituted thieno[2,3-*d*]pyrimidine benzoyl antifolates were selective for FRs over the facilitative transporters RFC and PCFT.^25,32^ Following internalization by FRs, antitumor efficacy was attributed to the inhibition of *de novo* purine biosynthesis at AICARFTase, the second folate-dependent reaction, in addition to GARFTase.^25^ Subsequent studies established that replacement of the side-chain phenyl with a 2′4′-thienyl ring increased inhibitory potency.^32^

In the present study, we explored the impact of strategically placed nitrogen or fluorine atoms in the aromatic side chain (i.e., 1′,4′-phenyl, 2′4′-thienyl, and 2′5′-pyridine) of the thieno[2,3-*d*]pyrimidine series of compounds on transporter specificities and inhibition of intracellular enzyme targets. We used a robust readout of transporter specificities involving an engineered panel of isogenic CHO cell lines individually expressing human RFC, PCFT, FRα, or FRβ and extended studies to include KB human tumor cells that express RFC, PCFT, and FRα. Thus, replacement of the side-chain 1′,4′-phenyl ring with a 2′,5′-pyridyl ring or a fluorinated 2′,5′- pyridyl with the fluorine insertion ortho to the l-glutamate resulted in increased potencies of the thieno[2,3-*d*]pyrimidine antifolates toward FR-expressing CHO cells, with these analogs also potently inhibiting proliferation of KB human tumor cells. 2′*F*-Phenyl insertions (compounds **6** and **7**) resulted in increased potencies over the des-fluoro compounds **1** and **2** toward FR-expressing CHO cells that extended to the 3′F,2′4′-thiophene compound **9** and the 3′F,2′5′-pyridine compound **5**. Interestingly, for several series, these effects on FR-targeted cell inhibitions were more pronounced for FRβ and were independent of the nature of the aromatic side chain. This suggests a potential utility of these compounds for selectively targeting FRβ-expressing cells including protumor TAMs in the tumor microenvironment.^16,49^

By metabolite rescue studies, we identified *de novo* purine biosynthesis as the targeted pathway for the thieno[2,3-*d*]pyrimidine antifolates and discovered that compounds **1**, **2**, and **6** also inhibited mitochondrial C1 metabolism. By *in vitro* assays with monoglutamyl thieno[2,3-*d*]pyrimidine antifolates and isolated GARFTase and AICARFTase, inhibition of these folate-dependent targets in *de novo* purine biosynthesis was confirmed. Compound **9** with a side-chain 3′F,2′,4′ thiophene was the most potent GARFTase inhibitor we discovered with a potency 17- to 882-fold greater than the previous lead analogs of this series. For compounds **1**, **2**, and **6**, inhibition of mitochondrial C1 metabolism at SHMT2 was confirmed by metabolomic flux analysis with [2,3,3-^2^H]serine and direct assays of SHMT2 inhibition with isolated recombinant SHMT2. Mitochondrial SHMT2 inhibition by compounds **1**, **2**, and **6** would exacerbate the impact of directly inhibiting enzymes in *de novo* purine biosynthesis in the cytosol by limiting the source of C1 units for cytosolic anabolism.^1,2^

Fluorinated agents exemplified by compounds **5**–**9** offer structural simplicity with improved biological effects and represent an important step toward further optimizing tumor-targeted thieno[2,3-*d*]pyrimidines with selective transport via FRs over RFC. Molecular modeling studies indicated that the pyridine analog and the fluorinated pyridine analogs adopted bound conformations in FRα and FRβ and GARFTase and AICARFTase where the nitrogen in the pyridine ring is positioned on the same side (syn) as the NH of the l-glutamate. This is due to the electronic interaction with the binding pocket, as well as the ability to stabilize the side-chain binding conformation through intramolecular hydrogenbonding. The presence of a fluoro-amide hydrogen contact and a pyridine-amide NH contact was corroborated by NMR and was also seen in the crystal structure of GARFTase in complex with compounds **9** and **5**, respectively.

Of course, although molecular modeling and studies of isolated enzyme inhibition potencies establish structure–activity relationships for the thieno[2,3-*d*]pyrimidine antifolates, these results only partly reflect those for the *in vitro* cell-based efficacy studies (Table 1) as they neither account for the impact of differences in membrane transport or polyglutamate synthesis^5^ nor account for the role of multienzyme associations (“purinosome”), which are important to the efficient synthesis of purine nucleotides in cells.^50^

In conclusion, our results document novel first-in-class thieno[2,3-*d*]pyrimidine inhibitors with FR selectivity, including increased potency toward FR-expressing cells with newly discovered fluorine-substituted analogs, and inhibition of metabolic pathways that are essential to malignant cells including *de novo* purine nucleotide biosynthesis and, for certain compounds, mitochondrial C1 metabolism.^1,2,51^ Multitargeted inhibitors such as those described herein offer substantial advantages in circumventing resistance to single-target drugs. Clearly, these multitargeted thieno[2,3-*d*]pyrimidine agents represent an exciting new structural platform for targeted cancer therapy with substantial advantages of selectivity and potency over clinically used antifolates.

## Methods

### General Information

Chemicals and solvents were purchased from the following companies, including Oakwood Chemical, Matrix Scientific, Ark Pharm. Inc., Aldrich Chemical Co., or Fisher Scientific Co. For ^1^H NMR spectra, a Bruker WH-400 MHz or AV-III 500 MHz spectrometer equipped with a BBFO-Plus probe was used. In the NMR analysis study, the synthesized molecules were prepared in CDCl_3_ or DMSO-*d*_6_ as the NMR solvent. Chemical shift values are reported in ppm (parts per million) relative to tetramethylsilane (internal standard). Singlets, doublets, triplets, quartets, multiplet, and broad singlets are abbreviated as s, d, t, q, m, and br, respectively. The relative integrals of peak areas match with the assigned structures, and chemical names adhere to the IUPAC nomenclature. Analytical thin-layer chromatography (TLC) analysis was conducted on silica gel plates (Whatman Sil G/UV254) with a fluorescent indicator, and the spots were visualized under a UV lamp at the wavelength of 254 and 365 nm. Silica gel (230–400 mesh; Thermo-Fisher Scientific) column chromatography was used to purify the compounds. A rotary evaporator and vacuum pump were utilized for the evaporation process *in vacuo*. The final compounds were dried overnight using a CHEM-DRY apparatus over P_2_O_5_ at 50 °C. For some of the final compounds, even after 24 to 48 h of drying *in vacuo*, solvents could not be entirely removed, and the fractional moles were confirmed by their presence in their respective ^1^H NMR spectra. Melting points of the solids were recorded on an uncorrected FLUKE 51 K/J electronic thermometer equipped MEL-TEMP II melting point apparatus. High-resolution mass spectrometry (HRMS) data were acquired using the ESI probe on a Thermo Scientific LTQ Orbitrap XL system. Reverse-phase HPLC was performed on a ThermoFisher UltiMate 3000 UHPLC Systems using an XSelect HSS T3 XP C18 Column, 100 Å, 2.5 μm, 3 × 100 mm, from Waters company and a Luna 5 μm C18(2) 100 Å, LC Column 250 × 4.6 mm, from Phenomenex for the determination of purity or structure identification. Compounds **3**–**9** are >95% pure by HPLC analysis.

### Molecular Modeling and Computational Studies

The potential binding interactions, binding energies, and binding modes were analyzed by docking the designed analogs into the X-ray crystal structures of human FRα (5IZQ),^27^ FRβ (4KN2,)^37^ GARFTase (7JG0),^32^ and ATIC (1P4R),^38^ respectively. The crystal structures were obtained from the protein database RCSB and subjected to standard preparation and optimization using the standard Maestro protein preparation wizard to evaluate bond order and missing hydrogens. An energy minimization mode was then performed using the standard OPLS3e force field. The Maestro LigPrep module was used to prepare and generate the energy minimized conformations of the three-dimensional (3D) geometries of ligands using the OPLS3e force field protocol. Using the Maestro induced-fit module, the energy minimized ligands were docked within a workspace box similar to the co-crystallized ligands around the crystallographic binding poses. The extended precision mode was utilized to perform redocking. Redocking of each ligand was carried out in its corresponding low-energy protein structures, and the resulting complexes generated were ranked according to the docking scores. For each compound, the 20 top-scoring binding modes were generated and visually analyzed. The best pose (ligand–receptor complex) was selected based on the docked scores with the lowest energies (in kcal/mol) for further analysis of the binding interactions. Modeling figures were generated with the Chimera visualization software.^52^

### Synthesis of Compounds

#### General Procedure to Prepare Intermediates **13a**–**c**

To a round-bottom flask equipped with a magnetic stirrer, commercially available bromo-(het)arylcarboxylic acids (**12a**–**c**) (1 equiv), *N*-methyl morpholine (1.2 equiv), and 2-chloro-4,6-dimethoxy-1,3,5-triazine (1.2 equiv) in anhydrous DMF were added. The reaction mixture was stirred for 2 h at room temperature, and *N*-methyl morpholine (1.2 equiv) and l-glutamic acid dimethyl ester hydrochloride (1.5 equiv) were added to the mixture. The reaction was stopped after an additional 10 h stirring. Upon completion of the reaction, the solvent DMF was removed under a vacuum followed by addition of MeOH and silica gel. The resulting plug was subjected to column chromatography on the silica gel using gradient elution with hexanes and gradually increasing amounts of ethyl acetate, up to 10% ethyl acetate in hexanes. Fractions with the desired *R*_f_ (TLC) were pooled, and the solvent was removed under reduced pressure to yield glutamate esters **13a**–**c** as light-yellow liquids.

##### Dimethyl (6-Bromopicolinoyl)-l-glutamate (**13a**)

The synthesis of compound **13a** was carried out following the same general method used for the preparation of **13a**–**c**, from 6-bromopicolinic acid, **12a** (2 g, 9 mmol), to afford 2.45 g of **13a** as a light-yellow liquid in 67% yield. TLC *R*_f_ = 0.45 (hexane/EtOAc, 1:1). ^1^H NMR (400 MHz, CDCl_3_): δ 2.04–2.25 (m, 2H, CH_2_), 2.40 (t, *J* = 7.3 Hz, 2H, CH_2_), 3.57 (s, 3H, CH_3_), 3.66 (s, 3H, CH_3_), 4.56 (ddd, *J* = 9.2, 8.0, 5.0 Hz, 1 H, Gluα-CH), 7.90 (dd, *J* = 7.9, 1.1 Hz, 1H, Ar.), 7.93–7.99 (m, 1H, Ar.), 8.04 (dd, *J* = 7.5, 1.0 Hz, 1H, Ar.), 8.96 (d, *J* = 8.1 Hz, 1H, CO-NH, exch.).

##### Dimethyl (5-Bromo-3-fluoropicolinoyl)-l-glutamate (**13b**)

The synthesis of compound **13b** was carried out following the same general method used for the preparation of **13a**–**c**, from 5-bromo-3-fluoropicolinic acid, **12b** (2 g, 9 mmol), to afford 2.5 g of **13b** as a light-yellow liquid in 69% yield. TLC *R*_f_ = 0.45 (hexane/EtOAc, 1:1). ^1^H NMR (400 MHz, CDCl_3_): δ 1.97–2.19 (m, 2H, CH_2_), 2.42 (t, *J* = 7.6 Hz, 2H, CH_2_), 3.58 (s, 3H, CH_3_), 3.66 (s, 3H, CH_3_), 4.77–4.55 (m, 1H, Gluα-CH), 8.39 (dd, *J* = 10.1, 1.8 Hz, 1H, Ar.), 8.66–8.72 (m, 1H, Ar.), 9.06 (d, *J* = 8.0 Hz, 1H, CONH, exch.).

##### Diethyl (4-Bromo-3-fluorothiophene-2-carbonyl)-l*-*glutamate (**13c**)

The synthesis of compound **13c** was carried out following the same general method used for the preparation of **13a**–**c**, from 4-bromo-3-fluorothiophene-2-carboxylic acid, **12c** (2 g, 8.9 mmol), to afford 1.64 g of **13c** as a light-yellow liquid in 45% yield. TLC *R*_f_ = 0.50 (hexane/EtOAc, 1:1). ^1^H NMR (400 MHz, CDCl_3_): δ 1.30 (dt, *J* = 26.5, 7.2 Hz, 6H, 2CH_3_), 3.73–3.77 (m, 2H, CH_2_), 3.85–3.89 (m, 2H, CH_2_), 4.15 (q, *J* = 7.2 Hz, 2H, CH_2_), 4.27 (qd, *J* = 7.1, 2.0 Hz, 2H, CH_2_), 4.77–4.85 (m, 1H, Gluα-CH), 7.05 (q, *J* = 7.7 Hz, 1 H, CO-NH), 7.43 (d, *J* = 4.0 Hz, 1H, Ar.).

##### 2-Amino-6-(but-3-yn-1-yl)thieno[2,3-*d*]pyrimidin-4(*3H*)-one (**14**)

Key intermediate **14** was synthesized from the commercially available 5-hexyn-1-ol by oxidizing the alcohol using Dess–Martin periodinane (DMP) in DCM. After 3 h, the reaction mixture was gradually warmed from 0 °C to room temperature in an ice bath and then treated with dropwise addition of 10 mL 1 N NaOH solution to deactivate the residual DMP. Ethyl acetate was then used to extract the resulting solution three times, with each extraction using 30 mL of solvent. The organic solution obtained after extraction was combined and evaporated to dryness, and then a microwave-assisted Gewald reaction was carried out in the presence of the aldehyde with a mixture of sulfur, ethyl cyanoacetate, ethanol, and triethylamine in a sealed microwave vial irradiated in the microwave reactor at 80 °C for 30 min. Subsequently, the mixture of the appropriate thiophene (1 equiv) obtained by the Gewald reaction was mixed with chloroformamidine hydrochloride (4 equiv) in dimethyl sulfone (DMSO_2_), and the mixture was then heated at 140 °C for 4 h. After the completion of the reaction, 20 mL of water was added before the mixture was cooled to room temperature, after which ammonium hydroxide was added dropwise to neutralize the suspension to pH over 7. After filtration, the resulting brown solid was washed with water and then dried over P_2_O_5_ to afford the key intermediate **14** in 68% yield. mp > 250 °C. TLC *R*_f_ = 0.46 (CHCl_3_/MeOH, 8:1). ^1^H NMR (400 MHz, DMSO-*d*_6_): δ 2.44–2.49 (m, 2H, CH_2_), 2.83–2.89 (m, 3H, CH_2_ and CH), 6.50 (s, 2H, 2-NH_2_, exch.), 6.89 (s, 1H, Ar.), 10.89 (s, 1H, 3-NH, exch.). LRMS (ESI) calculated for chemical formula C_10_H_9_N_3_OS, 219.05, found: 218.9.

#### General Procedure to Prepare Intermediates **15a**–**c**

The catalyst tetrakis(triphenylphosphine)palladium(0) (0.16 equiv), triethylamine (10 equiv), and **13a**–**c** (1.5 equiv) were added to 10 mL of anhydrous DMF in a N_2_-purged microwave vial equipped with a stir bar. While stirring under N_2_, copper(I) iodide (0.16 equiv) and **14** (1 equiv) were added, and the reaction mixture was subsequently reacted in a microwave reactor at 70 °C for 12 h. Upon completion of the reaction, DMF was removed under a vacuum followed by addition of MeOH and silica gel, and the solvent was further evaporated under reduced pressure. The resulting plug was subjected to column chromatography on silica using gradient elution with CHCl_3_ followed by gradually increasing amounts of methanol up to 10% MeOH in CHCl_3_. Fractions with the desired *R*_f_ (TLC) were pooled, and the solvent was removed under reduced pressure to yield the alkyne intermediates **15a**–**c**.

##### Dimethyl (6-(4-(2-Amino-4-oxo-3,4-dihydrothieno[2,3-*d*]pyrimidin-6-yl)but-1-yn-1-yl)picolinoyl)-l-glutamate (**15a**)

The synthesis of intermediate **15a** was carried out following the same general method used for the preparation of **15a**–**c**, from **14** (100 mg, 0.46 mmol) and **13a** (258 mg, 0.74 mmol), to give 110 mg (44%) of **15a** as a brown sticky solid. TLC *R*_f_ = 0.5 (CHCl_3_/MeOH, 5:1); ^1^H NMR (400 MHz, DMSO-*d*_6_): δ 2.39 (t, *J* = 7.2 Hz, 2H, CH_2_), 2.83 (t, *J* = 7.1 Hz, 2H, CH_2_), 3.03 (t, *J* = 6.9 Hz, 2H, CH_2_), 3.10 (d, *J* = 2.4 Hz, 2H, CH_2_), 3.56 (d, *J* = 1.1 Hz, 3H, CH_3_), 3.66 (d, *J* = 1.1 Hz, 3H, CH_3_), 4.57 (m, 1H, Gluα-CH), 6.51 (s, 2H, 2-NH_2_, exch.), 6.99 (s, 1H, Ar.), 7.66 (dd, *J* = 7.1, 1.7 Hz, 1H, Ar.), 8.03–7.94 (m, 2H, Ar.), 8.90 (d, *J* = 8.3 Hz, 1H, CO-NH, exch.), 10.88 (s, 1H, 3-NH, exch.).

##### Dimethyl (5-(4-(2-Amino-4-oxo-3,4-dihydrothieno[2,3-*d*]pyrimidin-6-yl)but-1-yn-1-yl)-3-fluoropicolinoyl)-l-glutamate (**15b**)

The synthesis of compound **15b** was carried out following the same general method used for the preparation of **15a**–**c**, from **14** (100 mg, 0.46 mmol) and **13b** (258 mg, 0.74 mmol), to give 124 mg (45%) of **15b** as a brown sticky solid. TLC *R*_f_ = 0.45 (CHCl_3_/MeOH, 5:1); ^1^H NMR (400 MHz, DMSO-*d*_6_): δ 2.42 (t, *J* = 7.4 Hz, 2H, CH_2_), 2.84 (t, *J* = 6.9 Hz, 2H, CH_2_), 3.03 (t, *J* = 7.0 Hz, 2H, CH_2_), 3.10 (d, *J* = 7.4 Hz, 2H, CH_2_), 3.58 (s, 3H, CH_3_), 3.66 (s, 3H, CH_3_), 4.57 (m, 1H, Gluα-CH), 6.51 (s, 2H, 2-NH_2_, exch.), 6.99 (s, 1H, Ar.), 7.66 (dd, *J* = 7.1, 1.7 Hz, 1H, Ar.), 7.93–8.03 (m, 2H, Ar.), 8.90 (d, *J* = 8.3 Hz, 1H, CO-NH, exch.), 10.88 (s, 1H, 3-NH, exch.).

##### Dimethyl (4-(4-(2-Amino-4-oxo-3,4-dihydrothieno[2,3-*d*]pyrimidin-6-yl)but-1-yn-1-yl)-3-fluorothiophene-2-carbonyl)-l-glutamate (**15c**)

The synthesis of intermediate **15c** was carried out using the same general method described for the preparation of **15a**–**c**, from **14** (100 mg, 0.46 mmol) and **13c** (260 mg, 1.37 mmol), to give 112 mg (46%) of **15c** as a brown sticky semisolid. TLC *R*_f_ = 0.48 (CHCl_3_/MeOH, 5:1); ^1^H NMR (400 MHz, DMSO-*d*_6_): δ 1.97–2.18 (m, 2H, CH_2_), 2.43 (t, *J* = 7.4 Hz, 2H, CH_2_), 2.78 (t, *J* = 7.0 Hz, 2H, CH_2_), 2.96–3.01 (m, 2H, CH_2_), 3.59 (s, 3H, CH_3_), 3.66 (s, 3H, CH_3_), 4.40–4.57 (m, 1H, Gluα-CH), 6.50 (s, 2H, 2-NH_2_, exch.), 6.94 (s, 1H, Ar.), 7.96 (d, *J* = 4.3 Hz, 1H, Ar.), 8.40 (d, *J* = 8.1 Hz, 1H, CO-NH, exch.), 10.87 (s, 1H, 3-NH, exch.).

#### General Procedure for the Synthesis of Compounds **3**, **5**, and **9**

The solution of the compounds **15a**–**c** in methanol was introduced to a Parr flask that contained 10% palladium on activated carbon (200 mg) soaked in methanol. The hydrogenation process was conducted at 55 psi for approximately 24 h. After 24 h, the reaction mixture was filtered through Celite powder followed by multiple washes by MeOH and then concentrated under reduced pressure to yield the semisolid reduced alkanes. To the semisolids, two drops of 1 N NaOH solution were added, and the resulting mixture was stirred under N_2_ at room temperature for 3 h until the disappearance of the starting material (*R*_f_ = 0.45) and observation of one major spot at the origin (CHCl_3_/MeOH, 5:1) as confirmed by TLC analysis. After cooling the solution in an ice bath, the pH was adjusted to 3–4 by gradual addition of 1 N HCl. The resulting suspension was further cooled in an ice bath for an additional 30 min. The suspension was filtered, and the residue was washed thoroughly with cold water and dried under vacuum using P_2_O_5_ to afford the target compounds **3**, **5**, and **9**.

##### (6-(4-(2-Amino-4-oxo-3,4-dihydrothieno[2,3-*d*]pyrimidin-6-yl)butyl)picolinoyl)-l-glutamic Acid **3**

Final compound **3** was prepared using the same general method reported for the preparation of **3**, **5**, and **9** from **15a** (320 mg, 0.64 mmol) to give 150 mg (49% over 2 steps) of **3** as a light-yellow powder. mp: 125.8–126.2 °C. ^1^H NMR (400 MHz, DMSO-*d*_6_): δ 1.67 (q, *J* = 7.5 Hz, 2H, CH_2_), 1.78 (q, *J* = 7.5 Hz, 2H, CH_2_), 1.99–2.19 (m, 2H, CH_2_), 2.30 (t, *J* = 7.3 Hz, 2H, CH_2_), 2.77 (d, *J* = 7.4 Hz, 2H, CH_2_), 2.86 (t, *J* = 7.6 Hz, 2H, CH_2_), 4.49 (m, 1H, Gluα-CH), 6.47 (s, 2H, 2-NH_2_, exch), 6.82 (s, 1H, Ar.), 7.50 (d, *J* = 7.4 Hz, 1H, Ar.), 7.94–7.83 (m, 2H, Ar.), 8.73 (d, *J* = 8.1 Hz, 1H, CONH, exch.), 10.85 (s, 1H, NH, exch.). HRMS (ESI) calculated for C_21_H_24_N_5_O_6_S [M + H]^+^: 474.1442, found: 474.1439. HPLC analysis: retention time, 12.6 min; purity: 97.6%; eluent A, 0.1% formic acid in H_2_O: eluent B, 0.1% formic acid in ACN; gradient elution (5% ACN to 95% ACN) over 30 min with a flow rate of 0.5 mL/min and detected at 330 nm; column temperature, 25 °C.

##### (5-(4-(2-Amino-4-oxo-3,4-dihydrothieno[2,3-*d*]pyrimidin-6-yl)butyl)-3-fluoropicolinoyl)-l-glutamic Acid **5**

Final compound **5** was prepared using the same general method reported for the preparation of **3**, **5**, and **9** from **15b** (100 mg, 0.45 mmol) to give 110 mg (54% over 2 steps) of **5** as a light-yellow powder. mp: 128.0–129.7 °C. ^1^H NMR (400 MHz, DMSO-*d*_6_): δ 1.63 (m, 4H, 2CH_2_), 1.97–2.10 (dt, *J* = 14.6, 6.9 Hz, 2H, CH_2_), 2.31 (t, *J* = 7.6 Hz, 2H, CH_2_) 2.74 (t, *J* = 7.1 Hz, 4H, 2CH_2_), 4.41 (td, *J* = 8.6, 4.9 Hz, 1H, Gluα-CH), 6.49 (s, 2H, 2-NH_2_, exch), 6.82 (s, 1H, Ar.), 7.75 (d, *J* = 11.8 Hz, 1H, Ar.), 8.38 (s, 1H, Ar.), 8.75 (d, *J =* 7.8 Hz, 1H, CONH, exch.), 10.87 (s, 1H, 3-NH, exch.). HRMS (ESI) calculated for C_21_H_23_FN_5_O_6_ [M + H]^+^: 492.1348, found: 492.1344. HPLC analysis: retention time, 6.62 min; purity: 95.9%; eluent A, 0.1% formic acid in H_2_O: eluent B, 0.1% formic acid in ACN; gradient elution (95% H_2_O to 60% H_2_O) over 15 min with a flow rate of 0.5 mL/min and detected at 254 nm; column temperature, 25 °C.

##### (4-(4-(2-Amino-4-oxo-3,4-dihydrothieno[2,3-*d*]pyrimidin-6-yl)butyl)-3-fluorothiophene-2-carbonyl)-l-glutamic Acid **9**

Final compound **9** was prepared using the same general method reported for the preparation of **3**, **5**, and **9** from **15c** (200 mg, 0.38 mmol) to give 90 mg (46%) of **7** as a light-yellow powder. mp: 142.3–143.5 °C. ^1^H NMR (400 MHz, DMSO-*d*_6_): δ 1.56–1.67 (m, 4H, 2CH_2_), 1.92–2.13 (m, 2H, CH_2_), 2.32 (t, *J* = 7.4 Hz, 2H, CH_2_), 2.55 (d, *J* = 7.2 Hz, 2H, CH_2_), 2.77–2.99 (m, 2H, CH_2_), 4.36 (td, *J* = 8.4, 5.2 Hz, 1H, Gluα-CH), 6.48 (s, 2H, 2-NH_2_, exch.), 6.82 (s, 1H, Ar.), 7.52 (t, *J* = 4.8 Hz, 1H, Ar.), 7.99 (dd, *J* = 7.5, 3.8 Hz, 1H, CONH, exch.), 10.87 (s, 1H, 3-NH, exch.). HRMS (ESI) calculated for C_20_H_22_FN_4_O_6_S_2_ [M + H]^+^: 497.0959, found: 497.0955. HPLC analysis: retention time, 6.16 min; purity: 97.8%; eluent A, 0.1% formic acid in H_2_O: eluent B, 0.1% formic acid in ACN; gradient elution (95% H_2_O to 60% H_2_O) over 15 min with a flow rate of 0.5 mL/min and detected at 320 nm; column temperature, 25 °C.

#### General Procedure for the Synthesis of Alkyne Compounds **18a**–**d**

The appropriate bromo-pyridine/phenyl or thiophene ester (**17a**–**d**) (1.10 g, 5 mmol, 1 equiv) was combined with palladium chloride (35.5 mg, 0.82 mmol), triphenylphosphine (65 mg, 0.2 mmol, 0.04 equiv), copper iodide (152 mg, 0.8 mmol, 0.16 equiv), triethylamine (5.50 mL, 50 mmol), and 4-pentyn-1-ol (**16a**) or 5-hexyn-1-ol (**16b**) (7.5 mmol, 1.5 equiv) in a 20 mL microwave vial equipped with a stir bar and then dissolved in 10 mL of anhydrous acetonitrile. The reaction mixture was subjected to microwave irradiation at 100 °C for 1 h. After completion of the reaction, the reaction mixture was allowed to cool down to room temperature and transferred to a round-bottom flask to which silica gel (approximately 10 g) was added. After evaporating the solvent under reduced pressure, the resulting mixture was loaded onto a silica gel column and eluted with hexane followed by gradually increasing amounts of ethyl acetate, with up to 20% ethyl acetate in hexane. Fractions with the desired *R*_f_ (TLC) were pooled, and the solvent was removed under reduced pressure to yield **18a**–**d** as light-yellow liquids.

##### Methyl 5-(6-Hydroxyhex-1-yn-1-yl)picolinate (**18a**)

The preparation of intermediate **18a** from methyl 6-bromopicolinate **17a** (2.15 g, 20 mmol) was done following the general method described to synthesize target compounds **18a**–**d** to give a light-yellow liquid (1.63 g, 81%); TLC *R*_f_ = 0.25 (hexane/EtOAc, 1:1). ^1^H NMR (400 MHz, CDCl_3_): δ 1.72–1.80 (m, 4H, 2CH_2_), 2.50–2.57 (m, 2H, CH_2_), 3.72–3.77 (m, 2H, CH_2_), 4.03 (d, *J* = 0.6 Hz, 3H, CH_3_), 7.82 (ddd, *J* = 8.1, 2.1, 0.6 Hz, 1H, Ar.), 8.08 (dt, *J* = 8.0, 0.8 Hz, 1H, Ar.), 8.70–8.75 (m, 1H, Ar.).

##### Methyl 2-Fluoro-4-(5-hydroxypent-1-yn-1-yl)benzoate (**18b**)

The preparation of intermediate **18b** from methyl 4-bromo-2-fluorobenzoate **17b** (1.10 g, 4.76 mmol) was done following the general method described to synthesize target compounds **18a**–**d** to give a light-yellow liquid (0.9 g, 84%); TLC *R*_f_ = 0.26 (hexane/EtOAc, 1:1). ^1^H NMR (400 MHz, CDCl_3_): δ 1.85–1.96 (m, 2H, CH_2_), 2.59 (t, *J* = 7.0 Hz, 2H, CH_2_), 3.84 (*J* = 6.1 Hz, 2H, CH_2_), 3.95 (s, 3H, CH_3_), 7.14–7.24 (m, 2H, Ar.), 7.88 (t, *J* = 7.9 Hz, 1H, Ar.).

##### Methyl 2-Fluoro-4-(6-hydroxyhex-1-yn-1-yl)benzoate (**18c**)

The preparation of intermediate **18c** from methyl 4-bromo-2-fluorobenzoate **17c** (3.00 g, 12.87 mmol) was done following the general method to synthesize target compounds **18a**–**d** to give a light-yellow liquid (2.1 g, 66%); TLC *R*_f_ = 0.28 (hexane/EtOAc, 1:1). ^1^H NMR (400 MHz, CDCl_3_): δ 1.67–1.81 (m, 4H, CH_2_), 2.50 (t, *J* = 6.7 Hz, 2H, CH_2_), 3.72–3.76 (m*,* 2H, CH_2_), 3.95 (s, 3H, CH_3_), 7.14–7.24 (m, 2H, Ar.), 7.88 (t, *J* = 7.8 Hz, 1H, Ar.)

##### Methyl 3-Fluoro-4-(5-hydroxypent-1-yn-1-yl)thiophene-2-carboxylate (**18d**)

The preparation of intermediate **18d** from methyl 4-bromo-3-fluorothiophene-2-carboxylate **17d** (1.00 g, 4.52 mmol) was done following the general method described to synthesize target compounds **18a**–**d** to give a light-yellow liquid (0.81 g, 73%); TLC *R*_f_ = 0.25 (hexane/EtOAc, 1:1). ^1^H NMR (400 MHz, CDCl_3_): δ 1.83–1.97 (m, 2H, CH_2_), 2.57 (t, *J* = 7.0 Hz, 2H, CH_2_), 3.81–3.86 (m*,* 2H, CH_2_), 3.92 (s, 3H, CH_3_), 7.44 (d, *J* = 4.0 Hz, 1H, Ar.).

#### General Procedure for the Synthesis of Compounds **19a**–**d**

To synthesize **19a**–**d**, the Parr hydrogenation flask was charged with **18** (8.02 mmol), 10% palladium on activated carbon (50% w/w) (400 mg), and methanol (50 mL). Hydrogenation was performed for 12 h at 15–30 psi (**19a**) or for 24 h at 50 psi (**19b**–**d**). After completion of the reaction, the reaction mixture was filtered through Celite and washed repeatedly with methanol (100 mL). The filtrate was then concentrated under reduced pressure to afford **19a**–**d** as light-yellow liquids.

##### Methyl 5-(6-Hydroxyhexyl)picolinate (**19a**)

Using the general procedure described above, **19a** was obtained from **18a** as a light-yellow liquid (1.2 g, 59%); TLC *R*_f_ = 0.60 (hexane/EtOAc, 1:1).^1^H NMR (400 MHz, DMSO-*d*_6_): δ 1.13–1.36 (m, 4H, 2CH_2_), 1.40 (t, *J* = 6.6 Hz, 2H, CH_2_), 1.60 (p, *J* = 7.4 Hz, 2H, CH_2_), 2.64–2.72 (m, 2H, CH_2_), 3.37 (d, *J* = 5.2 Hz, 2H, CH_2_), 3.87 (s, 3H, CH_3_), 4.35 (t, *J* = 4.9 Hz, 1H, OH), 7.82 (dd, *J* = 8.0, 2.2 Hz, 1H, Ar.), 7.99 (dd, *J* = 8.0, 0.8 Hz, 1H, Ar.), 8.57 (d, *J* = 2.1, 1H, Ar.).

##### Methyl 2-Fluoro-4-(5-hydroxypentyl)benzoate (**19b**)

Using the general procedure described above, **19b** was obtained from **18b** (2.00 g, 8.47 mmol) as a light-yellow liquid (1.65 g, 77%); TLC *R*_f_ = 0.26 (hexane/EtOAc, 1:1).^1^H NMR (400 MHz, CDCl_3_): δ 1.37–1.48 (m, 2H, CH_2_), 1.61 (m, *J* = 6.6 Hz, 2H, CH_2_), 1.65–1.74 (m, 2H, CH_2_), 2.65–2.71 (m, 2H, CH_2_), 3.67 (t, *J* = 6.5 Hz, 2H, CH_2_), 3.94 (s, 3H, CH_3_), 6.95–7.05 (m, 2H, Ar.), 7.87 (t, *J* = 7.9 Hz, 1H, Ar.).

##### Methyl 2-Fluoro-4-(6-hydroxyhexyl)benzoate (**19c**)

Using the general procedure described above, **19c** was obtained from **18c** (2.00 g, 7.99 mmol) as a light-yellow liquid (1.74 g, 85%); TLC *R*_f_ = 0.28 (hexane/EtOAc, 1:1).^1^H NMR (400 MHz, CDCl_3_): δ 1.32–1.45 (m, 4H, 2CH_2_), 1.55–1.70 (m, 4H, 2CH_2_), 2.63–2.70 (m, 2H, CH_2_), 3.66 (t, *J* = 6.5 Hz, 2H, CH_2_), 3.94 (s, 3H, CH_3_), 6.94–7.05 (m, 2H, Ar.), 7.87 (t, *J* = 7.8 Hz, 1H, Ar.).

##### Methyl 3-Fluoro-4-(5-hydroxypentyl)thiophene-2-carboxylate (**19d**)

Using the general procedure described above, **19d** was obtained from **18d** (1.20 g, 4.68 mmol) as a light-yellow liquid (1.10 g, 90%); TLC *R*_f_ = 0.28 (hexane/EtOAc, 1:1).^1^H NMR (400 MHz, CDCl_3_): δ 1.47 (m, 2H, CH_2_), 1.89 (m, 4H, 2CH_2_), 2.57 (m, *J* = 7.0 Hz, 2H, CH_2_), 3.83 (m, 2H, CH_2_), 3.91 (s, 3H, CH_3_), 7.44 (t, *J* = 4.0 Hz, 1H, Ar.).

#### General Procedures for the Synthesis of **20a**–**d**

To compounds **19a**–**d** (1.5 g, 6.7 mmol, 1 equiv) dissolved in anhydrous dichloromethane (20 mL) in a round-bottom flask kept on ice, a stirred solution of Dess–Martin periodinane (DMP) (3.26 g, 7.0 mmol, 1.1 equiv) in dichloromethane (10 mL) was added, and the reaction mixture was warmed gradually from 0 °C to ambient temperature. When the reaction mixture was examined by TLC after ∼3 h, the TLC indicated the disappearance of **19a**–**d**. The reaction mixture was treated with dropwise addition of 10 mL 1 N NaOH solution to deactivate the residual DMP. Ethyl acetate was then used to extract the resulting solution three times, with each extraction using 30 mL of solvent. Fractions with the desired *R*_f_ (TLC) were pooled, and the solvent was removed under reduced pressure to afford **20a**–**d**.

##### Methyl 5-(6-Oxohexyl)picolinate (**20a**)

Using the general procedure described for **20a**–**d**, **20a** was obtained as a white solid (0.75 g, 50%); TLC *R*_f_ = 0.60 (hexane/EtOAc, 1:1). ^1^H NMR (400 MHz, CDCl_3_): δ 1.34–1.44 (m, 2H, CH_2_), 1.63–1.75 (m, 4H, 2CH_2_), 2.46 (td, *J* = 7.2, 1.6 Hz, 2H, CH_2_), 2.72 (t, *J* = 7.7 Hz, 2H, CH_2_), 4.02 (d, *J* = 0.9 Hz, 3H, CH_3_), 7.64–7.72 (m, 1H, Ar.), 8.09 (dd, *J* = 8.0, 0.8 Hz, 1H, Ar.), 8.59 (s, 1H, Ar.), 9.78 (q, *J* = 1.6, 1.2 Hz, 1H, CHO).

##### Methyl 2-Fluoro-4-(5-oxopentyl)benzoate (**20b**)

Using the general procedure described for **20a**–**d**, **20b** was obtained as a light-yellow liquid (1.11 g, 74%); TLC *R*_f_ = 0.70 (hexane/EtOAc, 1:1). ^1^H NMR (400 MHz, CDCl_3_): δ 1.63–1.72 (m, 2H, CH_2_), 1.79–1.96 (m, 2H, CH_2_), 2.12 (m, 2H, CH_2_), 2.49 (tt, *J* = 5.7, 1.5 Hz, 2H, CH_2_), 2.58–2.79 (m, 2H, CH_2_), 3.94 (s, 3H, CH_3_), 7.00 (dddd, *J* = 25.8, 11.9, 3.9, 1.7 Hz, 2H, Ar.), 7.85–7.91 (m, 1H, Ar.), 9.79 (t, *J* = 1.7 Hz, 1H, CHO).

##### Methyl 2-Fluoro-4-(6-oxohexyl)benzoate (**20c**)

Using the general procedure described for **20a**–**d**, **20c** was obtained as a light-yellow solid with impurities from DMP (1.26 g, crude yield 87%); TLC *R*_f_ = 0.70 (hexane/EtOAc, 1:1). ^1^H NMR (400 MHz, CDCl_3_): δ 1.38 (tt, *J =* 10.15, 6.38 Hz, 2H, CH_2_), 1.63–1.72 (m, 4H, 2CH_2_), 2.44–2.48 (dt, *J =* 7.30, 1.62 Hz, 2H, CH_2_), 2.65–2.69 (m, 2H, CH_2_), 3.94 (s, 3H, CH_3_), 6.94–6.98 (dd, *J =* 11.88, 1.34 Hz, 1H, Ar.), 7.01–7.03 (dd, *J =* 7.96, 1.44 Hz, 1H, Ar.), 7.85–7.89 (t, *J =* 7.81 Hz, 1H, Ar.), 9.79 (t, *J =* 1.62 Hz, 1H, CHO).

##### Methyl 3-Fluoro-4-(5-oxopentyl)thiophene-2-carboxylate (**20d**)

Using the general procedure described for **20a**–**d**, **20d** was obtained as a light-yellow liquid (0.84 g, 74%); TLC *R*_f_ = 0.70 (hexane/EtOAc, 1:1). ^1^H NMR (400 MHz, CDCl_3_): δ 1.67–1.70 (m, 4H, 2CH_2_), 2.49–2.53 (m, 2H, CH_2_), 2.59 (t, *J* = 6.8 Hz, 2H, CH_2_), 3.90 (s, 3H, CH_3_), 7.13 (d, *J* = 4.7 Hz, 1H, Ar.), 9.80 (t, *J* = 1.6 Hz, 1H, CHO).

#### General Procedures for the Synthesis of **21a**–**d**

The microwave-assisted Gewald reaction was carried out in a 20 mL microwave vial in the presence of aldehyde **20a**–**d** (6.18 mmol, 1 equiv), sulfur powder (300 mg, 9.3 mmol, 1.5 equiv), ethyl cyanoacetate (700 mg, 6.18 mmol, 1 equiv), ethanol (15 mL), and triethylamine (68 mg, 0.62 mmol, 0.1 equiv); the vial was sealed and subjected to microwave irradiation in the microwave reactor at 80 °C for 30 min. After completion of the reaction, unreacted sulfur was eliminated through filtration, and the filtrate was concentrated under reduced pressure. The orange liquid residue was loaded onto a silica gel column and subjected to elution with 10% ethyl acetate in hexane or directly used in the next step without further purification. Subsequently, the mixture of the appropriate thiophene (1 equiv) afforded by Gewald reaction was mixed with chloroformamidine hydrochloride (4 equiv) in DMSO_2_ and was heated at 140 °C for 4 h. After the completion of the reaction, 20 mL of water was added to the hot mixture, after which ammonium hydroxide was added dropwise to neutralize the suspension to pH over 7. The resulting brown solid was filtered, washed with water, and subsequently dried under a vacuum over P_2_O_5_. To a solution of the crude intermediate fractions dissolved in ethanol (10–50 mL), an aqueous solution of 1 N NaOH was added, and the reaction mixture was stirred at room temperature for 12 h. Ethanol was evaporated under reduced pressure, and the residue was dissolved in water (5–10 mL). TLC analysis showed one major spot at *R*_f_ = 0.25 (CHCl_3_/MeOH, 5:1). The reaction mixtures were dried under reduced pressure to yield residues that were subsequently dissolved in water. The resulting solutions were cooled in an ice bath, and the pH was adjusted to 3–4 by gradual addition of 1 N HCl dropwise to induce precipitation. The resulting suspension was left at 0 °C for 10 min, and then the residue was filtered, washed with water (5 mL), and dried under a vacuum over P_2_O_5_ at 50 °C to afford the free acids **21a**–**d**.

##### 5-(4-(2-Amino-4-oxo-3,4-dihydrothieno[2,3-*d*]pyrimidin-6-yl)butyl)picolinic Acid (**21a**)

The general method described for the preparation of **21a**–**d** was used to prepare **21a** (0.110 g, 45% over 3 steps) as a brown solid; mp: decomposes at 250 °C before melting. ^1^H NMR (400 MHz, DMSO-*d*_6_): δ 1.63 (m, 4H, CH_2_), 2.74 (t, *J* = 9.3 Hz, 4H, CH_2_), 6.49 (s, 2H, 2-NH_2_, exch.), 6.81 (s, 1H, Ar.), 7.80 (dd, *J* = 8.2, 2.2 Hz, 1H, Ar.), 8.19 (dd, *J* = 26.6, 8.0 Hz, 1H, Ar.), 8.59 (dd, *J* = 30.4, 2.1 Hz, 1H, Ar.), 10.88 (s, 1H, 3-NH, exch.).

##### 4-(3-(2-Amino-4-oxo-3,4-dihydrothieno[2,3-*d*]pyrimidin-6-yl)propyl)-2-fluorobenzoic Acid (**21b**)

The general method described for the preparation of **21a**–**d** was used to prepare **21b** (0.110 g, 21%) as a light-brown solid; mp >250 °C. ^1^H NMR (400 MHz, DMSO-*d*_6_): δ 1.92 (t, *J* = 7.7 Hz, 2H, CH_2_), 2.66–2.75 (m, 4H, CH_2_), 6.53 (s, 2H, 2-NH_2_, exch.), 6.83 (s, 1H, Ar.), 7.15 (t, *J* = 8.7 Hz, 2H, Ar.), 7.77 (t, *J* = 8.0 Hz, 1H, Ar.), 10.95 (s, 1H, 3-NH, exch.).

##### 4-(4-(2-Amino-4-oxo-3,4-dihydrothieno[2,3-*d*]pyrimidin-6-yl)butyl)-2-fluorobenzoic Acid (**21c**)

The general method described for the preparation of **21a**–**d** was used to prepare **21c** (0.180 g, 20%) as a light-brown solid; mp >250 °C. ^1^H NMR (400 MHz, DMSO-*d*_6_): δ 1.62 (dd, *J* = 13.0, 7.4 Hz, 4H, 2CH_2_), 2.65 (t, *J* = 7.0 Hz, 2H, CH_2_), 2.73 (t, *J* = 6.6 Hz, 2H, CH_2_), 6.56 (s, 2H, NH_2,_ exch.), 6.80 (s, 1H, CH, Ar.), 7.04 (t, *J* = 8.7 Hz, 2H, Ar.), 7.72 (t, *J* = 7.9 Hz, 1H, Ar.), 10.97 (s, 1H, 3-NH exch.).

##### 4-(3-(2-Amino-4-oxo-3,4-dihydrothieno[2,3-*d*]pyrimidin-6-yl)propyl)-3-fluorothiophene-2-carboxylic Acid (**21d**)

The general method described for the preparation of **21a**–**d** was used to prepare **21d** (0.145 g, 29%) as a light-brown solid; mp >250 °C. ^1^H NMR (400 MHz, DMSO-*d*_6_): δ 1.87 (p, *J* = 7.5 Hz, 2H, CH_2_), 2.48 (d, *J* = 7.0 Hz, 2H, CH_2_), 2.74 (t, *J* = 7.4 Hz, CH_2_), 6.75 (s, 2H, NH_2,_ exch.), 6.84 (s, 1H, CH, Ar.), 7.24 (d, *J* = 4.1 Hz, 1H, Ar.), 10.32 (s, 1H, 3-NH exch.).

#### General Procedure for the Synthesis of Target Compounds **22a**–**d**

The crude acids **21a**–**d** (1 equiv) were mixed with *N*-methylmorpholine (1.2 equiv) and 2-chloro-4,6-dimethoxy-1,3,5 triazine (1.2 equiv) and dissolved in anhydrous DMF, and the reaction mixture was stirred at room temperature for 2 h. *N*-Methylmorpholine (1.2 equiv) and l-glutamate diethyl ester hydrochloride or l-glutamate dimethyl ester hydrochloride (1.8 equiv) were then added, and the reaction mixture was stirred for an additional 4 h. Upon completion of the reaction, DMF was removed under a vacuum followed by addition of MeOH and silica gel. The solution was evaporated to get a plug, which was subjected to column chromatography on the silica gel with CHCl_3_ in MeOH (CHCl_3_/MeOH, 5:1) as the eluent. Fractions with the desired *R*_f_ (TLC) were pooled, and the solvent was removed under reduced pressure to afford the intermediate glutamate esters **22a**–**d** as semisolids.

##### Diethyl (5-(4-(2-Amino-4-oxo-3,4-dihydrothieno[2,3-*d*]pyrimidin-6-yl)butyl)picolinoyl)-l-glutamate (**22a**)

The general method described for the preparation of **22a**–**d** was used to prepare **22a** (80%) as a yellow solid from **21a** (0.2 g, 0.55 mmol). mp: 167.5–167.9 °C TLC *R*_f_ 0.40 (MeOH/CHCl_3_, 1:6); ^1^H NMR (400 MHz, DMSO- *d*_6_): δ 1.16 (dq, *J* = 21.2, 7.0, 5.6 Hz, 6H, 2CH_3_), 1.65 (d, *J* = 13.4 Hz, 4H, 2CH_2_), 2.13 (d, *J* = 28.6 Hz, 2H, CH_2_), 2.38 (m, 2H, CH_2_), 2.74 (m, 4H, 2CH_2_), 3.91–4.26 (m, 4H, 2CH_2_), 4.53 (m, 1H, Gluα-CH), 6.47 (s, 2H, 2-NH_2_ exch.), 6.82 (s, 1H, Ar.), 7.85 (s, 1H, Ar.), 7.95 (d, *J* = 7.9 Hz, 1H, Ar.), 8.53 (d, *J* = 2.2 Hz, 1H, Ar.), 8.92 (d, *J* = 8.8 Hz, 1H, CO-NH, exch.), 10.89 (s, 1 H, 3-NH exch.)

##### Dimethyl (4-(3-(2-Amino-4-oxo-3,4-dihydrothieno[2,3-*d*]pyrimidin-6-yl)propyl)-2-fluorobenzoyl)-l-glutamate (**22b**)

The general method described for the preparation of **22a**–**d** was used to prepare **22b** (46%) as a yellow semisolid from **21b** (0.2 g, 0.60 mmol). TLC *R*_f_ 0.42 (MeOH/CHCl_3_, 1:6); ^1^H NMR (400 MHz, DMSO-*d*_6_): δ 1.86–2.14 (m, 4H, 2CH_2_), 2.45 (t, *J* = 7.2 Hz, 2H,CH_2_), 2.70 (t, *J* = 8.1 Hz, 4H, 2CH_2_), 3.59 (s, 3H, CH_3_), 3.66 (s, 3H, CH_3_), 4.45 (m, 1H, Gluα-CH), 6.49 (s, 2H, 2-NH_2_ exch.), 6.84 (s, 1H, Ar.), 7.12–7.14 (m, 2H, 2Ar.), 7.53 (t, *J* = 7.7 Hz, 1H, Ar.), 8.62 (d, *J* = 7.5 Hz, 1H, CO-NH, exch.), 11.88 (s, 1 H, 3-NH exch.).

##### Dimethyl (4-(4-(2-Amino-4-oxo-3,4-dihydrothieno[2,3-*d*]pyrimidin-6-yl)butyl)-2-fluorobenzoyl)-l-glutamate (**22c**)

The general method described for the preparation of **22a**–**d** was used to prepare **22c** (49%) as a yellow semisolid from **21c** (0.2 g, 0.55 mmol). TLC *R*_f_ 0.42 (MeOH/CHCl_3_, 1:6); ^1^H NMR (400 MHz, DMSO-*d*_6_): δ 1.65 (m, 4H, 2CH_2_), 2.34 (t, 2H, CH_2_), 2.45 (m, 2H, CH_2_), 2.56 (m, 2H, CH_2_), 2.68 (m, 2H, CH_2_), 3.59 (s, 3H, CH_3_), 3.67 (s, 3H, CH_3_), 4.45 (m, 1H, Gluα-CH), 6.47 (s, 2H, 2-NH_2_ exch.), 6.80 (s, 1H, C6-CH), 7.12–7.14 (m, 2H, 2Ar.), 7.51 (t, 1H, Ar.), 8.62 (d, *J* = 7.5 Hz, 1H, CO-NH, exch.), 10.91 (s, 1 H, 3-NH exch.).

##### Dimethyl (4-(3-(2-Amino-4-oxo-3,4-dihydrothieno[2,3-*d*]pyrimidin-6-yl)propyl)-3-fluorothiophene-2-carbonyl)-l-glutamate (**22d**)

The general method described for the preparation of **22a**–**d** was used to prepare **22d** (44%) as a yellow semisolid from **21d** (0.2 g, 0.55 mmol). TLC *R*_f_ 0.45 (MeOH/CHCl_3_, 1:6); ^1^H NMR (400 MHz, DMSO-*d*_6_): δ 1.90 (m, 2H, CH_2_), 2.41 (t, *J* = 7.4 Hz, 2H, CH_2_), 2.76 (m, 6H, 3CH_2_), 3.58 (s, 3H, CH_3_), 3.65 (s, 3H, CH_3_), 4.44 (m, 1H, Gluα-CH), 6.53 (s, 2H, 2-NH_2_ exch.), 6.85 (s, 1 H, Ar.), 7.57 (d, *J* = 4.8 Hz, 1H, Ar.), 8.20 (dd, *J* = 7.0, 1 H, CONH, exch.), 10.91 (s, 1H, 3-NH exch.).

#### General Procedure for the Synthesis of Target Compounds **4**, **6**, **7**, and **8**

To hydrolyze the glutamate esters, intermediates **22a**–**d** were dissolved in 1 N NaOH in methanol, and the resulting solution was stirred at room temperature. After 4 h, TLC analysis indicated the complete consumption of the starting material and generation of one major spot at the baseline (CHCl_3_/MeOH 8:1). The solution was cooled in an ice bath, and the pH was adjusted to 3–4 by gradual addition of 1 N HCl. The resulting suspension was filtered, and the residue was washed with a small quantity of cold water before drying in vacuo over P_2_O_5_ to afford the target compounds **4**, **6**, **7**, and **8** as light-yellow powders.

##### (5-(4-(2-Amino-4-oxo-3,4-dihydrothieno[2,3-*d*]pyrimidin-6-yl)butyl)picolinoyl)-l-glutamic Acid (**4**)

Final compound **4** was prepared from **22a** (150 mg, 0.28 mmol) using the general method utilized for the preparation of **4**, **6**, **7**, and **8** to give 125 mg (95%) of **4** as a white powder. mp: 226.6–226.8 °C. ^1^H NMR (400 MHz, DMSO-*d*_6_): δ 1.55–1.72 (m, 4H, 2CH_2_), 1.93–2.19 (m, 2H, CH_2_), 2.29 (t, *J* = 7.6 Hz, 2H, CH_2_), 2.74 (q, *J* = 7.0 Hz, 4H, 2CH_2_), 4.48 (td, *J* = 8.8, 4.7 Hz, 1H, Gluα-CH), 6.48 (s, 2H, 2-NH_2_, exch.), 6.82 (s, 1H, Ar.), 7.84 (dd, *J* = 8.1, 2.2 Hz, 1H, Ar.), 7.95 (d, *J* = 7.9 Hz, 1H, Ar.), 8.52 (d, *J* = 2.0 Hz, 1H, Ar.), 8.80 (d, *J* = 8.2 Hz, 1H, CONH, exch.), 10.86 (s, 1H, 3-NH, exch.). HRMS (ESI) calculated for C_21_H_24_N_5_O_6_S [M + H]^+^: 474.1442, found: 474.1439. HPLC analysis: retention time, 6.0 min; eluent A, 0.1% of formic acid in H_2_O; eluent B, 0.1% of formic acid in CAN, purity: 98.5%; gradient condition (5% ACN to 40% ACN) over 15 min with a flow rate of 0.5 mL/min and detected at λ = 320 nm, column temperature, 25 °C.

##### (4-(3-(2-Amino-4-oxo-3,4-dihydrothieno[2,3-*d*]pyrimidin-6-yl)propyl)-2-fluorobenzoyl)-l-glutamic Acid (**6**)

Final compound **6** was prepared from **22b** (100 mg, 0.19 mmol) using the general method described for the preparation of **4**, **6**, **7**, and **8** to give 85 mg (88%) of **6** as a light-yellow powder. mp: 128.0–129.8 °C. ^1^H NMR (400 MHz, DMSO-*d*_6_): δ 1.91 (d, *J* = 7.6 Hz, 2H, CH_2_), 1.94–2.16 (m, 2H, CH_2_), 2.34 (t, *J* = 7.5 Hz, 2H, CH_2_), 2.70 (dt, *J* = 13.4, 7.8 Hz, 4H, 2CH_2_), 4.44–4.35 (m,1H, Gluα-CH), 6.50 (s, 2H, 2-NH_2_, exch.), 6.83 (s, 1H, Ar.), 7.15 (d, *J* = 9.8 Hz, 2H, Ar.), 7.54 (t, *J* = 7.7 Hz, 1H, Ar.), 8.44 (d, *J =* 7.7 Hz, 1H, CONH, exch.), 10.91 (s, 1H, 3-NH, exch.). HRMS (ESI) calculated for C_21_H_22_FN_4_O_6_S [M + H]^+^: 477.1239, found: 477.1232. HPLC analysis: retention time, 5.96 min; purity: 96.5%; eluent A, 0.1% formic acid in H_2_O: eluent B, 0.1% formic acid in ACN; gradient condition (5% ACN to 40% ACN) over 15 min with a flow rate of 0.5 mL/min and detected at λ = 320 nm, column temperature, 25 °C.

##### (4-(4-(2-Amino-4-oxo-3,4-dihydrothieno[2,3-*d*]pyrimidin-6-yl)butyl)-2-fluorobenzoyl)-l-glutamic Acid (**7**)

Final compound **7** was prepared from **22c** (100 mg, 0.19 mmol) using the general method described for the preparation of **4**, **6**, **7**, and **8** to give 78 mg (82%) of **7** as a light-yellow powder. mp: 121.7–122.6 °C. ^1^H NMR (400 MHz, DMSO-*d*_6_): δ 1.62 (m, 4H, 2CH_2_), 1.90–2.07 (dq, *J* = 13.3, 6.9 Hz, 2H, CH_2_), 2.35 (t, *J* = 7.7 Hz, 2H, CH_2_), 2.67 (t, *J* = 7.0 Hz, 2H, CH_2_), 2.73 (t, *J* = 6.8 Hz, 2H, CH_2_), 4.38 (m, *J* = 8.6, 4.8 Hz, 1H, Gluα-CH), 6.48 (s, 2H, 2-NH_2_, exch.), 6.80 (s, 1H, Ar.), 7.13 (d, *J* = 9.1 Hz, 2H, Ar.), 7.53 (t, *J* = 7.8 Hz, 1H, Ar.), 8.43 (d, *J =* 7.8 Hz, 1H, CONH, exch.), 10.87 (s, 1H, 3-NH, exch.). HRMS (ESI) calculated for C_22_H_24_FN_4_O_6_S [M + H]^+^: 491.1395, found: 491.1388. HPLC analysis: retention time, 6.70 min; purity: 96.1%; eluent A, 0.1% formic acid in H_2_O: eluent B, 0.1% formic acid in ACN; gradient elution (5% ACN to 40% ACN) over 15 min with a flow rate of 0.5 mL/min and detected at λ = 320 nm; column temperature, 25 °C.

##### (4-(3-(2-Amino-4-oxo-3,4-dihydrothieno[2,3-*d*]pyrimidin-6-yl)propyl)-3-fluorothiophene-2-carbonyl)-l-glutamic Acid (**8**)

Final compound **8** was prepared from **22d** (150 mg, 0.45 mmol) using the general method described for the preparation of **4**, **6**, **7**, and **8** to give 84 mg (88%) of **8** as a light-yellow powder. mp: 146.2–146.8 °C. ^1^H NMR (400 MHz, DMSO-*d*_6_): δ 1.84–2.16 (m, 6H, 3CH_2_), 2.32 (t, *J* = 7.3 Hz, 2H, CH_2_), 2.76 (t, *J* = 7.4 Hz, 2H, CH_2_), 4.37 (dq, *J* = 8.5, 5.0 Hz, 1H, Gluα-CH), 6.49 (s, 2H, 2-NH_2_, exch.), 6.86 (s, 1H, Ar.), 7.57 (t, *J* = 4.6 Hz, 1H, Ar.), 7.99 (dd, *J* = 7.8, 3.7 Hz, 1H, CONH, exch.), 10.87 (s, 1H, 3-NH, exch.), 12.52 (s, 2H, 2COOH-exch). HRMS (ESI) calculated for C_19_H_20_FN_4_O_6_S_2_ [M + H]^+^: 483.0803, found: 483.0795. HPLC analysis: retention time, 5.95 min; purity: 95.7%; eluent A, 0.1% formic acid in H_2_O: eluent B, 0.1% formic acid in ACN; gradient condition (5% ACN to 40% ACN) over 15 min with a flow rate of 0.5 mL/min and detected at λ = 320 nm, column temperature, 25 °C.

## Biological Studies

### Reagents

[2,3,3-^2^H]l-Serine (98%) was purchased from Cambridge Isotope Laboratories, Inc. (Andover, MA). Folic acid was purchased from Sigma Chemical Co. (St. Louis, MO). MTX and leucovorin [(6*R*,*S*)5-formyl THF] were obtained from the Drug Development Branch, National Cancer Institute (Bethesda, MD). PMX [*N*-{4-[2-(2-amino-3,4-dihydro-4-oxo-7*H*-pyrrolo[2,3-*d*]pyrimidin-5-yl)ethyl]benzoyl-l-glutamic acid] (Alimta) was purchased from LC Laboratories (Woburn, MA). 10-CHOTHF was purchased from Merck & Cie (Schaffhausen, Switzerland). Serine-, glycine-, and folate-free RPMI 1640 was purchased from ThermoFisher (Waltham, MA) and supplemented with tissue-culture grade glycine (ThermoFisher) or serine (Sigma-Aldrich). Additional chemicals were purchased from commercial sources in the highest available purity. Compounds **1**, **2**, **10**, **11**, **AGF23**, and **AGF347** were synthesized as previously described.^25,26,32,45^

### Cell Culture

The MTXRIIOua^R^2-4 RFC-, PCFT-, and FRα-null Chinese hamster ovary (CHO) cell line (R2) was a gift from Dr. Wayne Flintoff (University of Western Ontario).^40,42^ Isogenic CHO cell lines were subsequently derived from R2 cells by transfection with RFC, PCFT, or FRα cDNAs to generate PC43-10 (expresses only human RFC), R2/PCFT4 (expresses only human PCFT), RT16 (expresses only human FRα), and D4 (expresses only human FRβ).^25,26,39^

The CHO sublines were cultured in α-minimal essential medium (α-MEM) supplemented with 100 U/mL penicillin/100 μg/mL streptomycin, 2 mM l-glutamine, and 10% bovine calf serum (Sigma-Aldrich). For the PC43-10, R2/PCFT4, RT16, and D4 sublines, 1.5 mg/mL G418 was also added. Seventy-two hours prior to *in vitro* proliferation assays (below), RT16 and D4 CHO cells were grown in folate-free RPMI with dialyzed fetal bovine serum (DFBS) (Invitrogen) supplemented with 100 U/mL penicillin/10 μg/mL streptomycin and 2 mM l-glutamine. FRα-expressing KB nasopharyngeal carcinoma cells were acquired from the American Type Culture Collection (Manassas, VA); cells were grown in complete folate-free RPMI 1640 supplemented with 10% fetal bovine serum, 100 U/mL penicillin/100 μg/mL streptomycin, and 2 mM l-glutamine. Cell lines were authenticated by STR analysis (Genetica DNA Laboratories, Burlington, NC) and tested for *Mycoplasma* by PCR (Venor GeM Mycoplasma Detection Kit, Sigma). Frozen stocks were generated from authenticated *Mycoplasma*-free cultures.

Cell proliferation assays used CHO and KB cells cultured in folate-free-RPMI 1640 media with 10% DFBS, 2 mM l-glutamine, and 100 U/mL penicillin/100 μg/mL streptomycin supplemented with 2 nM (KB, RT16, D4) or 25 nM (R2, PC43-10, R2/PCFT4) leucovorin.^25,26,30^ Parallel incubations with 200 nM folic acid were performed in viability assays to confirm FR-mediated drug uptake. Cells were plated in 96-well dishes at 2500–5000 cells/well in 200 μL media and treated with a range of C1 inhibitors spanning 0 to 1 μM. Cells were treated at 37 ° C with 5% CO_2_, and numbers were assayed after 96 h using CellTiter-blue (Promega, Madison, WI) and a fluorescence plate reader.^25,26^ Raw data were exported to Excel, and the results were plotted using Prism GraphPad 6.0 to determine IC_50_ values corresponding to the drug concentrations that resulted in 50% loss of cell growth.

Additional proliferation assays with metabolite rescue used KB cells to identify the targeted pathway and enzyme(s) in folate- and glycine-free RPMI1640, DFBS, 2 mM glutamine, and antibiotics. Cells were treated with inhibitors in the presence of thymidine (10 μM), adenosine (60 μM), glycine (130 μM), AICA (320 μM), combined adenosine/glycine, or combined AICA/glycine. These assays have been previously described.^25,26,45^

### *In Vitro* Targeted Metabolomics

KB cells (1 × 10^6^) were seeded in each of three 60 mm dishes in folate-, serine-, and glycine-free RPMI1640 supplemented with 10% DFBS, 1% penicillin/streptomycin, 2 mM l-glutamine, 285 μM serine, 130 μM glycine, and 2 nM leucovorin. Cells were allowed to adhere for 24 h at 37^o^ C, after which fresh media were added including 60 μM adenosine and 100 nM of **AGF23**, **AGF347**, or compounds **1**, **2**, or **6** (in 100% DMSO). Control cells were treated with an equivalent volume of DMSO (vehicle). After an additional 24 h, fresh media were added including the inhibitors and 250 μM [2,3,3-^2^H]serine. The cells were incubated for 24 h; metabolites were extracted with methanol/water (80:20 v/v); and the supernatant was collected, dried in a vacuum evaporator at 4 °C, and reconstituted in water for LC–MS/MS analysis.^45^ The targeted metabolites were quantitatively determined using the AB Sciex QTRAP 6500 LC–MS/MS system. Serine and glycine were separated on an ACQUITY UPLC BEH amide column (2.1 × 50 mm, 1.7 μm) using a gradient of mobile phase A (10 mM ammonium acetate in water, pH 3) and mobile phase B (0.1% formic acid in acetonitrile). Isotopomers were detected using multiple reaction monitoring (MRM) at the positive ionization mode. Metabolites were identified by their exact masses, and their retention times were compared against the retention times of standard metabolites using the MultiQuant 3.0.1 software. Absolute metabolite concentrations were calculated from calibration curves and then normalized to total protein concentrations measured from the postextraction pellet (solubilized in 0.5 N NaOH) using the Folin-phenol method.^53^ Values were square root transformed and compared to the vehicle control by Welch’s unpaired *t* test. To correct for multiple comparisons, adjusted *p* values were determined using Holm’s method.

### Enzyme Expression and Purification

N-terminal hexahistidine-tagged GARFTase (formyltransferase domain; residues 808–1010) and full-length human ATIC were expressed in Rosetta (DE3)pLysS cells.^45,48^ Cultures (1 L) were grown at 37 °C in LB media with 100 μg/mL ampicillin and 34 μg/mL chloramphenicol until OD_600_ reached 0.6. Isopropyl β-d-1-thiogalactopyranoside (500 μM) was added, and the cells were incubated at 20 °C for 16–18 h. The cultures were pelleted and resuspended in 40 mL of 25 mM Tris–HCl, pH 7.5, 300 mM NaCl, 5 mM β-mercaptoethanol (β-ME), 10 mM CaCl_2_, 10 mM MgCl_2_, 40 mg lysozyme, and 8 U DNAse I (Sigma). The cells were lysed by emulsification.

His-GARFTase was purified from the lysate by ÄKTA FPLC (GE Healthcare) with a Ni-NTA column (Qiagen, Valencia, CA).^48^ The column was washed with 5 column volumes (CV) of 25 mM Tris–HCl, pH 8, 300 mM NaCl, 10% glycerol, and 5 mM β-ME. His-GARFTase was eluted with a 0 to 75% gradient of 25 mM Tris–HCl, pH 8, 300 mM NaCl, 10% glyercol, 300 mM imidazole, and 5 mM β-ME over 10 CV. Further purification involved size exclusion chromatography on a Superdex 75 16/60 (GE Healthcare) column with 25 mM Tris–HCl, pH 8, 10 mM β-ME, and 300 mM NaCl. Purified His-GARFTase was stored at 90 μM in 20% glycerol at −80 °C.

His-ATIC was purified from the lysate by immobilized metal affinity chromatography with a 4 mL Ni-NTA column (Gold Biotechnology).^45^ The column was washed with 5 CV of 25 mM Tris–HCl, pH 7.5, 300 mM NaCl, 10 mM imidazole, and 5 mM β-ME (wash buffer) followed by 5 CV of the wash buffer containing 25 mM imidazole. The protein was eluted with 5 CV of the elution buffer containing components of the wash buffer with 300 mM imidazole. Further purification was by ÄKTA FPLC (GE Healthcare) with a Superdex 200 16/60 (GE Healthcare) column equilibrated with 20 mM Tris–HCl, pH 7.5, 150 mM NaCl, 50 mM KCl, 5 mM EDTA, and 5 mM dithiothreitol (DTT). His-ATIC was stored at 150 μM at 4 °C in this buffer (up to 6 months) or at 100 μM with 20% glycerol at −80 °C for longer-term storage.

N-terminal His_6_-tagged SHMT2 (residues 30–504) and full-length MTHFD2 were expressed in Rosetta (DE3)pLysS cells. One liter cultures were grown in LB media with 100 mg/mL ampicillin and 35 mg/mL chloramphenicol at 37 °C until the OD_600_ was 0.6. To induce expression, 500 mM isopropyl β-d-1-thiogalactopyranoside was added, and the cells were grown for 16–18 h at 18 °C. The cells were pelleted and resuspended in 25 mL of 25 mM Tris–HCl, pH 7.5, 300 mM NaCl, and 5 mM β-Me. To lyse the cells, 40 mg lysozyme and 8 U DNAse I were added followed by incubation on ice for 15 min. The cells were then lysed by emulsification.

Purification of His-SHMT2 and His-MTHFD2 was by Ni-affinity chromatography (above). For further purification of His-SHMT2, pyridoxal 5′-phosphate (PLP) was added (3-fold excess to His-SHMT2) followed by incubation for 16–18 h. Further purification was by size exclusion chromatography on a Superdex 200 16/60 column (GE Healthcare) with monitoring of PLP absorbance at 435 nm. Purified His-SHMT2 was stored in 20 mM sodium phosphate buffer, pH 7.5, 100 mM potassium chloride, 0.2 mM EDTA, and 5 mM β-ME. To further purify MTHFD2, size exclusion chromatography was performed using a Superdex 200 16/60 column (GE Healthcare). His-MTHFD2 was stored in 50 mM Tris–HCl buffer, pH 7.5, 250 mM sodium chloride, 5% glycerol, and 0.5 mM tris(2-chloroethyl) phosphate (TCEP).

### *In Vitro* Enzymatic Assays and *K*_i_ Determinations

GARFTase catalytic activity was measured by monitoring THF formation from 10-CHOTHF.^32,45,48^ GARFTase assays included 40 μM 10-CHO-THF, 50 nM His-GARFTase, 15 μM α,β-GAR, and a range of inhibitor concentrations in 25 mM Tris–HCl, pH 8.0, 300 mM NaCl, and 5 mM β-ME at 37 °C.^45^ AICARFTase catalytic activity was measured by monitoring THF formation from 10-CHOTHF.^54^ Reactions included 50 μM 10-CHOTHF, 100 nM His-ATIC, 50 μM ZMP, and inhibitors in 32.6 mM Tris–HCl, pH 7.5, 25 mM KCl, and 5 mM β-ME at 25 ° C.^45^ Kinetic measurements were recorded in triplicate in a UV-transparent 96-well plate (Costar 3635) at 298 nm using a BioTek Synergy Neo2 Plate Reader. Initial slopes were graphed against inhibitor concentrations and fit to a three-parameter nonlinear regression to calculate IC_50_ values for each compound (GraphPad Prism 8.0). *K*_i_ values were calculated from the IC_50_ values [*K*_i_ = IC_50_/([10-CHOTHF]/*K*_M_ + 1)] using previously determined *K*_M_ values of 10-CHOTHF with His-GARFTase or His-ATIC of 84.8 and 100 μM, respectively. SHMT2 activity was assayed by monitoring NADH production with a coupled assay including 500 nM His-SHMT2 and 10 μM His-MTHFD2 (1:200 molar ratio His-SHMT2/His-MTHFD2). NADH production by His-MTHFD2 was monitored by fluorescence at 470 nm with excitation at 360 nm (Synergy Neo2 Biotek plate reader). Inhibition constants (*K*_i_’s) were calculated from the IC_50_ values using the equation *K*_i_ = IC_50_/([*S*]/*K*_M_ + 1) and the *K*_m_ and substrate concentration for THF. The THF concentration in the reaction was 50 mM. The calculated *K*_m_ value for THF with His-SHMT2 was 62.8 μM.

### Crystallization of Human GARFTase, X-ray Data Collection, and Structure Determination

The GAR formyltransferase domain (residues 808–1010) of the trifunctional GARFTase, glycinamide ribonucleotide synthase (GARS), and aminoimidazole ribonucleotide synthetase (AIRS) enzyme (engineered with a noncleavable C-terminal hexahistidine tag; GARFTase-His) was buffer-exchanged into 25 mM Tris–HCl, pH 8.0, 200 mM NaCl, and 0.6 mM TCEP and concentrated to 10 mg/mL. GARFTase-His was incubated (4 °C, 30 min) in a 3-fold molar ratio with α,β-GAR in the presence or absence of inhibitors and incubated at 4 °C. For crystal screens, hanging drop plates contained 1 μL of protein–ligand solution, 0.8 μL of crystal condition, and 0.2 μL of 9 mM *N*-decyl-β-d-thiomaltoside (Hampton Research, Aliso Viejo, CA) equilibrated over 0.5 mL of the crystallant (0.1 M Tris–HCl, pH 7.5, 0.33 M NaCl, 16–21% polyethylene glycol (PEG) 4000, and 2% PEG 400). Cube-shaped crystals formed within a few days; crystals were frozen by direct immersion in liquid nitrogen after being transferred stepwise to the crystallant with 35% PEG 4000. In some cases, inhibitors were soaked into GARFTase-His/α,β-GAR complex crystals; crystals were transferred to the cryoprotectant and allowed to soak in a 3:3:1 α,β-GAR/inhibitor/GARFTase-His molar ratio for at least 30 min prior to flash freezing.

Data collection was performed at the Lawrence Berkeley National Laboratory Advanced Light Source beamline 4.2.2 using the Taurus CMOS detector. All data sets were processed in space group *P*3_2_2 (XDS^55,56^). Molecular replacement was performed using Protein Data Bank (PDB) entry 1J9F with waters and ligands removed as a search model (PHENIX^57^). Subsequent model building and refinement used Coot^58^ and PHENIX,^57^ respectively. Data collection and refinement statistics are in Table S2 (Supporting Information).

### Statistical Analysis

Data were checked for their distributional assumptions and, if needed, transformed to meet the normality assumption. Statistical comparisons were performed using two-sided, unpaired *t* tests after square root transformation. Holm’s method was used to adjust for multiplicity. Statistical analyses were carried out using R and GraphPad Prism.

## Accession Codes

Atomic coordinates and experimental data for crystallographic structures have been deposited in the Protein Data Bank with accession numbers **8FDY** (GARFTase/GAR/**4**), **8EF0** (GARFTase/GAR/**5**), **8FDZ** (GARFTase/GAR/**9**), and **8FDX** (GARFTase/GAR/**10**). The authors will immediately release the atomic coordinates upon publication.

## Supporting Information Available

Docked scores for 6-substituted thieno[2,3-*d*]pyrimidine antifolates (Table S1); data collection and refinement statistics for GARFTase crystal structures in complex with 6-substituted thieno[2,3-*d*]pyrimidines and β-GAR (Table S2); ^1^H NMR spectra, HPLCs, and mass spectra of final compounds **3**-**9** (Figure S1); D_2_O exchange studies of representative example **9** (Figure S2); growth inhibition of KB human tumor cells by thieno[2,3-*d*]pyrimidine analogs and the protective effects of nucleosides, glycine, and/or AICA (Figure S3); crystal structure of **4** bound in the folate binding pocket of GARFTase (Figure S4); crystal structure of **10** bound in the folate binding pocket of GARFTase (Figure S5); crystal structure of **9** bound in the folate binding pocket of GARFTase (Figure S6); and crystal structure of **5** bound in the folate binding pocket of GARFTase (Figure S7) (PDF)

## Abbreviations

10-CHOTHF: 10-formyl tetrahydrofolate
CD: 3-dimensional
ADMET: absorption, distribution, metabolism, excretion, and toxicity
ACN: acetonitrile
AICA: 5-aminoimidazole-4-carboxamide
ATIC: 5-aminoimidazole-4-carboxamide ribonucleotide formyl transferase/inosine monophosphate cyclohydrolase
AICARFTase: 5-aminoimidazole-4-carboxamide ribonucleotide formyltransferase
CHO: Chinese hamster ovary
CHCl: chloroform
CV: column volume
DMP: Dess–Martin periodinane
CDCl_3_: deuterated chloroform
DMSO-*d_6_*: deuterated dimethyl sulfoxide
DFBS: dialyzed fetal bovine serum
DCM: dichloromethane
CDMT: 2,4-dimethoxy-6-chloro-triazine
DMF: dimethylformamide
DMSO_2_: dimethyl sulfone
DTT: dithiothreitol
DMEM: Dulbecco’s minimal essential medium
DPBS: Dulbecco’s phosphate-buffered saline
EtOAc: ethyl acetate
FBS: fetal bovine serum
FR: folate receptor
FDA: Food and Drug Administration
GAR: glycinamide ribonucleotide
GARFTase: glycinamide ribonucleotide formyltransferase
HBSS: Hanks’ balanced salts solution
HBS: HEPES-buffered saline
HPLC: high-performance liquid chromatography
HRMS: high-resolution mass spectrometry
IC_50_: 50% inhibitory concentration
IUPAC: International Union of Pure and Applied Chemistry
LCV: leucovorin
LC–MS: liquid chromatography–mass spectrometry
MHz: megahertz
MeOH: methanol
MTX: methotrexate
MTHFD1L: methylene tetrahydrofolate dehydrogenase 1-like
MTHFD2: methylene tetrahydrofolate dehydrogenase 2
MEM: minimal essential media
NMM: *N*-methyl morpholine
MRM: multiple reaction monitoring
NMR: nuclear magnetic resonance
C1: one-carbon
ppm: parts per million
PMX: pemetrexed
P_2_O_5_: phosphorous pentoxide
PEG: polyethylene glycol
PDX: pralatrexate
PDB: Protein Data Bank
PCFT: proton-coupled folate transporter
PLP: pyridoxal phosphate
RTX: raltitrexed
RFC: reduced folate carrier
RP: reverse phase
NaOH: sodium hydroxide
SHMT2: serine hydroxymethyl transferase 2
THF: tetrahydrofolate
TLC: thin layer chromatography
TS: thymidylate synthase
TCA: trichloroacetic acid
TCEP: tris(2-chloroethyl) phosphate
TAMs: tumor-associated macrophages
β-ME: β-mercaptoethanol
UV: ultraviolet
