# Supplementary material for: Multitargeted 6-Substituted Thieno[2,3-d]pyrimidines as Folate Receptor-Selective Anticancer Agents that Inhibit Cytosolic and Mitochondrial One-Carbon Metabolism
Source: ACS Pharmacol Transl Sci. 2023 Apr 26;6(5):748–70. doi: 10.1021/acsptsci.3c00020 (PMC10186366; doi:10.1021/acsptsci.3c00020)
Supplement: Supplementary file 1 — pt3c00020_si_001.pdf [file pt3c00020_si_001.pdf]

SUPPORTING INFORMATION

Multi-targeted 6-substituted thieno[2,3-*d*]pyrimidines as folate receptor-selective anticancer agents that inhibit cytosolic and mitochondrial one-carbon metabolism

Nian Tong[θ£], Jennifer Wong-Roushar[θ¥], Adrianne Wallace-Povirk[θ‡], Yesha Shah[£], Morgan C. Nyman[¥], Jade M. Katinas[¥], Mathew Schneider[‡], Carrie O'Connor[‡], Xun Bao[δ], Seogho Kim[‡δ], Jing Li[‡δ], Zhanjun Hou[‡δ], Larry H. Matherly[‡£δ*], Charles E. Dann III[¥*], Aleem Gangjee[£*]

[£]Division of Medicinal Chemistry, Graduate School of Pharmaceutical Sciences, Duquesne University, Pittsburgh, PA 15282
[¥] Department of Chemistry, Indiana University, Bloomington, IN 47405
[‡]Department of Oncology, Wayne State University School of Medicine, Detroit, MI 48201
[£]Department of Pharmacology, Wayne State University School of Medicine, Detroit, MI 48201
[δ]Barbara Ann Karmanos Cancer Institute, Detroit, MI 48201

[θ]These authors contributed equally to this work.

*To whom correspondence should be addressed.

AUTHOR INFORMATION

Corresponding Authors

Aleem Gangjee, Phone: PhD, Division of Medicinal Chemistry, Graduate School of Pharmaceutical Sciences, Duquesne University, 600 Forbes Avenue, Pittsburgh, PA 15282; 412-396-6070; 412-396-5593 fax; gangjee@duq.edu
Charles E. Dann III, PhD, Department of Chemistry and Interdisciplinary Graduate Program in Biochemistry, Indiana University, Bloomington, IN 47405; 812-856-1704; 812-856-5710 fax; cedann@indiana.edu
Larry H. Matherly, PhD, Karmanos Cancer Institute, 4100 John R, Detroit, MI 48201; 313-578-4280; 313-578-4287 fax; matherly@karmanos.org

Table of Contents

Table S1. Docked Scores for 6-substituted thieno[2,3-*d*]pyrimidine antifolates. S3

Table S2. Data collection and refinement statistics for GARFTase crystal structures in complex with 6-substituted thieno[2,3-*d*]pyrimidines and β-GAR. S4

Figure S1. ^{1}H NMR spectra, HPLCs, and mass spectra of final compounds **3**-**9**. S5

Figure S2. D_2O exchange studies of representative example **9**. S12

Figure S3. Growth inhibition of KB human tumor cells by thieno[2,3-*d*]pyrimidine analogs and the protective effects of nucleosides, glycine and/or AICA. S13

Figure S4. Crystal structure of **4** bound in the folate binding pocket of GARFTase. S14

Figure S5. Crystal structure of **10** bound in the folate binding pocket of GARFTase. S15

Figure S6. Crystal structure of **9** bound in the folate binding pocket of GARFTase. S16

Figure S7. Crystal structure of **5** bound in the folate binding pocket of GARFTase. S17

Table S1. Docked Scores for 6-substituted thieno[2,3-*d*]pyrimidine antifolates

Docked Scores of 6-substituted thieno[2,3-*d*]pyrimidine antifolates in FRα, FRβ, GARFTase and AICARFTase				
**Compound**	**FRα (kcal/mol) PDB: 5IZQ**	**FRβ (kcal/mol) PDB:4KN2**	**GARFTase (kcal/mol) PDB:7JG0**	**AICARFTase (kcal/mol) PDB:1P4R**
**1**	-14.42	-14.33	-15.52	-12.95
**2**	-14.62	-15.14	-15.60	-12.49
**3**	-13.41	-12.90	-16.09	-12.88
**4**	-13.68	-12.77	-15.40	-12.71
**5**	-13.64	-14.76	-15.68	-12.64
**6**	-14.50	-15.23	-15.69	-13.38
**7**	-14.51	-13.60	-16.05	-12.23
**8**	-16.26	-13.00	-15.68	-13.67
**9**	-14.14	-12.70	-16.05	-13.51
**10**	-14.90	-14.01	-16.05	-13.08
**11**	-15.98	-14.97	-16.01	-12.93
**PMX**	-14.20	-13.28	-14.45	-11.36

Table S2. Data collection and refinement statistics for GARFTase crystal structures in complex with 6-substituted thieno[2,3-*d*]pyrimidines and β-GAR. Values in parentheses are for the highest resolution shell.

	**GARFTase Cpd 4 (AGF132) PDB 8FDY**	**GARFTase Cpd 10 (AGF271) PDB 8FDX**	**GARFTase Cpd 9 (AGF302) PDB 8FDZ**	**GARFTase Cpd 5 (AGF305) PDB 8EF0**
**Data Collection**				
Space group	$P3_221$	$P3_221$	$P3_221$	$P3_221$
Cell dimensions				
a=b, c (Å)	74.66, 100.79	74.12, 99.76	74.98, 99.73	74.88, 100.62
Molecule per a.s.u	1	1	1	1
Resolution	39.75 - 2.06 (2.12 - 2.06)	39.39 - 2.07 (2.13 - 2.07)	39.55 - 2.48 (2.58 - 2.48)	39.75 - 2.22 (2.29 - 2.22)
I/σI	11.0 (0.7)	10.3 (0.7)	9.5 (0.9)	7.1 (0.9)
Completeness (%)	100.0 (100.0)	99.0 (90.9)	100.0 (99.8)	100.0 (100.0)
Redundancy	10.5 (10.4)	8.3 (3.3)	10.0 (7.4)	9.7 (7.0)
R_{meas}	0.209 (3.265)	0.161 (1.871)	0.276 (2.362)	0.505 (2.516)
R_{pim}	0.064 (1.008)	0.054 (0.971)	0.087 (0.850)	0.161 (0.929)
CC1/2	0.997 (0.383)	0.997 (0.412)	0.992 (0.365)	0.969 (0.362)
**Refinement**				
Resolution (Å)	39.75 - 2.06 (2.13 - 2.06)	39.39 - 2.07 (2.14 - 2.07)	39.55 - 2.48 (2.57 - 2.48)	39.75 - 2.22 (2.30 - 2.22)
No. reflections				
Used for refinement	19616	18477	11287	15779
Used for R_{free} calculation	960	953	597	796
R_{factor} (%)	21.1	20.22	20.5	20.5
R_{free} (%)	25.5	24.0	24.9	25.2
No. atoms				
Protein	1495	1514	1491	1503
Ligand	51	49	51	52
Water molecules	60	91	53	106
B-factors (Å^2, average)				
Overall	46.8	41.5	43.7	33.1
Protein	45.8	41.2	43.5	32.8
Ligand	75.6	48.3	50.7	38.0
Water	47.1	42.7	42.3	33.9
r.m.s. deviations				
Bond length (Å)	0.005	0.007	0.008	0.007
Bond angle (°)	0.91	1.03	1.07	1.06
MolProbity clash score	3.21	4.14	5.79	3.83

Figure S1. ^{1}H NMR spectra, HPLCs and mass spectra of final compounds **3-9**.

Compound 3

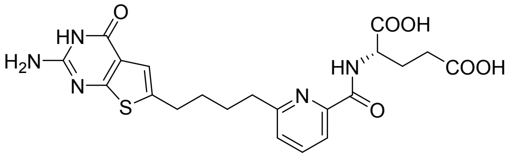

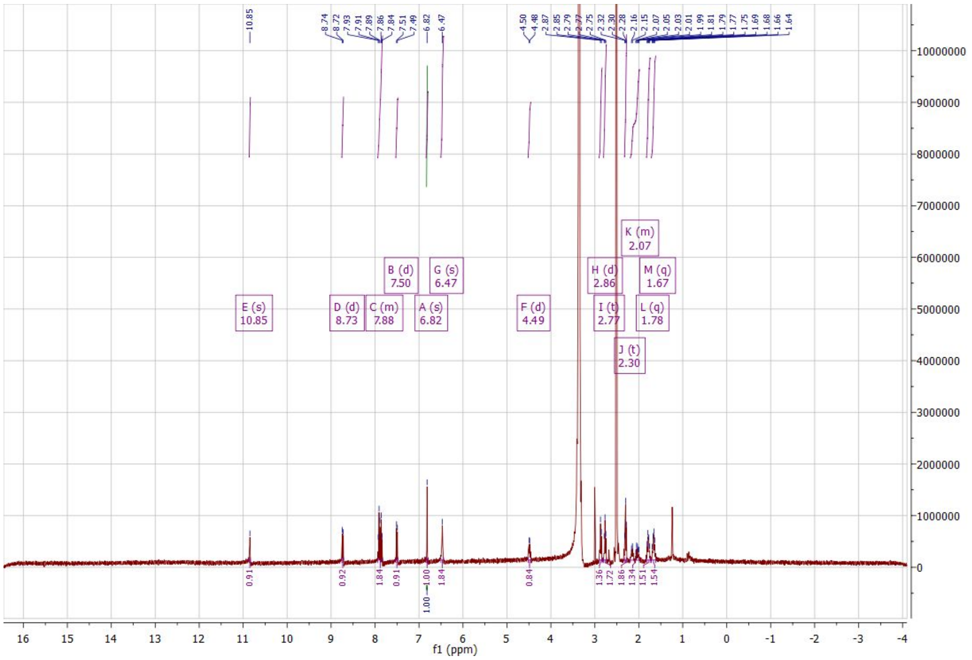

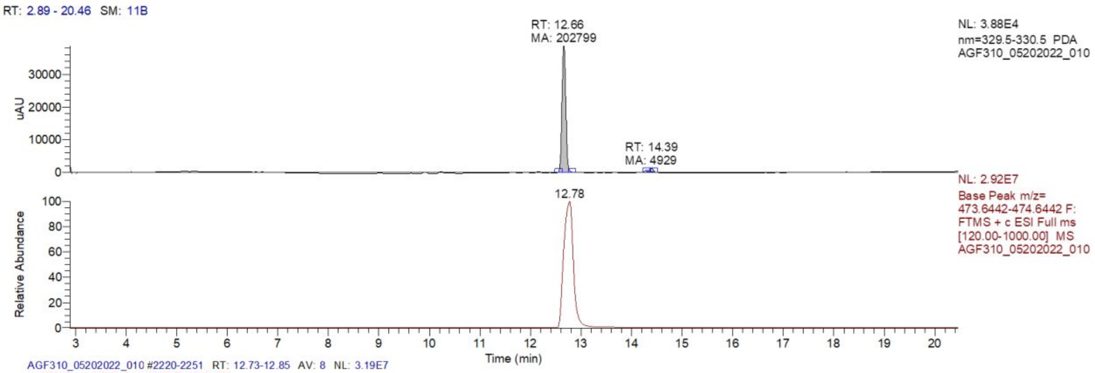

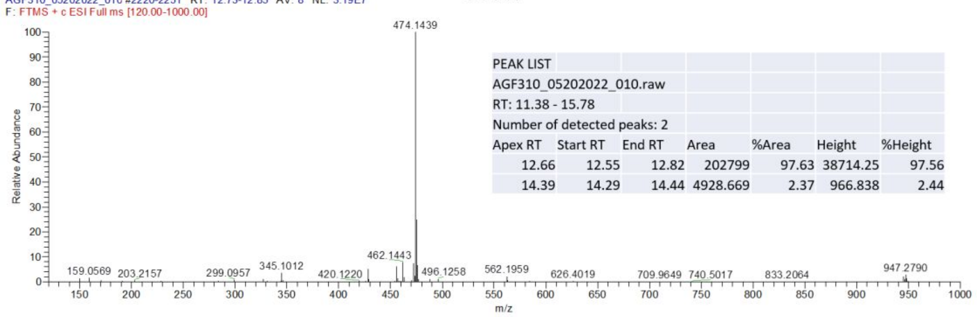

PEAK LIST
AGF310_05202022_010.raw
RT: 11.38 - 15.78
Number of detected peaks: 2

Apex RT	Start RT	End RT	Area	%Area	Height	%Height
12.66	12.55	12.82	202799	97.63	38714.25	97.56
14.39	14.29	14.44	4928.669	2.37	966.838	2.44

Compound 4

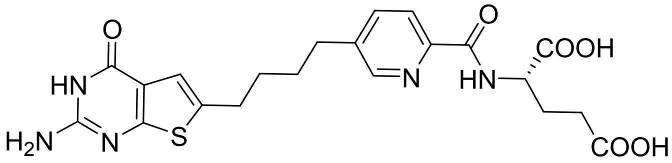

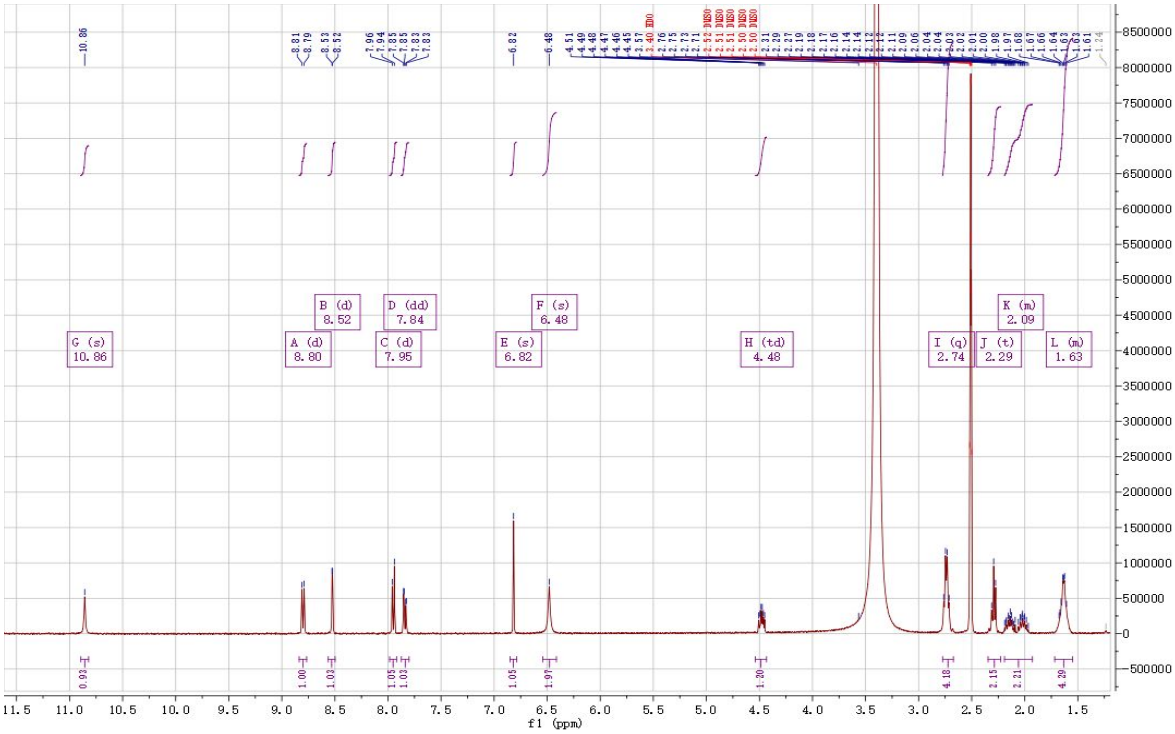

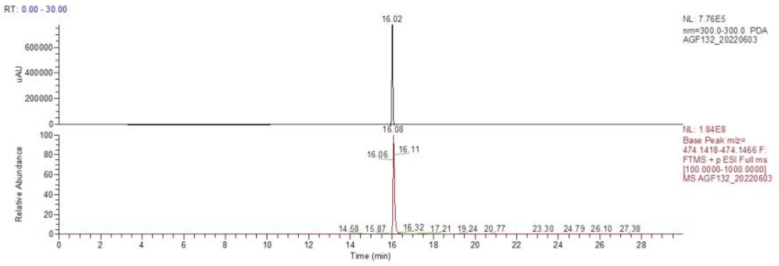

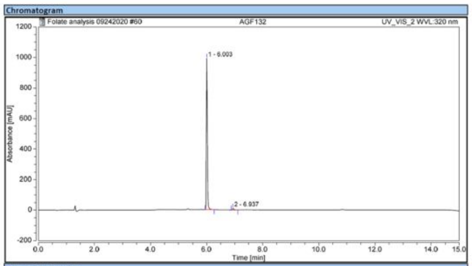

Integration Results

No.	Peak Name	Retention Time	Area	Height	Relative Area	Relative Height	Amount
		min	mAU*min	mAU	%	%	n.a.
1		6.003	37.852	990.565	98.54	98.91	n.a.
2		6.937	0.563	10.898	1.46	1.09	n.a.
Total:			38.415	1001.463	100.00	100.00	

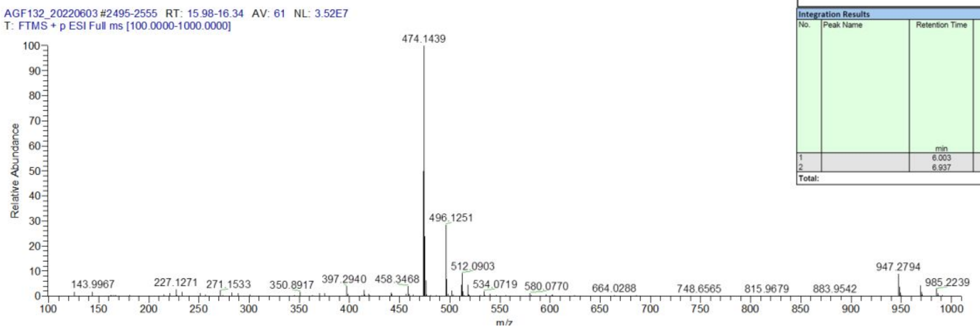

Compound 5

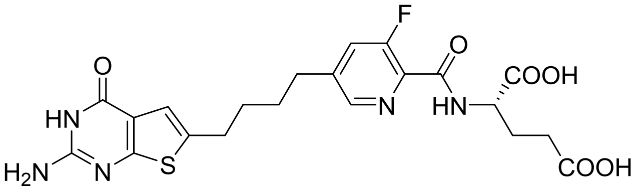

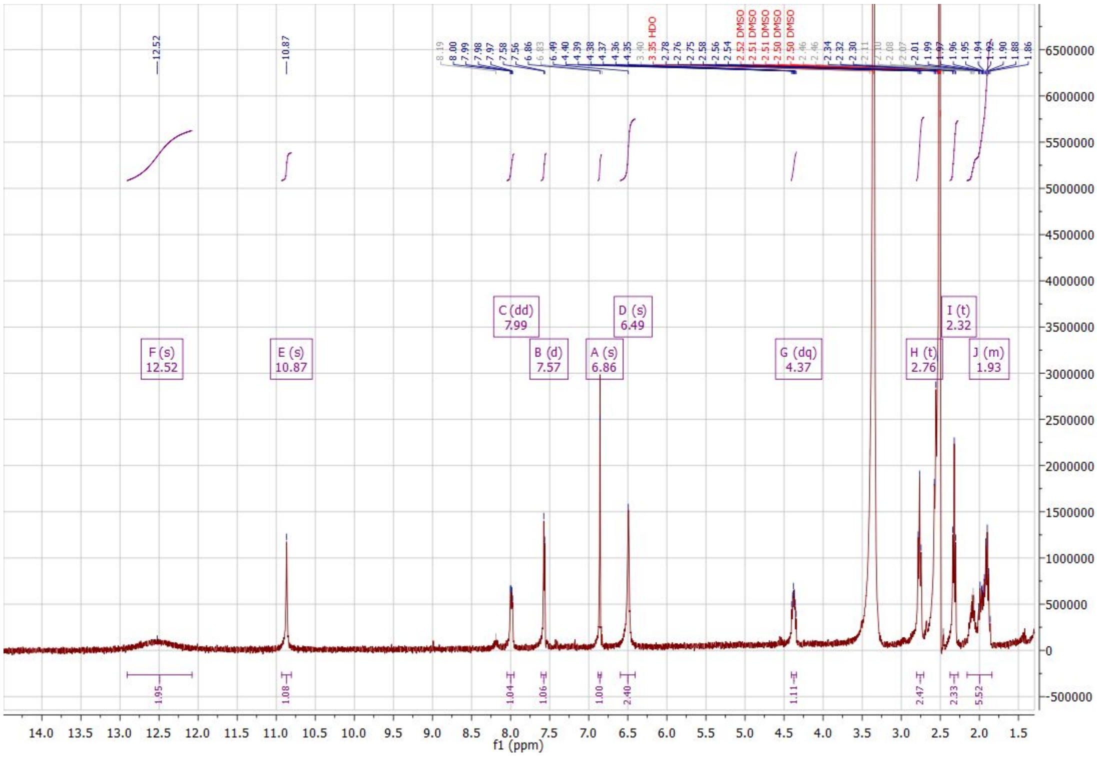

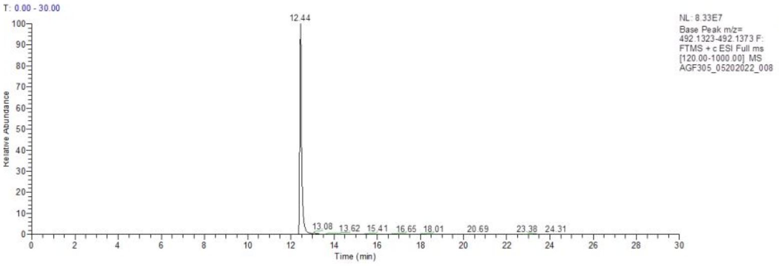

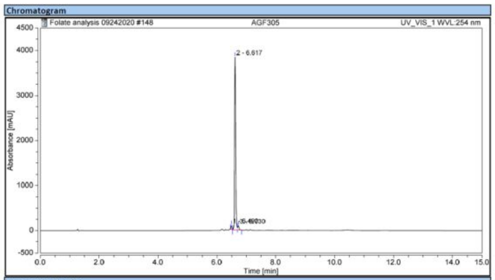

Integration Results

No.	Peak Name	Retention Time	Area	Height	Relative Area	Relative Height	Amount
		min	mAU*min	mAU	%	%	n.a.
1		6.490	3.054	92.461	1.71	2.30	n.a.
2		6.617	171.740	3838.286	95.91	95.31	n.a.
3		6.730	4.267	96.416	2.38	2.39	n.a.
Total:			179.061	4027.162	100.00	100.00	

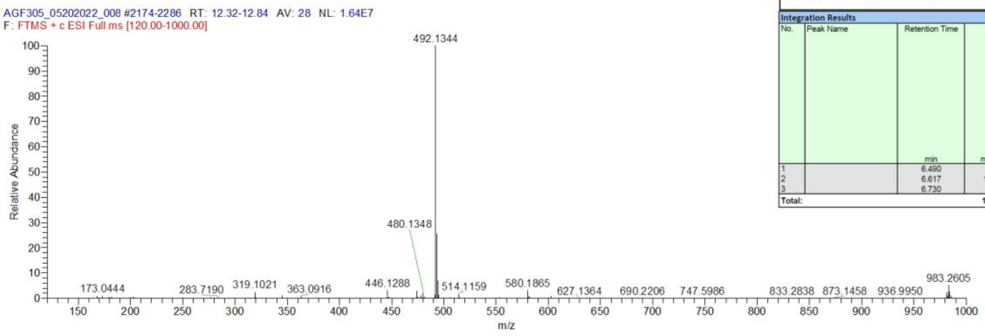

Compound 6

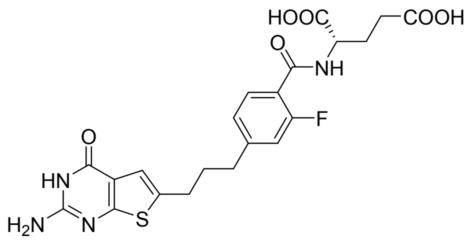

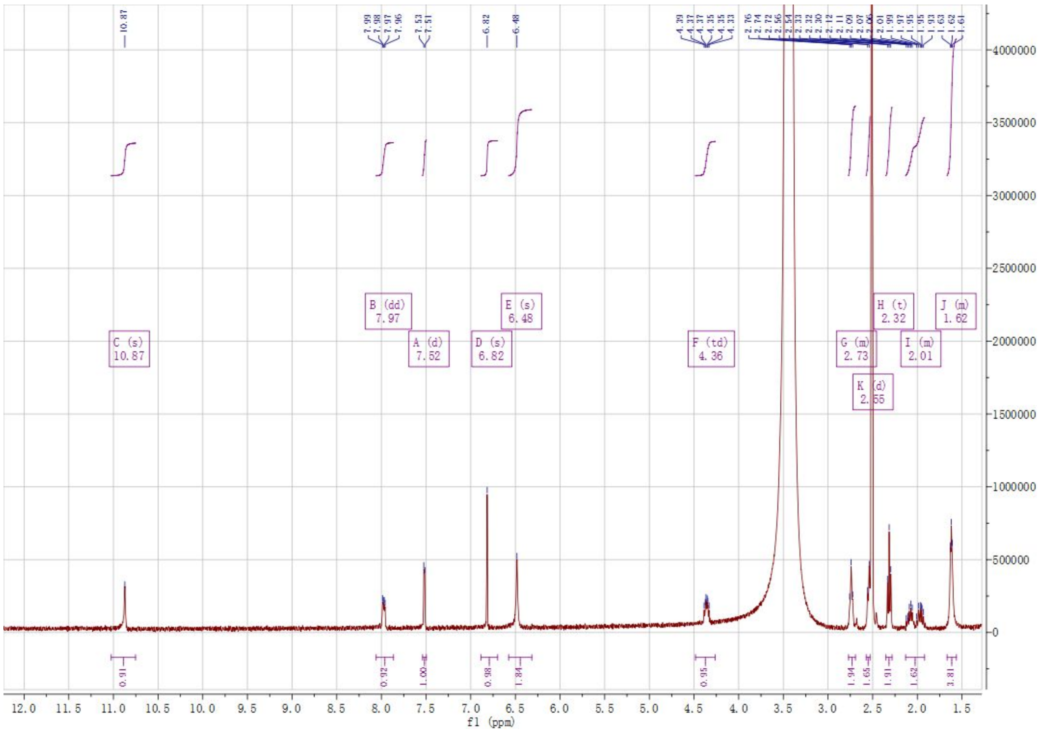

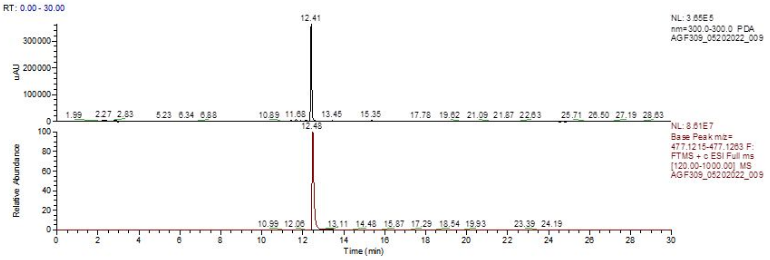

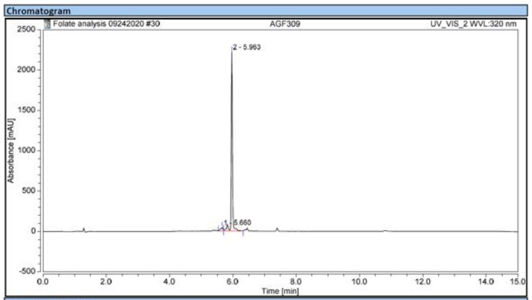

Integration Results

No.	Peak Name	Retention Time	Area	Height	Relative Area	Relative Height	Amount
		min	mAU*min	mAU	%	%	n.a.
1		5.660	3.421	47.404	3.50	2.09	n.a.
2		5.963	94.437	2222.280	96.50	97.91	n.a.
Total:			97.859	2269.684	100.00	100.00	

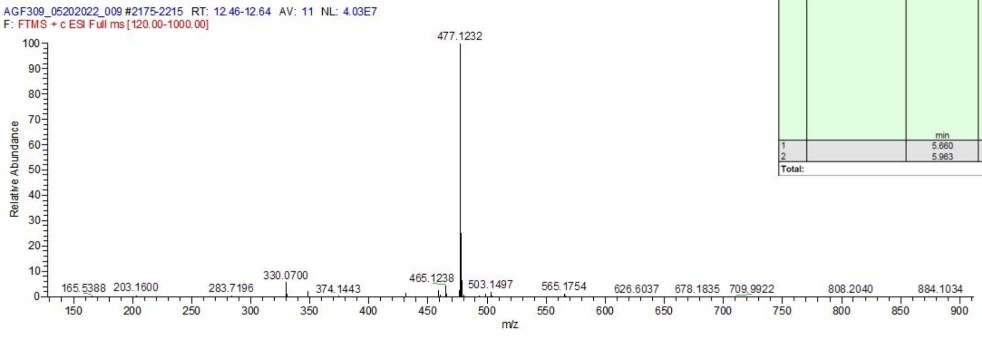

Compound 7

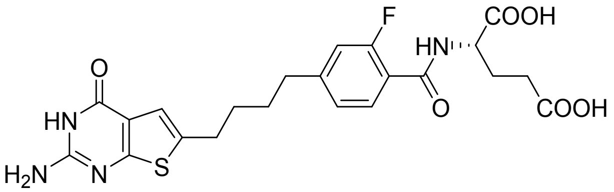

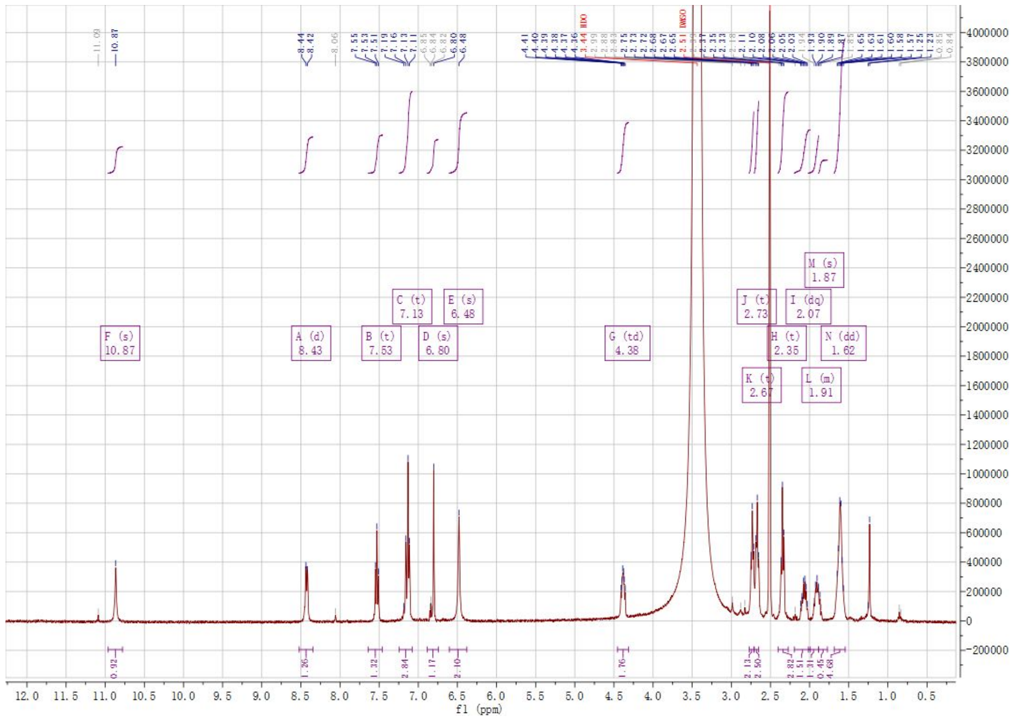

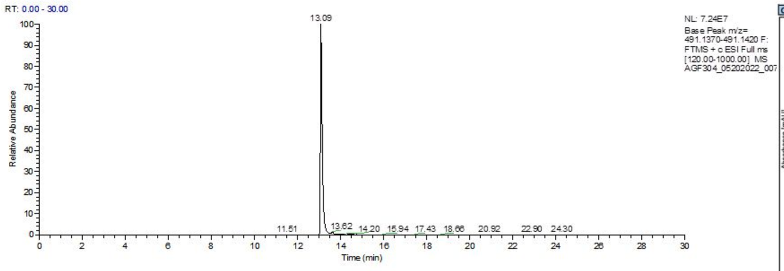

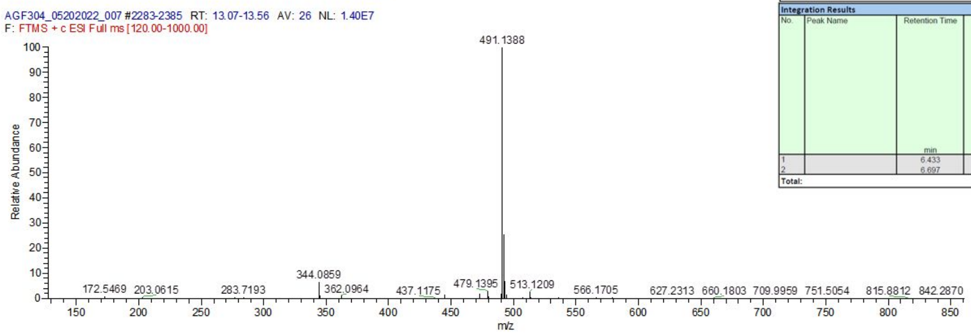

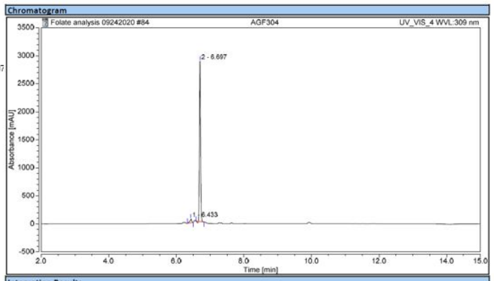

Integration Results

No.	Peak Name	Retention Time	Area	Height	Relative Area	Relative Height	Amount
		min	mAU*min	mAU	%	%	n.a.
1		6.433	4.383	72.833	3.86	2.48	n.a.
2		6.697	109.243	2863.240	96.14	97.52	n.a.
Total:			113.625	2936.073	100.00	100.00	

Compound 8

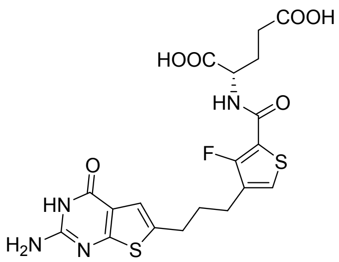
COOH
HOOC
HN
O
F
S
O
HN
H2N
N
S

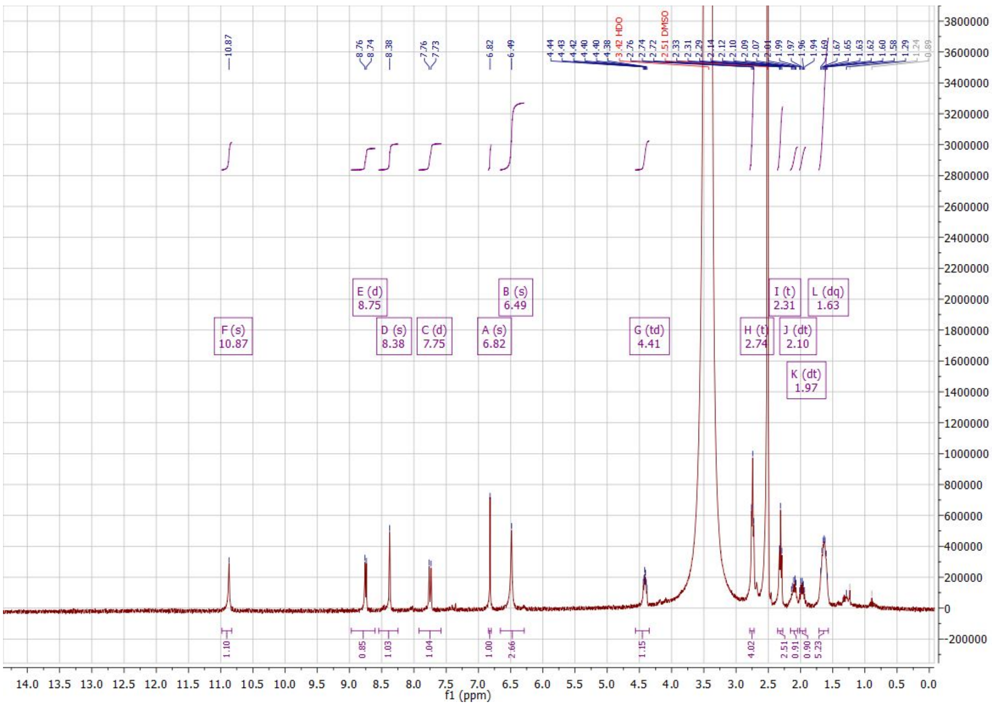
F (s)
10.87
E (d)
8.75
D (s)
8.38
C (d)
7.75
A (s)
6.82
B (s)
6.49
G (td)
4.41
H (t)
2.74
I (t)
2.31
J (dt)
2.10
K (dt)
1.97
L (dq)
1.63
3.42 HDO
2.51 DMSO
f1 (ppm)

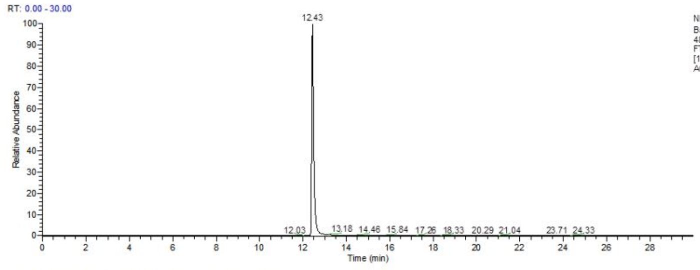
RT: 0.00 - 30.00
12.43
Relative Abundance
Time (min)
NL: 5.99E7
Base Peak m/z=
483.0779-483.0827 F:
FTMS + c ESI Full ms
[120.00-1000.00] MS
AGF298_05202022_004

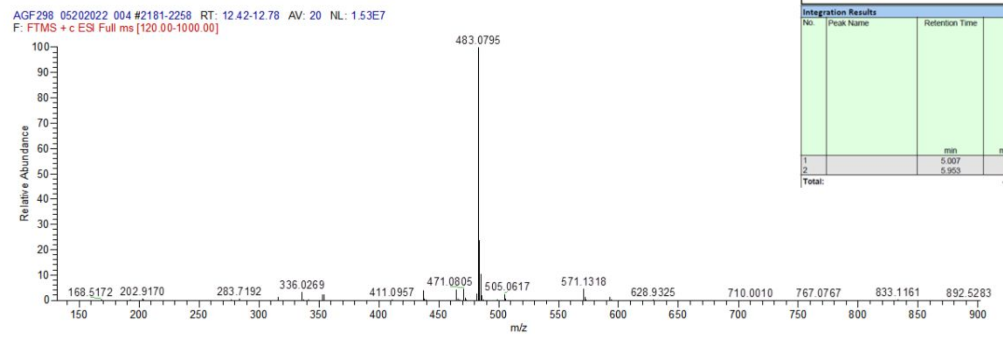
AGF298_05202022_004 #2181-2258 RT: 12.42-12.78 AV: 20 NL: 1.53E7
F: FTMS + c ESI Full ms [120.00-1000.00]
483.0795
471.0805
505.0617
571.1318
Relative Abundance
m/z

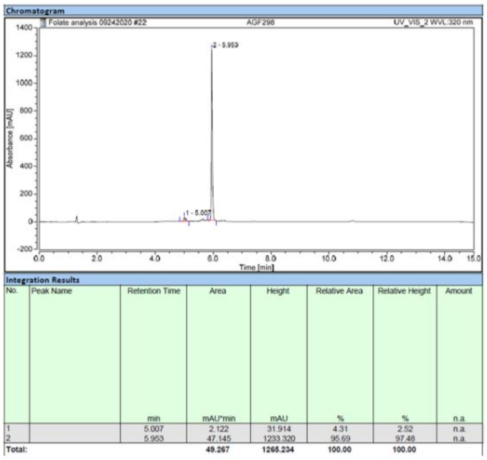
Chromatogram
Folate analysis 09242020 #22
AGF298
UV_VIS_2 WVL:320 nm
Absorbance [mAU]
Time [min]
Integration Results
No. | Peak Name | Retention Time | Area | Height | Relative Area | Relative Height | Amount
min | mAU*min | mAU | % | % | n.a.
1 | 5.007 | 2.122 | 31.914 | 4.31 | 2.52 | n.a.
2 | 5.953 | 47.145 | 1233.320 | 95.69 | 97.48 | n.a.
Total: | 49.267 | 1265.234 | 100.00 | 100.00

Compound 9

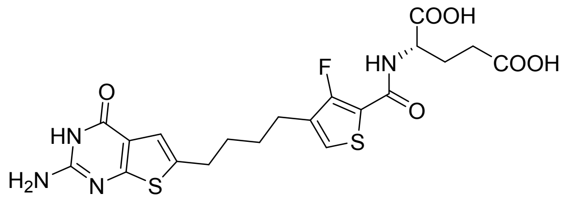

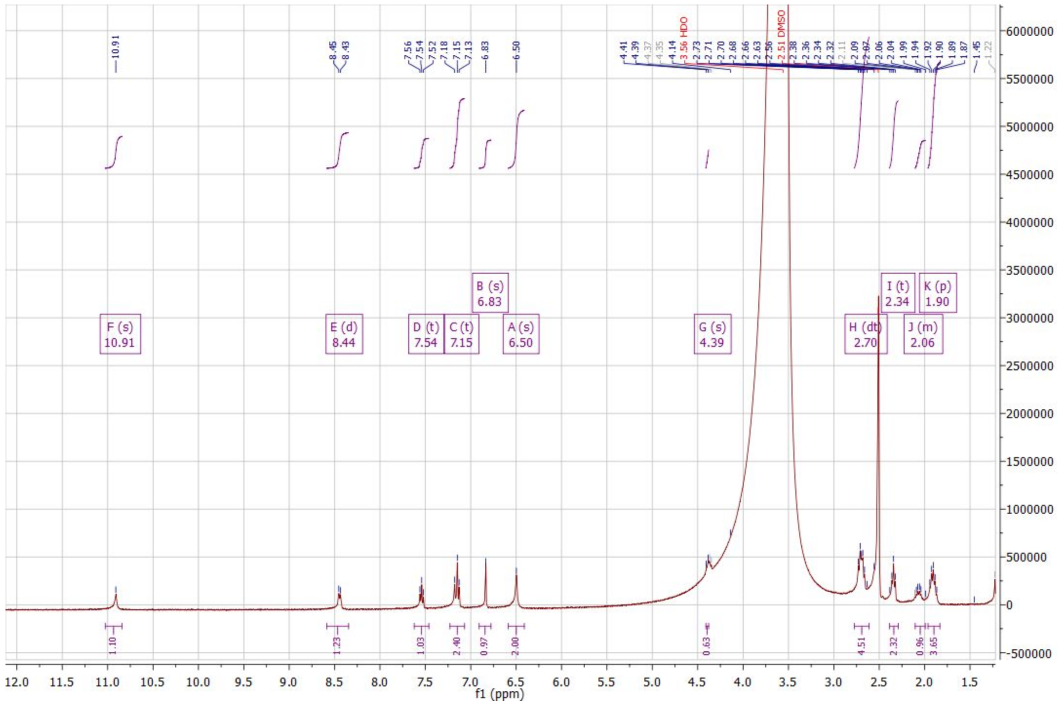

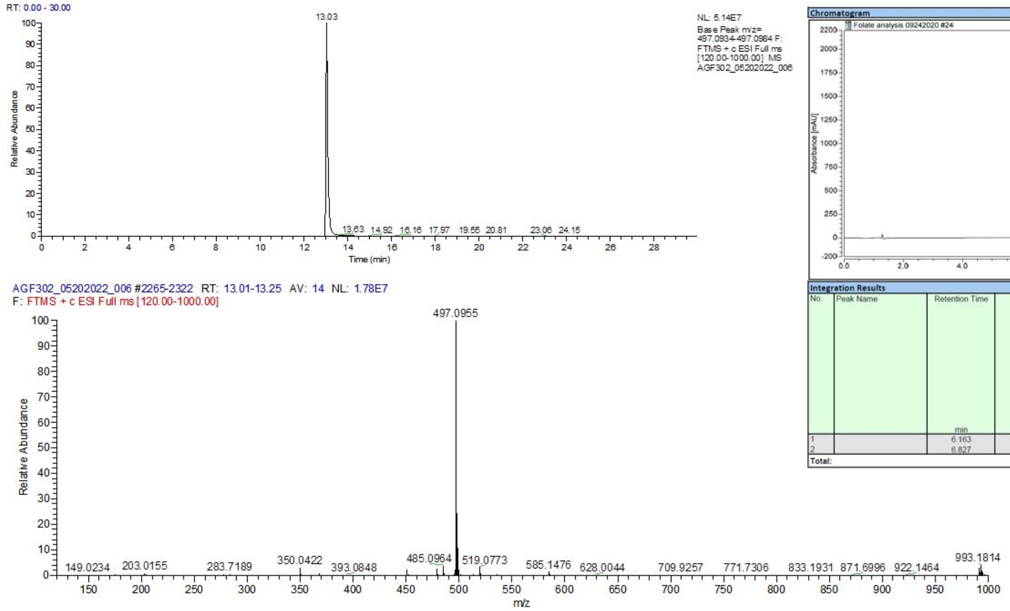

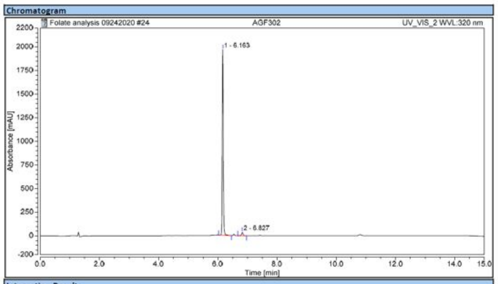

Integration Results

No.	Peak Name	Retention Time	Area	Height	Relative Area	Relative Height	Amount
		min	mAU*min	mAU	%	%	n.a.
1		6.163	76.364	1966.697	97.78	98.21	n.a.
2		6.827	1.737	35.870	2.22	1.79	n.a.
Total:			78.100	2002.567	100.00	100.00	

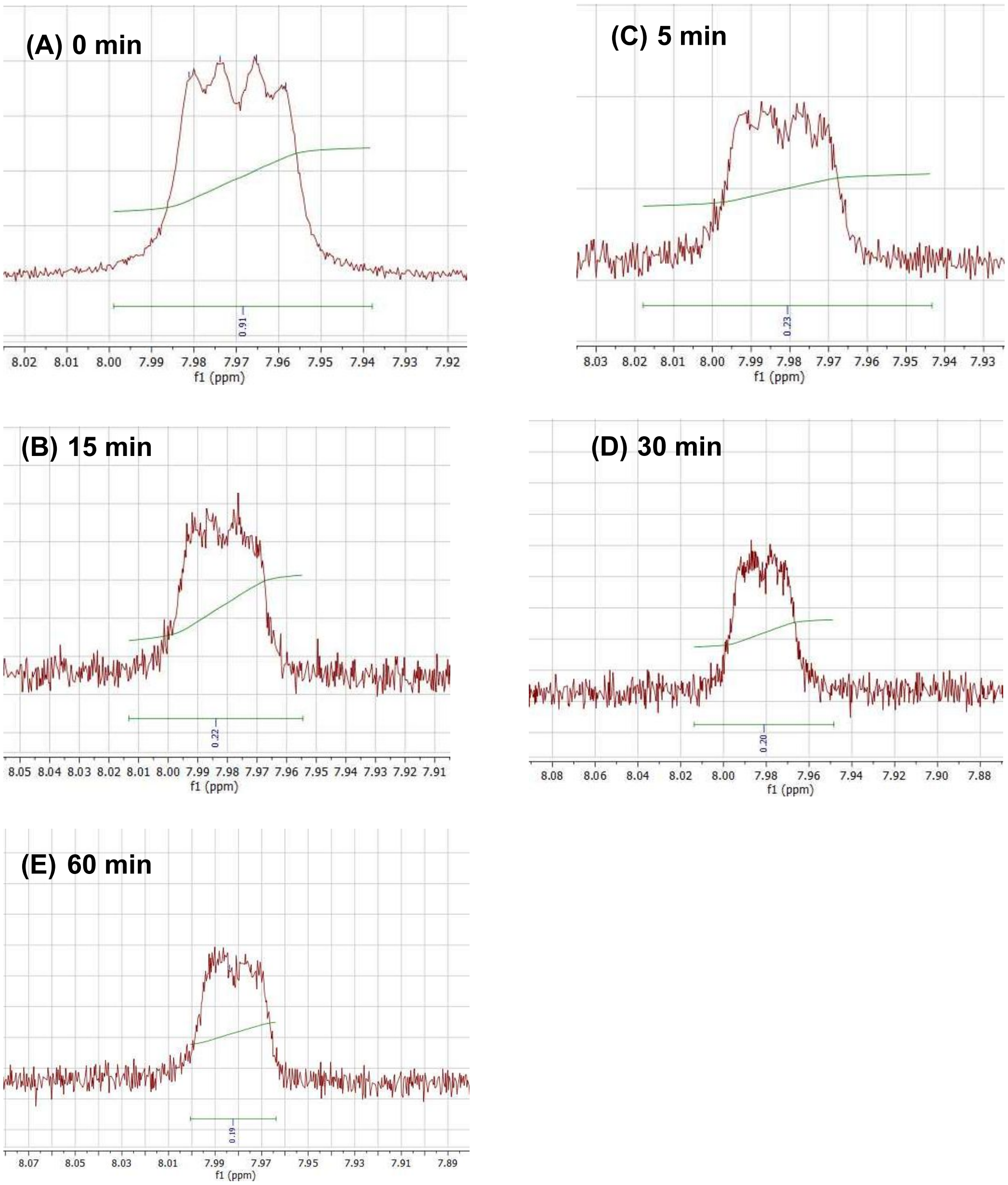

Figure S2. D_2O exchange studies of representative example **9**. The integration of NH signal over 60 minutes upon exchange of 1 mg compound of **9** in 0.7 mL DMSO-d_6 with 0.01 mL D_2O. (A) Integration of NH signal before exchange with D_2O (B) Integration of NH signal 5 minutes after exchange with D_2O. (C) Integration of NH signal 15 minutes after exchange with D_2O. (D) Integration of NH signal 30 minutes after exchange with D_2O. (E) Integration of NH signal 60 minutes after exchange with D_2O.

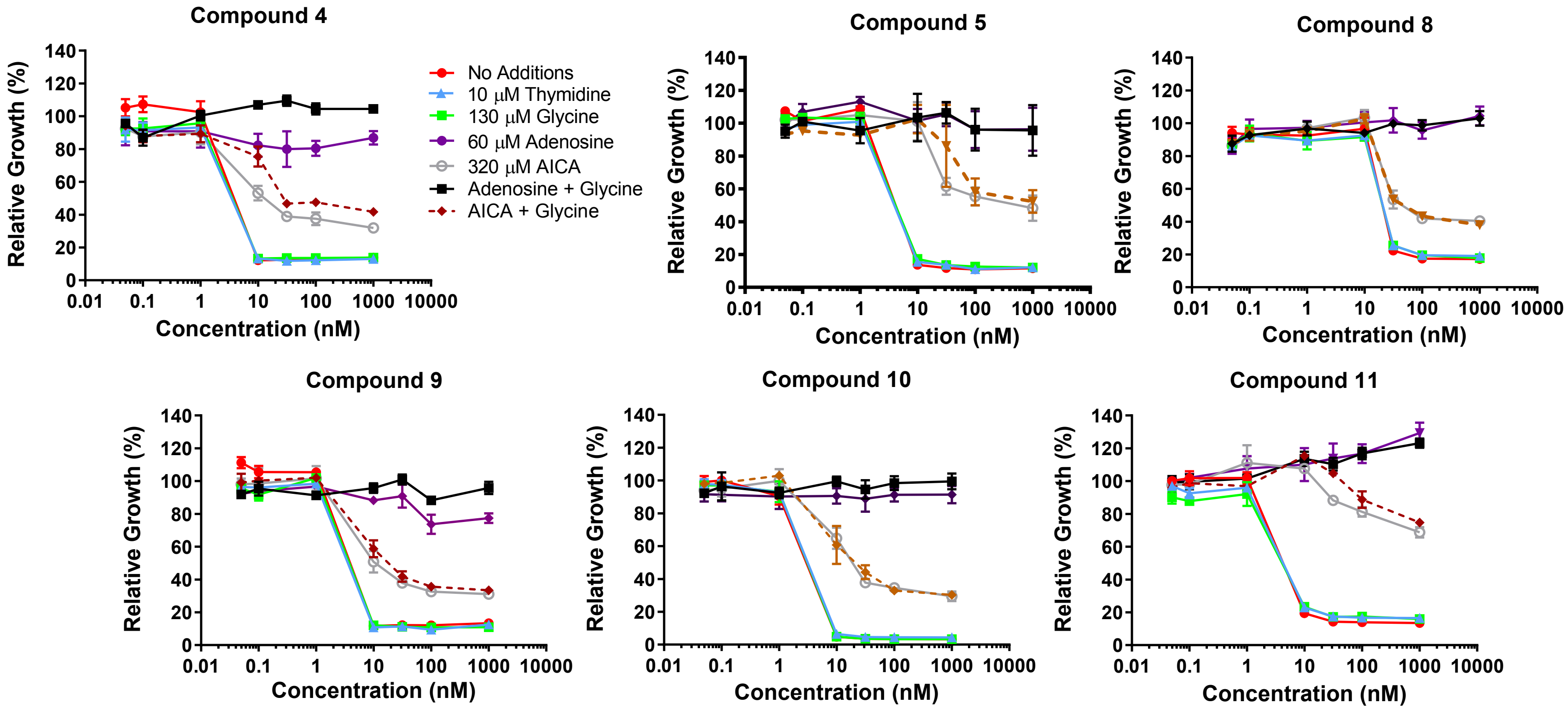

Figure S3. Growth inhibition of KB human tumor cells by thieno[2,3-*d*]pyrimidine analogs and the protective effects of nucleosides, glycine and/or AICA. KB cells were plated (4000 cells/well) in glycine-, nucleoside and folate-free RPMI 1640 medium with 10% dialyzed fetal bovine serum, antibiotics, *L*-glutamine, and 2 nM leucovorin over a range of drug concentrations, in the presence of adenosine (60 µM), glycine (160 µM), thymidine (10 µM), or AICA (320 µM). Combined glycine plus adenosine or AICA was also tested to identify potential mitochondrial C1 targeting. Cell proliferation was assayed with a fluorescence-based assay. Data are representative of at least triplicate experiments. Methods are described in the Experimental Section.

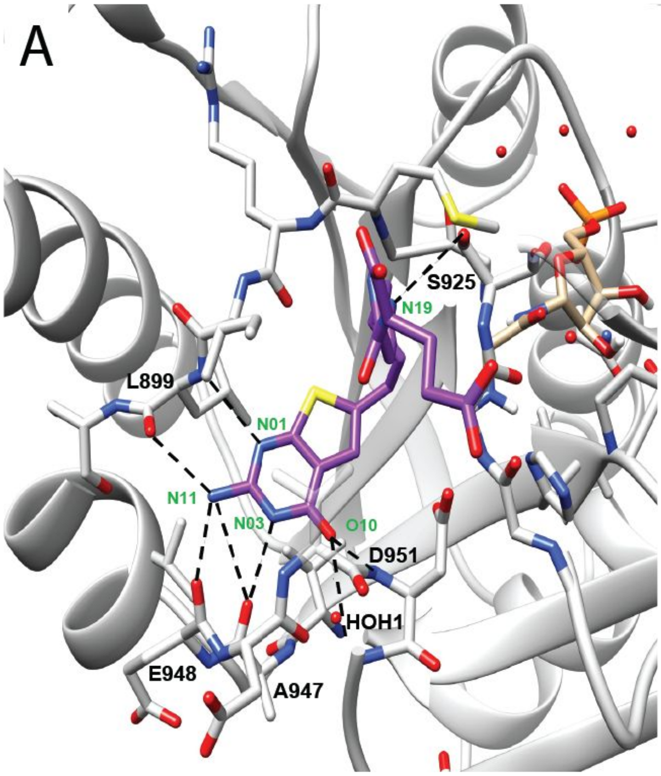

Atom 1	Atom 2	Distance (Å)
Leu899 N	AGF132 N01	3.23
Ala947 O	AGF132 N03	2.99
Leu899 O	AGF132 N11	3.12
Ala947 O	AGF132 N11	3.66
Glu948 O	AGF132 N11	3.85
Ser925 OG	AGF132 N19	3.08
HOH1	AGF132 O10	2.58
Asp951 N	AGF132 O10	3.00

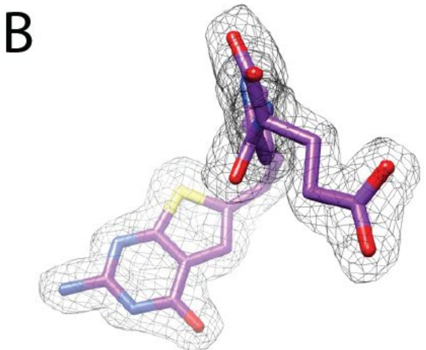

Figure S4. Crystal structure of compound 4 bound in the folate binding pocket of GARFTase. (A) Hydrogen-bond and charge-charge interactions between compound **4** and GARFTase are shown as dashed lines. Atoms that participate in longer distance H-bonding or charge-charge interactions are labeled in red. GARFTase is shown in ribbon except for interacting side-chain and backbone atoms, which are shown in stick. Distances between interacting atoms are shown in the table with distances greater than 3.2 Å for H-bonding and greater than 4.0 Å for salt-bridge interactions in red. (B) The structure is shown of compound **4** with the 2Fo - Fc map density in mesh contoured at 0.7 σ.

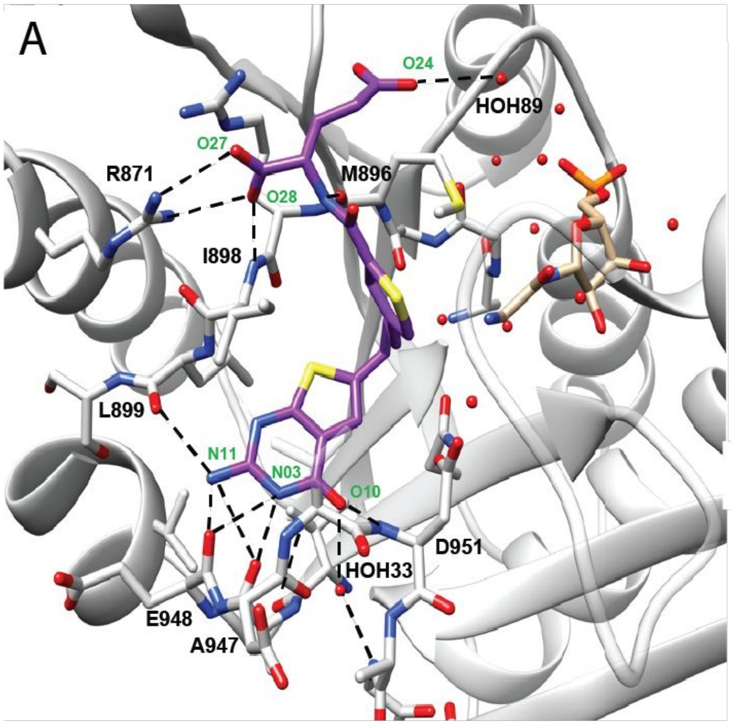

Atom 1	Atom 2	Distance (Å)
Leu 899 N	AGF271 N01	3.01
Ala 947 O	AGF271 N03	2.55
Glu 948 O	AGF271 N11	2.70
Leu 899 O	AGF271 N11	3.12
Ala 947 O	AGF271 N11	3.31
Met 896 O	AGF271 N19	2.68
Asp 951 N	AGF271 O10	2.90
HOH33	AGF271 O10	2.78
HOH89	AGF271 O24	3.36
Arg 871 NH2	AGF271 O27	3.16
Arg 871 NH1	AGF271 O28	3.19
Ile 898 N	AGF271 O28	2.93

B

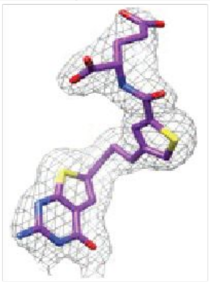

Figure S5. Crystal structure of compound 10 bound in the folate binding pocket of GARFTase. (A) Hydrogen-bond and charge-charge interactions between compound **10** and GARFTase are shown as dashed lines. Atoms that participate in longer distance H-bonding or charge-charge interactions are labeled in red. GARFTase is shown in ribbon except for interacting side-chain and backbone atoms, which are shown in stick. Distances between interacting atoms are shown in the table with distances greater than 3.2 Å for H-bonding and greater than 4.0 Å for salt-bridge interactions in red. (B) The structure is shown of compound **10** with the 2Fo - Fc map density in mesh contoured at 0.7 σ.

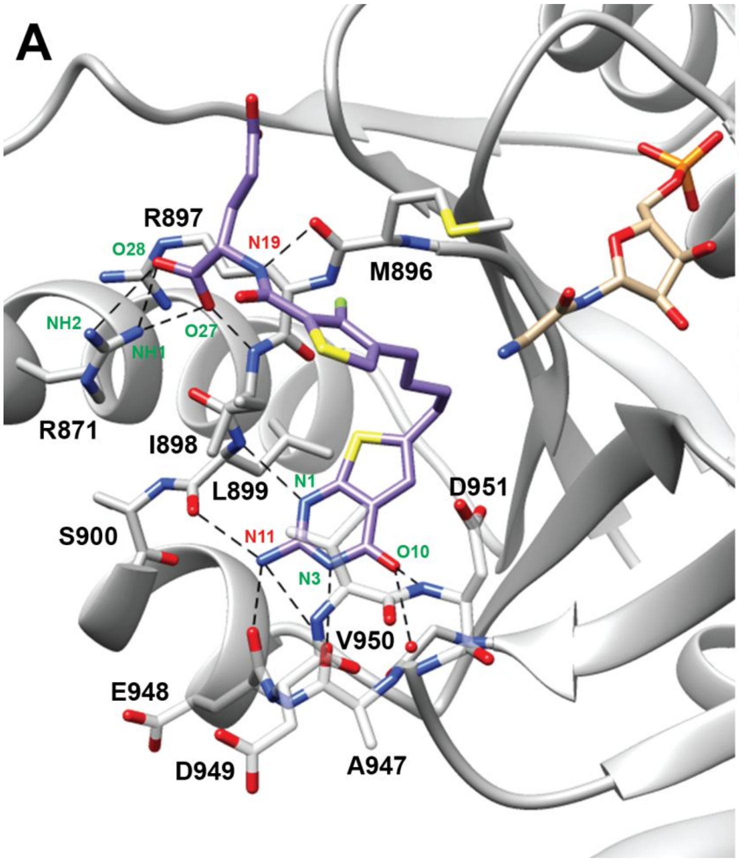

Atom 1	Atom 2	Distance (Å)
Leu 899 N	AGF302 N01	3.18
Ala 947 O	AGF302 N03	2.90
Glu 948 O	AGF302 N11	3.12
Leu 899 O	AGF302 N11	3.20
Ala 947 O	AGF302 N11	3.62
Met 896 O	AGF302 N19	3.48
HOH 1	AGF302 O10	2.71
Asp 951 N	AGF302 O10	3.14
Arg 871 NH1	AGF302 O27	3.00
Arg 871 NH2	AGF302 O27	3.22
Ile 898 N	AGF302 O28	2.60
Arg 871 NH1	AGF302 O28	2.89

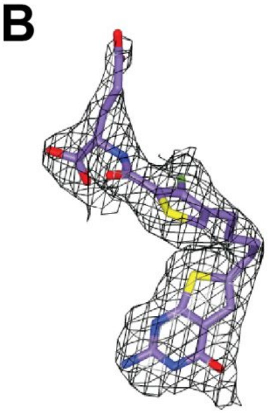

Figure S6. Crystal structure of compound 9 bound in the folate binding pocket of GARFTase. (A) Hydrogen-bond and charge-charge interactions between compound **9** and GARFTase are shown as dashed lines. Atoms that participate in longer distance H-bonding or charge-charge interactions are labeled in red. GARFTase is shown in ribbon except for interacting side-chain and backbone atoms, which are shown in stick. Distances between interacting atoms are shown in the table with distances greater than 3.2 Å for H-bonding and greater than 4.0 Å for salt-bridge interactions in red. (B) The structure is shown of compound **9** with the 2Fo - Fc map density in mesh contoured at 0.7 σ.

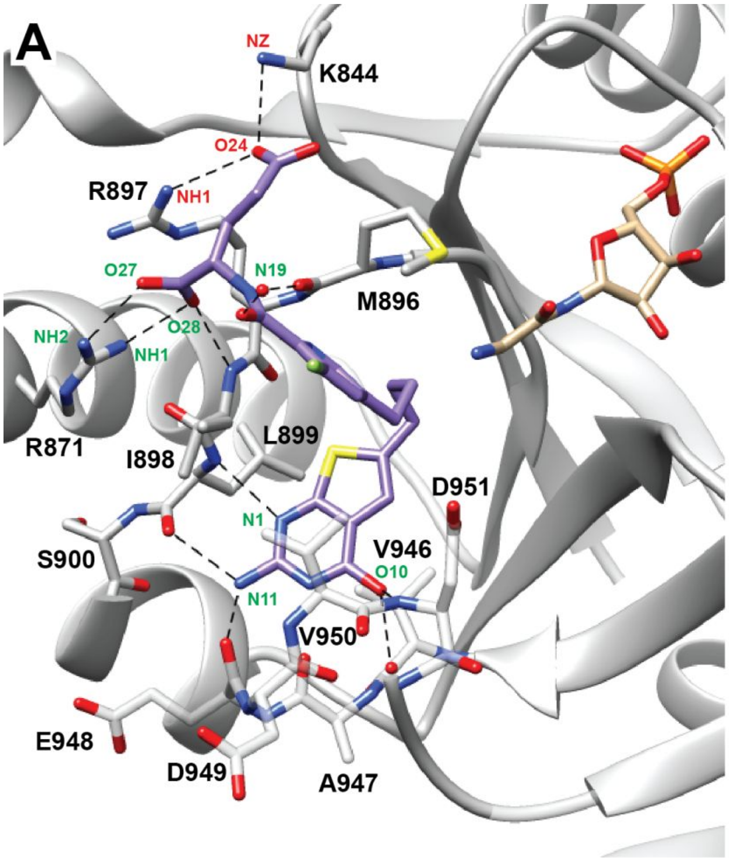

Atom 1	Atom 2	Distance (Å)
Leu 899 O	AGF305 N11	3.11
Glu 948 O	AGF305 N11	2.79
Met 896 O	AGF305 N19	2.86
Leu 899 N	AGF305 N19	3.15
Asp 951 N	AGF305 O10	2.80
HOH 1	AGF305 O10	2.52
HOH 2	AGF305 O18	3.03
Lys 844 NZ	AGF305 O24	3.57
Arg 897 NH1	AGF305 O24	3.35
Arg 871 NH2	AGF305 O27	2.83
Arg 871 NH1	AGF305 O28	3.08
Ile 898 N	AGF305 O28	3.00

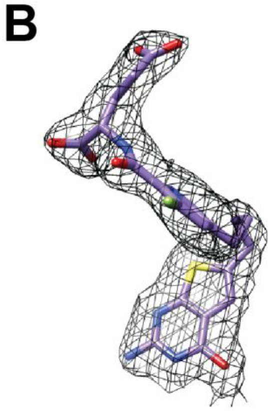

Figure S7. Crystal structure of compound 5 bound in the folate binding pocket of GARFTase. (A) Hydrogen-bond and charge-charge interactions between compound **5** and GARFTase are shown as dashed lines. Atoms that participate in longer distance H-bonding or charge-charge interactions are labeled in red. GARFTase is shown in ribbon except for interacting side-chain and backbone atoms, which are shown in stick. Distances between interacting atoms are shown in the table with distances greater than 3.2 Å for H-bonding and greater than 4.0 Å for salt-bridge interactions in red. (B) The structure is shown of compound **5** with the 2Fo - Fc map density in mesh contoured at 0.7 σ.
